# Confidence interval of risk difference by different statistical methods and its impact on the study conclusion in antibiotic non-inferiority trials

**DOI:** 10.1186/s13063-021-05686-8

**Published:** 2021-10-16

**Authors:** Anthony D. Bai, Adam S. Komorowski, Carson K. L. Lo, Pranav Tandon, Xena X. Li, Vaibhav Mokashi, Anna Cvetkovic, Aidan Findlater, Laurel Liang, George Tomlinson, Mark Loeb, Dominik Mertz, the McMaster Infectious Diseases Fellow Research Group

**Affiliations:** 1 Division of Infectious Diseases, Queen’s University, Kingston, ON Canada; 2 Health Research Methodology Program, McMaster University, Hamilton, ON Canada; 3 Division of Medical Microbiology, McMaster University, Hamilton, ON Canada; 4 Division of Infectious Diseases, McMaster University, Hamilton, ON Canada; 5 Global Health Office, McMaster University, Hamilton, ON Canada; 6 Leslie Dan Faculty of Pharmacy, University of Toronto, Toronto, ON Canada; 7 Department of Medicine, University Health Network and Mount Sinai Hospital, Toronto, ON Canada; 8 Institute of Health Policy Management and Evaluation, University of Toronto, Toronto, ON Canada

**Keywords:** Non-inferiority trials, Confidence interval, Statistics, Risk differences

## Abstract

**Background:**

Numerous statistical methods can be used to calculate the confidence interval (CI) of risk differences. There is consensus in previous literature that the Wald method should be discouraged. We compared five statistical methods for estimating the CI of risk difference in terms of CI width and study conclusion in antibiotic non-inferiority trials.

**Methods:**

In a secondary analysis of a systematic review, we included non-inferiority trials that compared different antibiotic regimens, reported risk differences for the primary outcome, and described the number of successes and/or failures as well as patients in each arm. For each study, we re-calculated the risk difference CI using the Wald, Agresti-Caffo, Newcombe, Miettinen-Nurminen, and skewness-corrected asymptotic score (SCAS) methods. The CIs by different statistical methods were compared in terms of CI width and conclusion on non-inferiority. A wider CI was considered to be more conservative.

**Results:**

The analysis included 224 comparisons from 213 studies. The statistical method used to calculate CI was not reported in 134 (59.8%) cases. The median (interquartile range IQR) for CI width by Wald, Agresti-Caffo, Newcombe, Miettinen-Nurminen, and SCAS methods was 13.0% (10.8%, 17.4%), 13.3% (10.9%, 18.5%), 13.6% (11.1%, 18.9%), 13.6% (11.1% and 19.0%), and 13.4% (11.1%, 18.9%), respectively. In 216 comparisons that reported a non-inferiority margin, the conclusion on non-inferiority was the same across the five statistical methods in 211 (97.7%) cases. The differences in CI width were more in trials with a sample size of 100 or less in each group and treatment success rate above 90%. Of the 18 trials in this subgroup with a specified non-inferiority margin, non-inferiority was shown in 17 (94.4%), 16 (88.9%), 14 (77.8%), 14 (77.8%), and 15 (83.3%) cases based on CI by Wald, Agresti-Caffo, Newcombe, Miettinen-Nurminen, and SCAS methods, respectively.

**Conclusions:**

The statistical method used to calculate CI was not reported in the majority of antibiotic non-inferiority trials. Different statistical methods for CI resulted in different conclusions on non-inferiority in 2.3% cases. The differences in CI widths were highest in trials with a sample size of 100 or less in each group and a treatment success rate above 90%.

**Trial registration:**

PROSPERO CRD42020165040. April 28, 2020.

**Supplementary Information:**

The online version contains supplementary material available at 10.1186/s13063-021-05686-8.

## Introduction

In most non-inferiority randomized controlled trials (RCTs), the primary outcome is reported as the risk difference between the treatment and control arms, which is a difference in proportions. The conclusion of whether a treatment is non-inferior is based on the confidence interval (CI) limit of this risk difference in relation to the pre-specified non-inferiority margin [1].

Numerous statistical methods can be used to calculate the CI for difference in proportions. The most commonly used and simplest method is the Wald CI, which is based on the asymptotic normal distribution [2]. Four other statistical methods were shown to perform better than the Wald method in terms of coverage probabilities in prior studies [3–7]. Agresti and Caffo proposed a modification by adding one success and one failure in each sample followed by calculation of the Wald CI [4]. In contrast, Newcombe used the Wilson score method for the single proportion and combined the intervals for the proportion in each treatment arm using the square and add method to calculate the CI [5]. Another method is the Miettinen and Nurminen method, which is based on restricted maximum likelihood estimation [8]. Lastly, the skewness-corrected asymptotic score method (SCAS) originated from the Gart and Nam method [9] and “re-defined the skewness correction using Miettinen and Nurminen’s contrast-based test statistics” [10]. The SCAS was shown to have superior equal-tailed coverage properties making it particularly suitable for use in one-sided non-inferiority testing [10]. Hereafter, these five statistical methods will be referred to as the Wald, Agresti-Caffo, Newcombe, Miettinen-Nurminen, and SCAS methods.

Although the CI is essential for the interpretation of results in non-inferiority RCTs, there is no consensus on the optimal statistical method to use. For example, the US Food and Drug Administration (FDA) guidance for industry document on non-inferiority RCTs does not specify any statistical method to construct the CI [11]. Prior studies have compared coverage probabilities for different CI methods [3–7]. According to these studies, the Wald method performs poorly compared to Agresti-Caffo, Newcombe, Miettinen-Nurminen, and SCAS methods [3, 5, 7]. There are still two unanswered questions when applying this to real non-inferiority trials. First, it is unclear if the recommendation of avoiding the Wald method has been followed in published non-inferiority trials. Second, non-inferiority trials typically have large and equal sample sizes above 30 in each group where the coverage probabilities of different CI statistical methods were shown to be more similar to one another [3]. Within these parameters of the non-inferiority trials, it is unclear if there are still significant differences in CI widths by different statistical methods that are large enough to change the study conclusion on non-inferiority for a given trial.

We recently performed a systematic review on antibiotic non-inferiority RCTs [12]. In this secondary analysis, we aimed to describe the reported statistical methods used to calculate the CI of risk differences in these non-inferiority RCTs as well as compare the five common CI statistical methods in terms of the CI width and consequent conclusion on non-inferiority.

## Methods

This was a secondary analysis of a previously conducted systematic review of antibiotic non-inferiority RCTs [12]. The full methodology of the original systematic review (PROSPERO registration number CRD42020165040) is described in the original journal publication [12].

### Inclusion criteria

The original systematic review included non-inferiority RCTs published up to November 22, 2019, comparing two or more systemic antibiotic regimens in the treatment of bacterial infections for humans. To be included in this secondary analysis, the primary outcome must be based on an absolute risk difference and the study must report the number of successes and/or failures as well as the number of patients in each arm.

### Statistical methods for CI

We calculated the absolute risk differences as the success rate in the treatment arm minus the success rate in the control arm. For studies that reported failure rates, we converted the failure rates to success rates by subtracting the number of failures from the number of patients in each arm. We then used these success rates to calculate the absolute risk differences. This is to ensure consistent orientation and meaning of positive and negative absolute risk differences. For studies that reported both intention-to-treat and per protocol analyses, we used the primary analysis. If both were viewed as co-primary analyses, we used the results from the intention-to-treat analysis.

We used the number of successes and total number of patients in the treatment and control arms to calculate the two-sided 95% CI for the absolute risk difference using the Wald, Agresti-Caffo, Newcombe, Miettinen-Nurminen, and SCAS methods. The calculation for each method is described in detail elsewhere [3, 7]. We chose the two-sided 95% CI, because it is the most commonly used CI width that is also recommended in the FDA industry guidance document for non-inferiority trials [11].

### Comparison of CI statistical methods

We compared the five statistical methods in terms of the CI width. A wider CI would be more conservative, because it would be less likely to exclude the non-inferiority margin. Note that this definition of conservatism differs from the conventional and more accurate definition of a conservative CI being one that ensures the coverage probability is at least the nominal confidence level [5].

We used the calculated two-sided 95% CI by each method to conclude non-inferiority based on the lower CI limit relative to the non-inferiority margin specified in the study. We then examined the concordance between the two-sided 95% CIs by the five statistical methods. The statistical methods were concordant if non-inferiority was shown based on CIs by all five methods. Similarly, the statistical methods were concordant if the conclusion was inconclusive based on CIs by all five methods. Concordance and agreement will be used interchangeably hereafter.

We chose not to compare the re-calculated CI to the study reported CI for the following reasons. First, most studies did not report the CI method used and some studies used stratified analysis, so we cannot validate or reproduce the study reported CI in most cases. Second, different studies used different confidence levels, where the comparison of different intervals is not meaningful.

### Statistical analysis

Descriptive analyses included number (percentage) for categorical variables and median (interquartile range IQR described as 25th and 75th percentile) for continuous variables.

We used graphs to describe the relationship of how CI width changed for different sample sizes and treatment success rates. For sample size, we used the smallest number of patients in an arm for a given study. For the treatment success rate, we used the average treatment success rate defined as the total number of successes divided by the total number of patients in both the treatment and control arms. The graph showed the relationship of CI width to sample size. To better illustrate the differences in CI width, the second graph shows the difference in CI width compared to the Wald method for the Agresti-Caffo, Newcombe, Miettinen-Nurminen, and SCAS methods in relation to sample size and stratified by success rates. On these graphs, a smooth line for each statistical method was fitted over the data points using a local polynomial regression. A subgroup analysis was done for sample size and treatment success scenarios with the largest difference between the smooth fitted lines. Additionally, a subgroup analysis was done for studies that did not randomize in a 1:1 ratio.

We assessed the agreement on conclusion by CIs using all five methods for non-inferiority margins that ranged from 0 to 20% by increment of 1%. As a sensitivity analysis, for studies with a sample size of greater than 200, we decreased the sample size to random sample sizes of 50, 100, 150, or 200 with the same success rate as reported by the study. Then we re-analyzed the agreement on conclusion by CIs based on the same range of non-inferiority margin.

All analyses were done with R version 3.6.3 (R Foundation for Statistical Computing, Vienna, Austria). CI for absolute risk reduction was calculated using the DescTools package [13]. CI was calculated based on the SCAS method using the ratesci package [14].

## Results

### Study characteristics

Of 227 antibiotic non-inferiority trials, 14 studies were excluded because they reported outcomes that were not proportions or did not report the raw numbers required to re-calculate the CI. Of the remaining 213 studies, nine studies compared two treatment arms to a comparison arm. One study compared three treatment arms to a comparison arm. Therefore, there were a total of 224 comparisons included in the analysis.

The study characteristics are described in Table 1. Of 224 comparisons, the statistical method used to calculate the CI was not reported in 134 (59.8%) cases. Comparison of studies that were published from 2001 to 2010 versus 2011 to 2019 is shown in Supplementary Materials 1 Table 1. The most commonly reported statistical method was the Wald method in 41 (18.3%) studies followed by the Miettinen-Nurminen method in 27 (12.1%) studies.

**Table 1:** Study characteristics

	Comparisons (*N*=224)
Smallest number of patients in either treatment or control group Median (IQR)	182 (101, 291)
Randomization in approximately 1:1 ratio	212 (94.6%)
Average treatment success rate in % Median (IQR)	84% (78%, 91%)
Statistical software used	
SAS	48 (21.4%)
Stata	19 (8.5%)
SPSS	16 (7.1%)
R	6 (2.7%)
Other	7 (3.1%)
Not specified	128 (57.1%)
Stratification used for primary outcome	51 (22.8%)
Description of the statistical method used to calculate the confidence interval for the primary outcome	
Wald^a^	41 (18.3%)
Miettinen-Nurminen	27 (12.1%)
Newcombe	12 (5.4%)
Agresti-Caffo	2 (0.9%)
Other methods^b^	8 (3.6%)
Not specified	134 (59.8%)
Study reported multiple CI methods	1 (0.5%)
Study reported two-sided 95% or one-sided 97.5% CI^c^	191 (85.3%)

*CI* confidence interval, *IQR* interquartile range

^a^Wald method includes studies that described using the normal approximation method.

^b^Other methods include bootstrap (*N*=1), exact method by Fagan (*N*=1), Farrington-Manning method (*N*=1), Gart-Nam method (*N*=1), generalized linear model (*N*=3), and exact method by Agresti-Min (*N*=1)

^c^Other confidence intervals include one-sided 90% (*N*=2), one-sided 95% (*N*=7), two-sided 90% (*N*=12), two-sided 97.5% (*N*=4). Eight studies did not report CIs

### Comparison of CIs by different statistical methods

The CIs calculated using the five statistical methods are described in Table 2. The CIs by different statistical methods and the CI reported by the study for each study are shown in Supplementary Materials 2. On average, the Miettinen-Nurminen method produced a wider CI than the other four methods. The Miettinen-Nurminen method produced the most conservative CI in 145 (64.7%) cases. The Wald method produced the most conservative CI in 15 (6.7%) cases. Sub-group analysis of studies that did not randomize in 1:1 ratio are shown in Supplementary Materials 1 Table 2.

**Table 2:** Confidence interval based on the five commonly used statistical methods

	CI width in % risk difference Median IQR	CI width difference compared to Wald method Median IQR^a^	Non-inferiority shown^b^ *N* (%)	Most conservative CI^c^
Wald	13.0 (10.8, 17.4)	Reference	170 / 216 (78.7%)	15 (6.7%)
Agresti-Caffo	13.3 (10.9, 18.5)	0.05 (0, 0.2)	169 / 216 (78.2%)	1 (0.5%)
SCAS	13.4 (11.1, 18.9)	0.2 (0.05, 0.4)	166 / 216 (76.9%)	53 (23.7%)
Newcombe	13.6 (11.1, 18.9)	0.1 (-0.01, 0.4)	165 / 216 (76.4%)	10 (4.5%)
Miettinen-Nurminen	13.6 (11.1, 19.0)	0.2 (0.04, 0.5)	165 / 216 (76.4%)	145 (64.7%)

*CI* confidence interval, *IQR* interquartile range, *SCAS* skewness-corrected asymptotic score method

^a^Difference calculated as CI width minus CI width by the Wald method, so a positive number suggests a CI that is wider than the CI by the Wald method

^b^There were 8 cases where non-inferiority margin was not specified

^c^Most conservative CI defined by the widest CI of the five methods for a given study

If non-inferiority was concluded based on the constructed two-sided 95% CI relative to the non-inferiority margins specified in the study, the CI by all five methods would be concordant in 211 of 216 (97.7%) studies that reported non-inferiority margins. In the five (2.3%) discordant cases, the conclusion was non-inferiority shown in all cases based on the Wald CIs and inconclusive in all cases based on the Newcombe and Miettinen-Nurminen CIs (Supplementary Materials 1 Table 3). CIs by the Wald method resulted in conclusions of non-inferiority in 170 (78.7%) cases, which was more than the other four methods. Non-inferiority was shown in the least number of cases using the CIs by the Newcombe and Miettinen-Nurminen methods.

**Table 3:** Confidence interval width by different statistical methods for the subgroup of 20 trials with a sample size of 100 or less in each arm and a success rate of 91% to 100%

	CI width in % risk difference Median IQR	CI width difference compared to Wald method Median IQR^a^	Non-inferiority shown^b^ *N* (%)	Most conservative CI^c^
Wald	14.5 (11.7, 17.0)	Reference	17/18 (94%)	0 (0%)
Agresti-Caffo	16.9 (13.2, 20.1)	1.4 (1.0, 3.2)	16/18 (89%)	0 (0%)
SCAS	17.4 (13.5, 20.8)	2.1 (1.4, 3.8)	15/18 (83%)	0 (0%)
Newcombe	18.1 (13.9, 23.4)	2.5 (1.7, 5.3)	14/18 (78%)	3 (15.0%)
Miettinen-Nurminen	17.8 (14.0, 23.8)	2.7 (1.8, 5.1)	14/18 (78%)	17 (85.0%)

*CI* confidence interval, *IQR* interquartile range, *SCAS* skewness-corrected asymptotic score method

^a^Difference calculated as CI width minus CI width by the Wald method, so a positive number suggests a CI that is wider than the CI by the Wald method

^b^There were two cases where non-inferiority margin was not specified

^c^Most conservative CI defined by the widest CI of the five methods for a given study

### Relationship of CI width to sample size and success rate

**Fig. 1:**
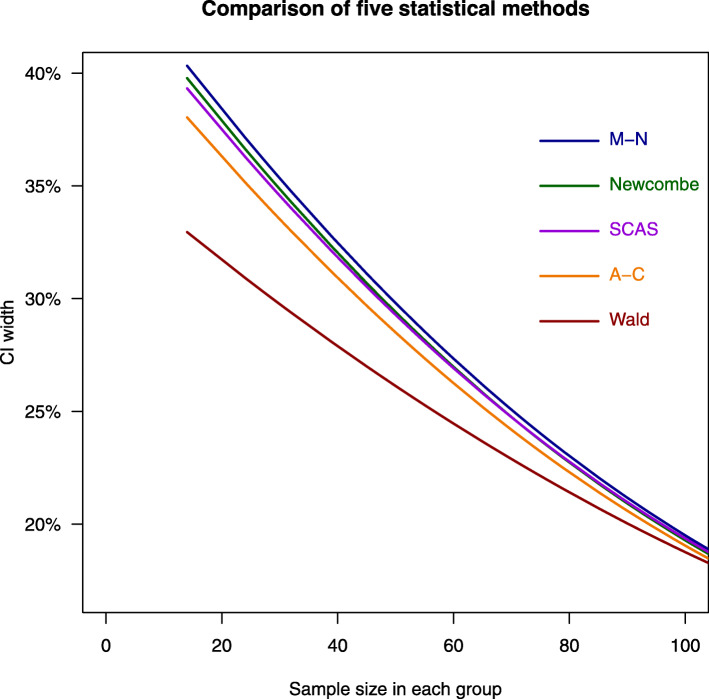
Relationship of confidence interval width to sample size. A-C, Agresti-Caffo method; CI, confidence interval; M-N, Miettinen-Nurminen method; SCAS, skewness-corrected asymptotic score method. Each line is a smooth fitted line of data points for each statistical method. After sample size in each group increases to more than 100, the 5 methods converge to even smaller differences that are not shown in this figure

With increasing sample size, the differences in CI width between statistical methods became smaller (Fig. 1). The CI width by different methods to sample size and stratified by success rate is shown in Supplementary Materials 1 Figure 1.

**Fig. 2:**
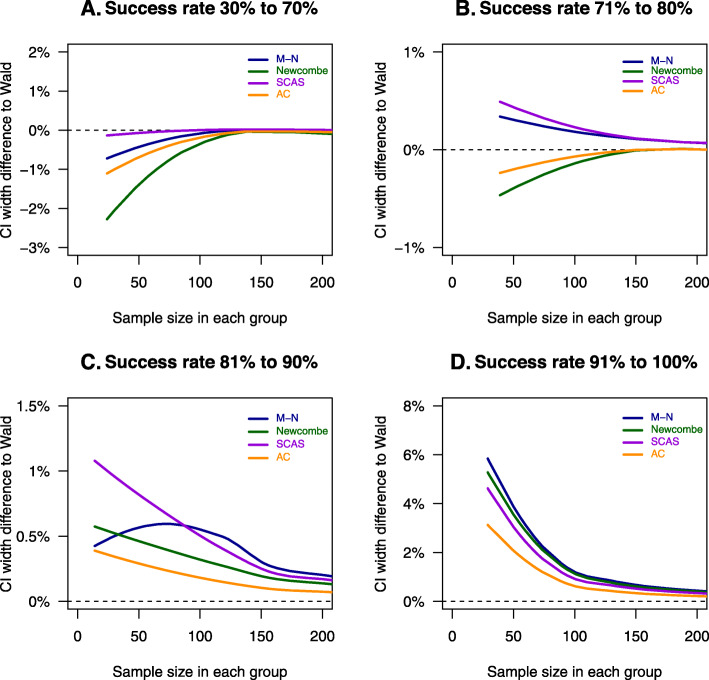
Differences in confidence interval compared to Wald method in relation to sample size and stratified by success rate. A-C, Agresti-Caffo method; CI, confidence interval; M-N, Miettinen-Nurminen method; SCAS, skewness-corrected asymptotic score method. The CI width difference is the method of interest minus the Wald CI. Each line is a smooth fitted line of data points for each statistical method. Note that the *y*-axis scale is different

The smooth fitted line of difference in CI width compared to Wald method by the four other statistical methods to sample size stratified by different treatment success rates are shown in Fig. 2. The CI width relative to other methods varies depending on the average underlying success rates. For example, when the success rate ranged from 30 to 70%, the Wald method produced the widest CI. When the success rate was 81% or higher, the Wald method produced the narrowest CI.

Based on Figs. 1 and 2, the CI width differed greatly between the statistical methods in trials with a success rate of 91% to 100% and sample size of 100 or less in each arm. Therefore, we performed a post hoc sub-group analysis of trials with a success rate of 91% to 100% and a sample size of 100 or less in each arm.

The CI width by different statistical methods for the subgroup of the 20 trials with a success rate of 91% to 100% and sample size of 100 or less in each arm are described in Table 3. For these 20 trials, CIs by the Wald method were much narrower than the other three methods. The conclusion would be non-inferiority shown based on the Wald CIs but inconclusive based on the Newcombe or Miettinen-Nurminen CIs in three cases.

### Agreement of conclusion on non-inferiority with varying non-inferiority margin

**Fig. 3:**
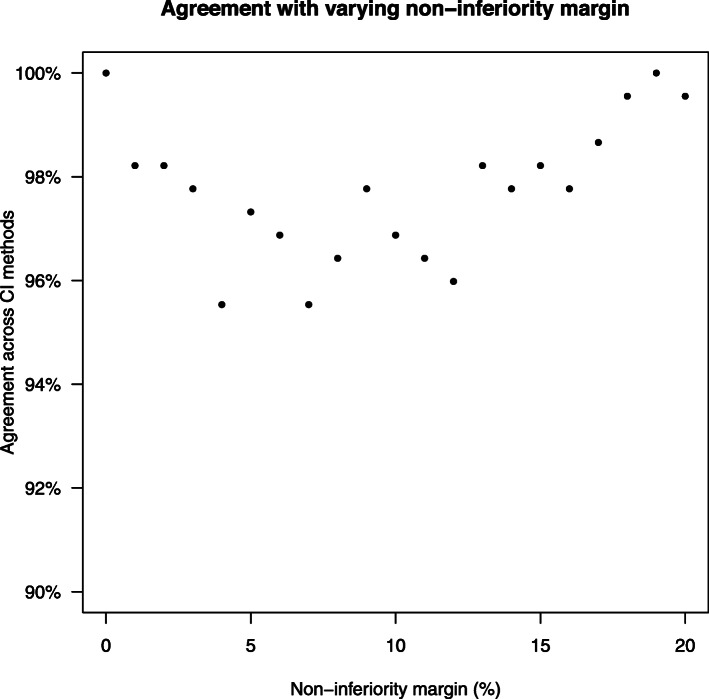
Agreement across confidence interval methods for different non-inferiority margins. CI, confidence interval

**Fig. 4:**
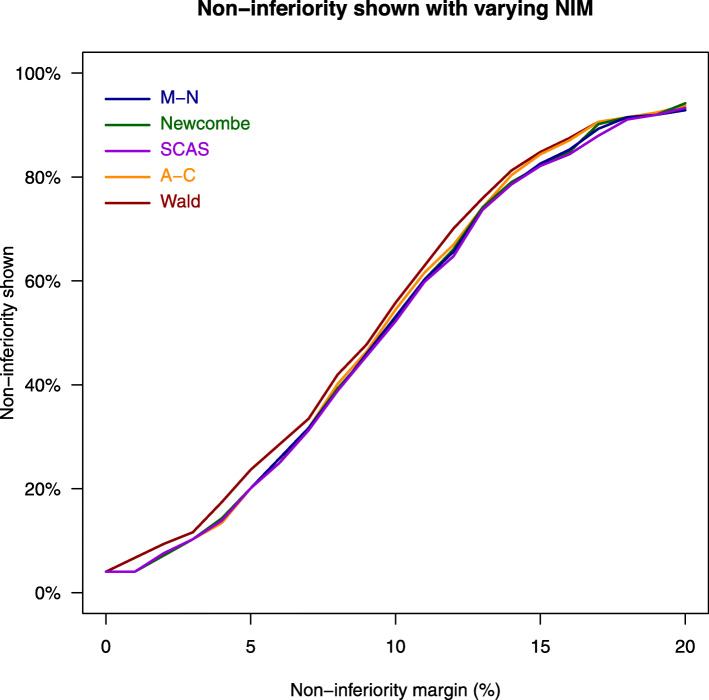
Percentage of non-inferiority shown by different confidence interval methods for different non-inferiority margins. A-C, Agresti-Caffo method; CI, confidence interval; M-N, Miettinen-Nurminen method; NIM, non-inferiority margin; SCAS, skewness-corrected asymptotic score method

Figure 3 shows the agreement between all five statistical methods in terms of conclusion of non-inferiority with varying non-inferiority margin from 0 to 20%. The agreement ranged from 95.5 to 100%. The proportion of cases where non-inferiority is shown using each method for varying non-inferiority margin is shown in Fig. 4. Sub-sampling of larger trials with a sample size of greater than 200 showed similar results (Supplementary Materials 1 Figure 2 and 3).

## Discussion

In a secondary analysis of a systematic review that included 213 antibiotic non-inferiority trials, we compared the CIs based on the five most common statistical methods. The CIs based on these statistical methods may reach a different conclusion on non-inferiority for the same study in approximately 2% of cases. The change in study conclusion does not reflect the change in CI width entirely. Significant differences in CI width may not change the study conclusion if the point estimate is very distant from the non-inferiority margin. The difference in CI width by these statistical methods ranged from 0.05 to 0.2% on average, which is not negligible. There can be considerable differences for an individual study. The differences in CI width by statistical methods were very apparent in trials with a small sample size of 100 or less in each arm and a high treatment success rate of above 90%. On average, the CI width by the Miettinen-Nurminen method was wider than the Wald method by 2.7% in absolute risk difference. To put this into perspective, a difference of 2.7% would be more than a quarter of the commonly used non-inferiority margin of 10%. The Wald method was the most commonly used method, but it produced narrower CIs, and would lead to a conclusion of non-inferiority more often (and incorrectly so, according to prior studies on coverage probabilities [3, 5]). In contrast, the Miettinen-Nurminen method produced wider CIs and would lead to a conclusion of non-inferiority less frequently.

In a similar study that included 11 HIV non-inferiority trials, the Wald method, an exact method, the Newcombe method, and the Farrington-Manning method were compared [15]. The Farrington-Manning method is closely related to the Miettinen-Nurminen method and both methods produce almost identical CIs. In general, the Wald method produced narrower CIs while the Farrington-Manning method produced wider CIs [15]. The CIs by the five different statistical methods reached a different conclusion on non-inferiority in two of the 11 trials [15]. These findings are similar to the findings from our much larger study. The pattern of larger differences between CIs by different methods in smaller sample sizes and success rates further away from 50% found in our study is consistent with a prior study on coverage probabilities [3]. Our study complements these findings as our study focuses on parameters of real trials rather than the theoretical wide ranging and arbitrarily stipulated parameters.

The strength of our study is in the systematic and comprehensive literature search that yielded the largest number of non-inferiority trials to date for the description and comparison of statistical methods for CI of risk differences.

There are several limitations to our study that merit mentioning. First, we could not reproduce or verify the stratified methods for calculating the CI in the trials, because we could not access the trials’ patient-level data. However, most studies (78%) constructed the CI without any stratification. Second, the statistical methods for estimating the CI in this study were far from exhaustive. We selected the five statistical methods that were most commonly used and/or performed well in terms of coverage probabilities [3]. In particular, we did not include an exact method. Some statisticians suggest that exact methods may be worse than approximate methods for estimating the CI of binomial proportions [16]. The specific scenarios such as small sample sizes less than 30 (in both arms altogether) where the exact method may be better [3] is not applicable to most non-inferiority RCTs. Third, our comparison of statistical methods is based on the observed treatment effect, which is prone to bias and may be different from the true effect.

Our study should be interpreted with caution. The objective of our study was to describe the differences in CI between statistical methods in real-life circumstances in terms of antibiotic non-inferiority trials. These differences in CI width should not be used to guide decisions on what CI method to use. Researchers should always use the method that presents the most accurate confidence interval based on coverage probabilities as reported in prior studies [3, 5]. This should be the case even if there are small differences between methods such as for larger samples where the differences will not change the study conclusion. Nevertheless, our study does illustrate whether the prior antibiotic non-inferiority trials that used the Wald method would have obtained a different conclusion if a more accurate method had been employed. It is reassuring that the conclusion on non-inferiority stayed consistent when another method was used in most cases.

Our study findings indicate room for improvement in the conduct and reporting of future non-inferiority RCTs. First, the use of the Wald method for estimating the CI should be discouraged. In our study, the Wald method was the most common method used in trials. Yet, the coverage probability of CI by the Wald method has previously been shown to be too liberal [3]. The Wald method may be popular for being simple and intuitive, but it is based on a flawed assumption that an expected population proportion is normally distributed about an observed proportion [5]. There is no excuse to prefer the Wald method, especially since the most commonly used statistical software (e.g. SAS, R, and Stata) used in the trials within our study can calculate the CI using methods other than the Wald method [3, 7]. Second, the statistical method used to calculate the CI should be described clearly in the journal publication. In our study, the statistical method for CI was not described in approximately 60% of trials. Since the study conclusion of non-inferiority is based solely on the CI in most trials and CIs by different statistical methods may reach different conclusions in 2% cases, it is important to describe the statistical method for reproducibility. One way to improve on reporting is to add CI statistical method as a criterion for FDA industry guidance documents and reporting guidelines for non-inferiority trials.

## Conclusions

Different statistical methods for CI may result in different conclusions on non-inferiority in 2.3% cases. Yet, the statistical method used to calculate CI was not reported in the majority of antibiotic non-inferiority trials. The differences in CI width by different methods can be significant for an individual study. In trials with a sample size of 100 or less in each group and a treatment success rate above 90%, the Wald method resulted in CIs that were much narrower than other methods. Reporting guidelines and industry guidance documents should mandate reporting of the statistical methods and discourage use of the Wald method.

## Supplementary Information

**Additional file 1:.** Supplementary Material 1

**Additional file 2:.** Supplementary Material 2

**Additional file 3:.** Supplementary Material 3

## Data Availability

All data generated or analyzed during this study are included in Supplementary Materials 3.

## Abbreviations

CI: Confidence interval
FDA: Food and Drug Administration
IQR: Interquartile range
RCT: Randomized controlled trial
SCAS: Skewness-corrected asymptotic score method
