# Supplementary material for: Confidence interval of risk difference by different statistical methods and its impact on the study conclusion in antibiotic non-inferiority trials
Source: Trials. 2021 Oct 16;22:708. doi: 10.1186/s13063-021-05686-8 (PMC8520289; doi:10.1186/s13063-021-05686-8)

Supplementary Materials 1. Tables and Figures

Supplementary Table 1. Comparison of studies by publication year

|  | Publication year 2001 to 2010  (N= 80) | Publication year  2011 to 2019  (N= 144) | P-value |
| --- | --- | --- | --- |
| CI method not specified | 51 (63.8%) | 83 (57.6%) | 0.3963 |
| Pharmaceutical industry funding | 66 (82.5%) | 70 (48.6%) | <0.0001 |
| Smallest number of patients in either treatment or control group  Median (IQR) | 178 (120.5, 260.8) | 184.5 (92.3, 307.8) | 0.8490 |
| Average treatment success rate in %  Median (IQR) | 85% (79%, 91%) | 84% (78%, 91%) | 0.7772 |

CI = confidence interval, IQR = interquartile range

Comparisons between two groups were done with Wilcoxon rank-sum test for continuous variables and Fisher’s exact test for proportions.

Supplementary Table 2. Sub-group analysis of 12 trials that randomized in ratios other than 1:1

|  | CI width in % risk difference  Median IQR | CI width difference compared to Wald method  Median IQR^a^ | Non-inferiority shown  N (%) |
| --- | --- | --- | --- |
| Wald | 12.5  (11.2, 15.3) | Reference | 10 (83%) |
| Agresti-Caffo | 12.8  (11.2, 15.5) | 0.2  (0.05, 0.3) | 10 (83%) |
| SCAS | 12.9  (11.3, 15.6) | 0.2  (0.09, 0.4) | 10 (83%) |
| Newcombe | 13.0  (11.3, 15.6) | 0.2  (0.07, 0.4) | 10 (83%) |
| Miettinen-Nurminen | 13.0  (11.4, 15.7) | 0.2  (0.09, 0.5) | 10 (83%) |

CI = confidence interval; IQR = interquartile range; SCAS = skewness-corrected asymptotic score

^a^Difference calculated as CI width minus CI width by Wald method, so a positive number suggests a CI that is wider than the CI by Wald method

Supplementary Table 3. Cases in which statistical methods disagree on non-inferiority

|  | F130  Molina  2013 | F150  Darouiche  2014 | F202  D’Ignazio  2005 | F58  Tazuma  2015 | F81  Park  2014 |
| --- | --- | --- | --- | --- | --- |
| Smallest N | 170 | 27 | 174 | 58 | 29 |
| Randomized in 1:1 ratio | Yes | Yes | Yes | Yes | Yes |
| Total success rate | 91% | 100% | 92% | 93% | 93% |
| Wald | NI shown | NI shown | NI shown | NI shown | NI shown |
| Agresti-Caffo | NI shown | NI shown | NI shown | Inconclusive | NI shown |
| Newcombe | Inconclusive | Inconclusive | Inconclusive | Inconclusive | Inconclusive |
| SCAS | Inconclusive | NI shown | Inconclusive | Inconclusive | Inconclusive |
| Miettinen-Nurminen | Inconclusive | Inconclusive | Inconclusive | Inconclusive | Inconclusive |

NI = non-inferiority; SCAS = skewness-corrected asymptotic score

Supplementary Figure 1. CI width by success rate and sample size


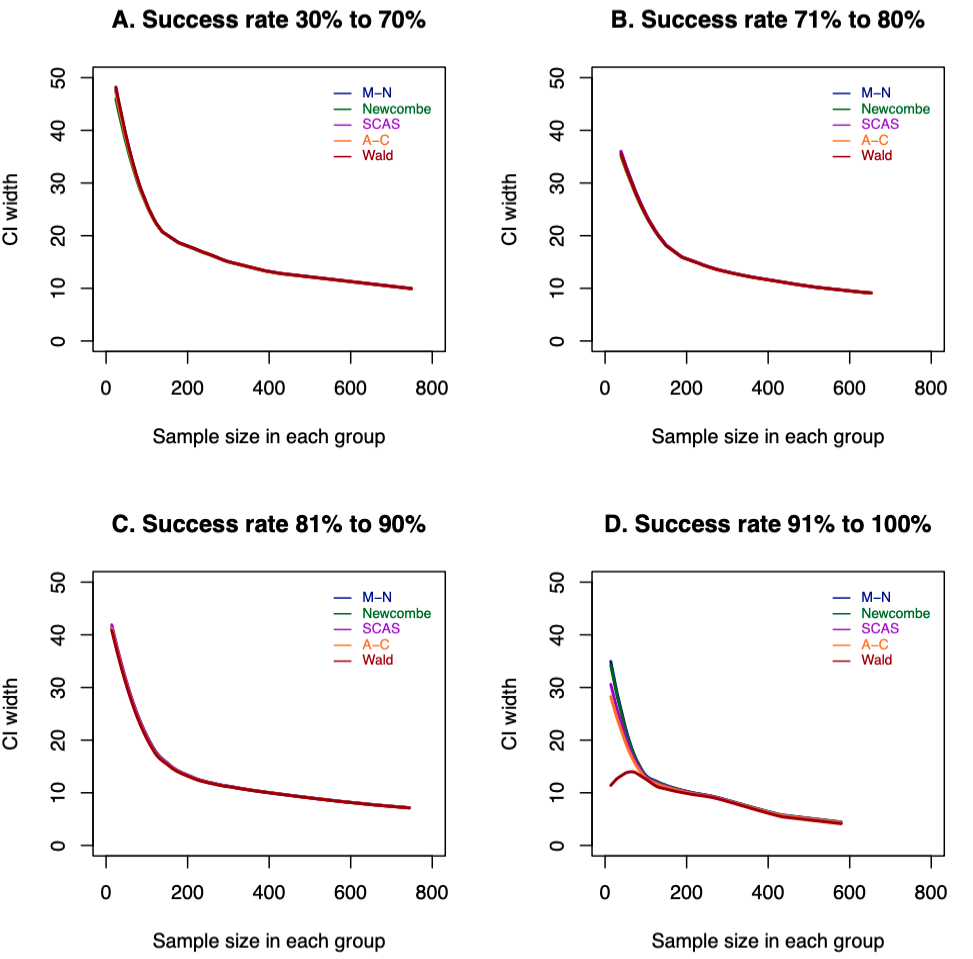


Supplementary Figure 2. Agreement across CI methods with varying non-inferiority margin


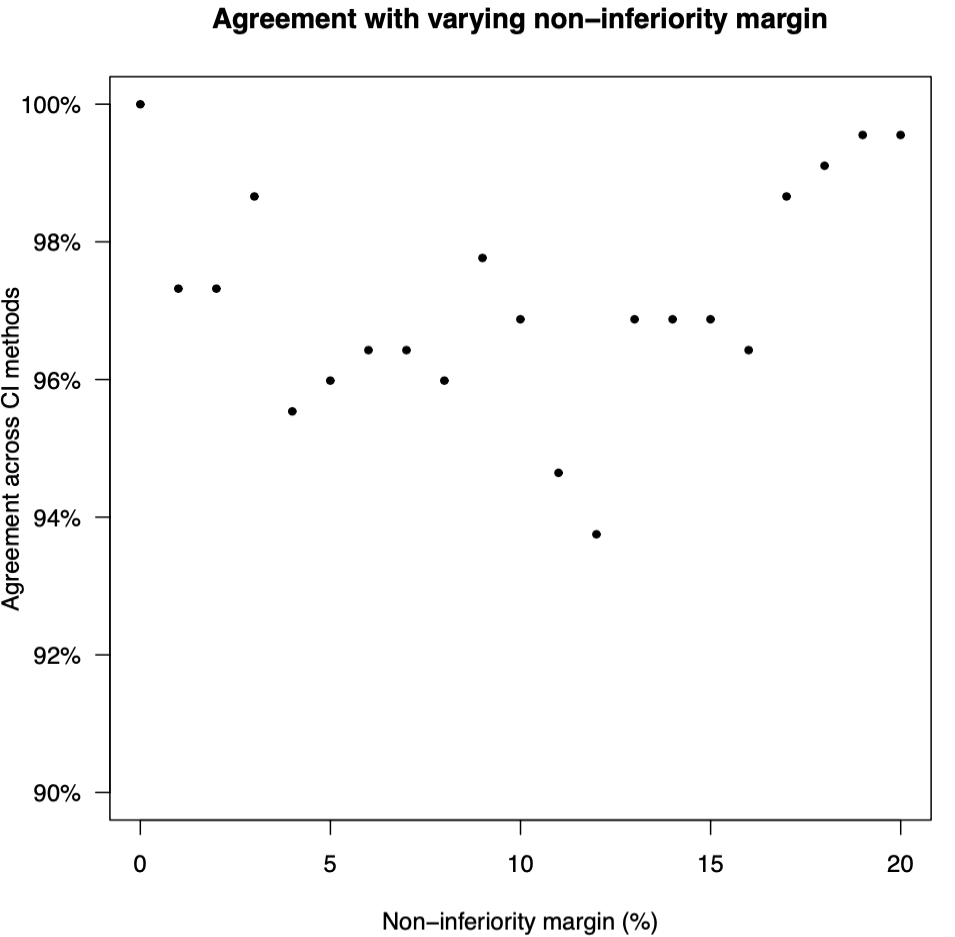


Supplementary Figure 3. Non-inferiority shown by different CI methods for varying non-inferiority margins


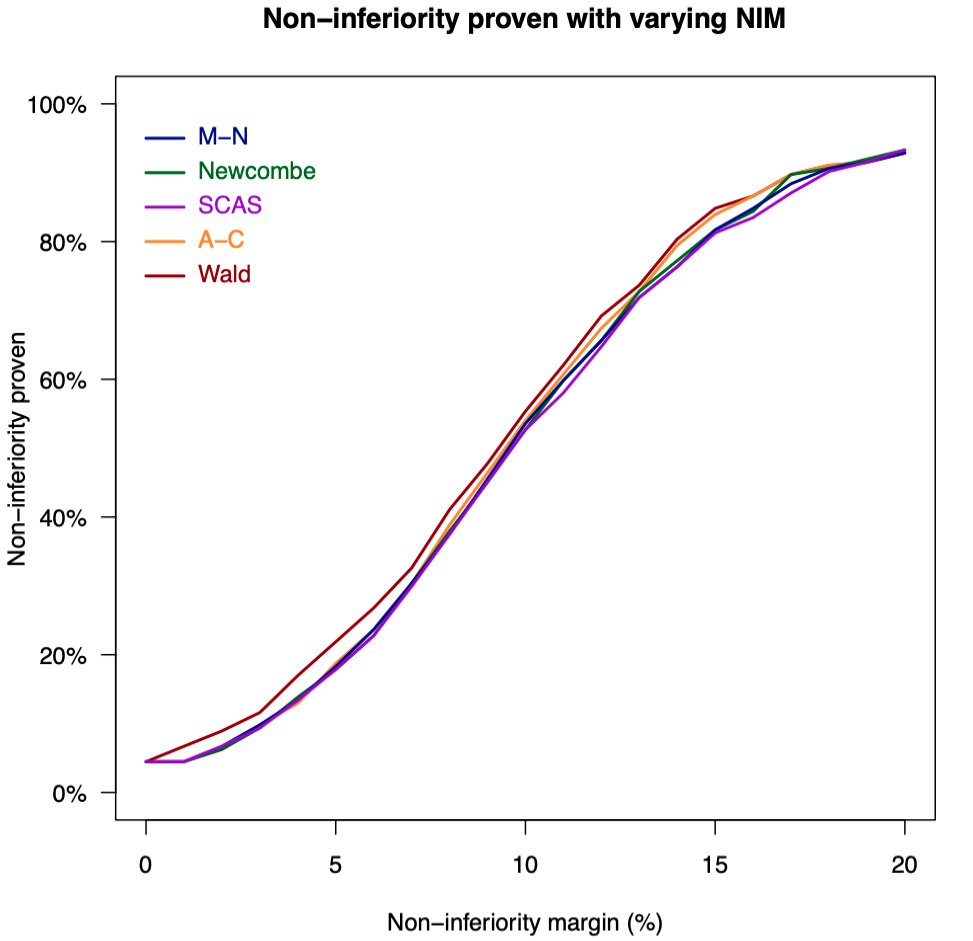

Supplement: Supplementary file 1 — Additional file 1:. Supplementary Material 1 [file 13063_2021_5686_MOESM1_ESM.docx]
