# Supplementary material for: Confidence interval of risk difference by different statistical methods and its impact on the study conclusion in antibiotic non-inferiority trials
Source: Trials. 2021 Oct 16;22:708. doi: 10.1186/s13063-021-05686-8 (PMC8520289; doi:10.1186/s13063-021-05686-8)
Supplement: Supplementary file 2 — Additional file 2:. Supplementary Material 2 [file 13063_2021_5686_MOESM2_ESM.pdf]

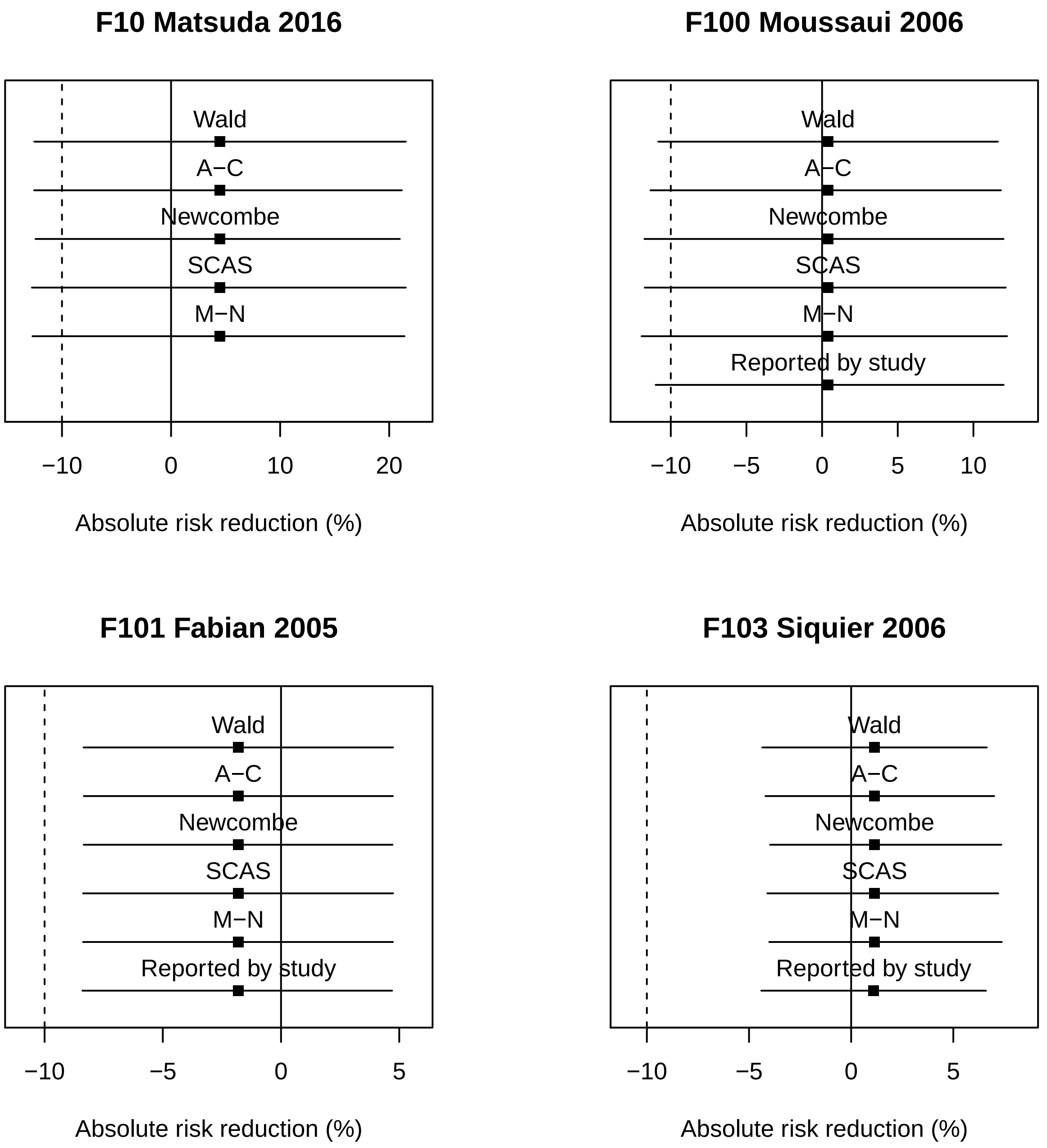

F10 Matsuda 2016
Wald
A−C
Newcombe
SCAS
M−N
−10
0
10
20
Absolute risk reduction (%)
F100 Moussaui 2006
Wald
A−C
Newcombe
SCAS
M−N
Reported by study
−10
−5
0
5
10
Absolute risk reduction (%)
F101 Fabian 2005
Wald
A−C
Newcombe
SCAS
M−N
Reported by study
−10
−5
0
5
Absolute risk reduction (%)
F103 Siquier 2006
Wald
A−C
Newcombe
SCAS
M−N
Reported by study
−10
−5
0
5
Absolute risk reduction (%)

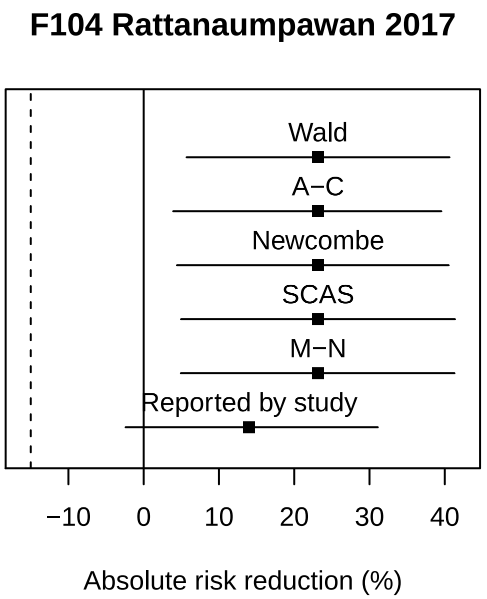
F104 Rattanaumpawan 2017
Wald
A−C
Newcombe
SCAS
M−N
Reported by study
−10
0
10
20
30
40
Absolute risk reduction (%)

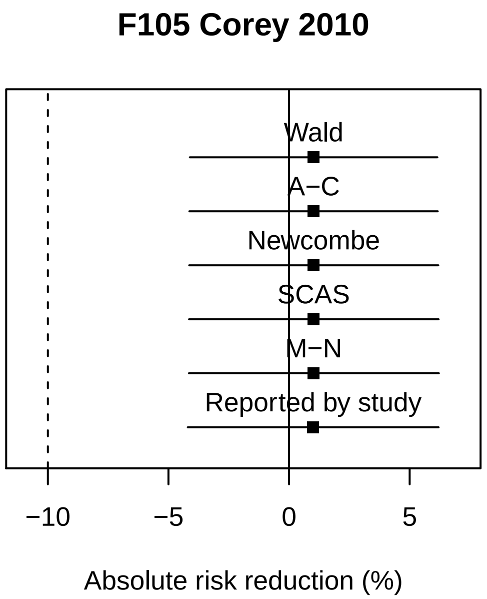
F105 Corey 2010
Wald
A−C
Newcombe
SCAS
M−N
Reported by study
−10
−5
0
5
Absolute risk reduction (%)

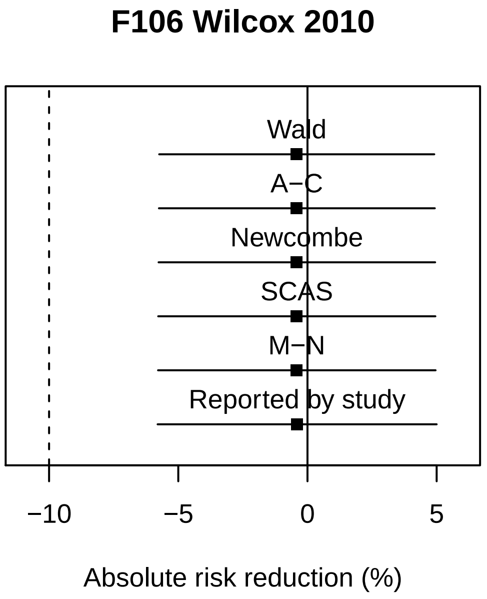
F106 Wilcox 2010
Wald
A−C
Newcombe
SCAS
M−N
Reported by study
−10
−5
0
5
Absolute risk reduction (%)

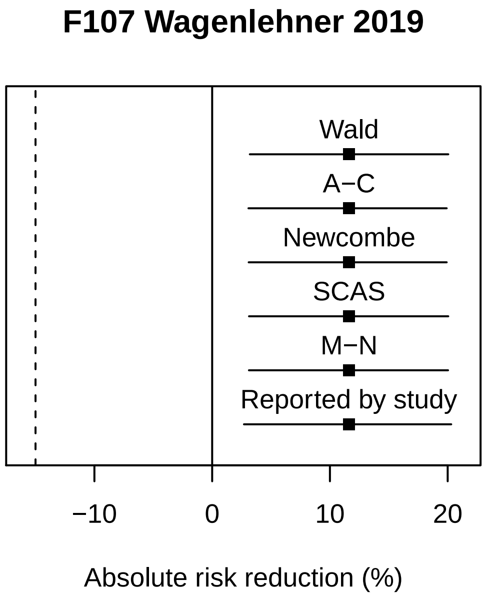
F107 Wagenlehner 2019
Wald
A−C
Newcombe
SCAS
M−N
Reported by study
−10
0
10
20
Absolute risk reduction (%)

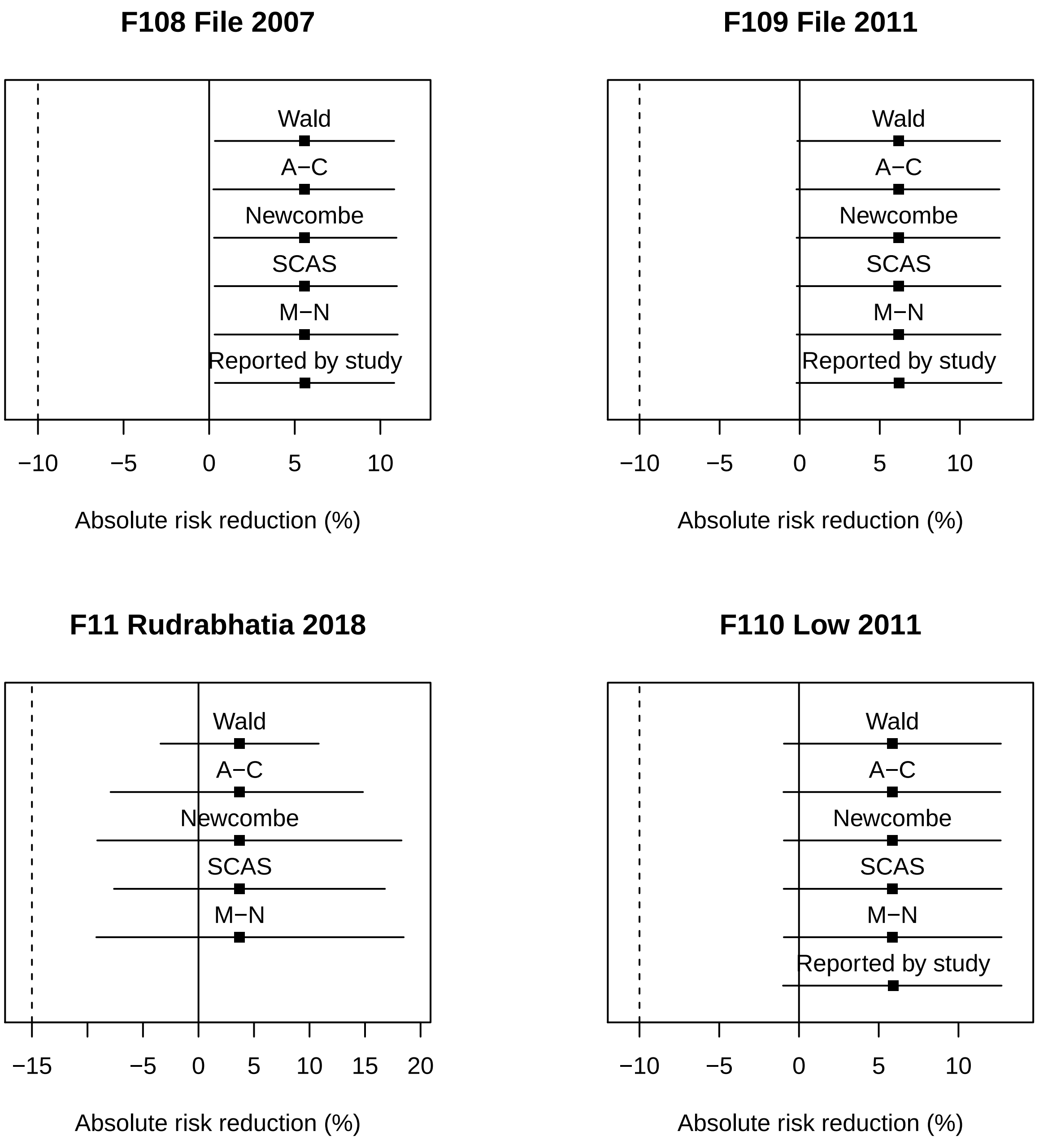
F108 File 2007
Wald
A−C
Newcombe
SCAS
M−N
Reported by study
−10
−5
0
5
10
Absolute risk reduction (%)
F109 File 2011
Wald
A−C
Newcombe
SCAS
M−N
Reported by study
−10
−5
0
5
10
Absolute risk reduction (%)
F11 Rudrabhatia 2018
Wald
A−C
Newcombe
SCAS
M−N
−15
−5
0
5
10
15
20
Absolute risk reduction (%)
F110 Low 2011
Wald
A−C
Newcombe
SCAS
M−N
Reported by study
−10
−5
0
5
10
Absolute risk reduction (%)

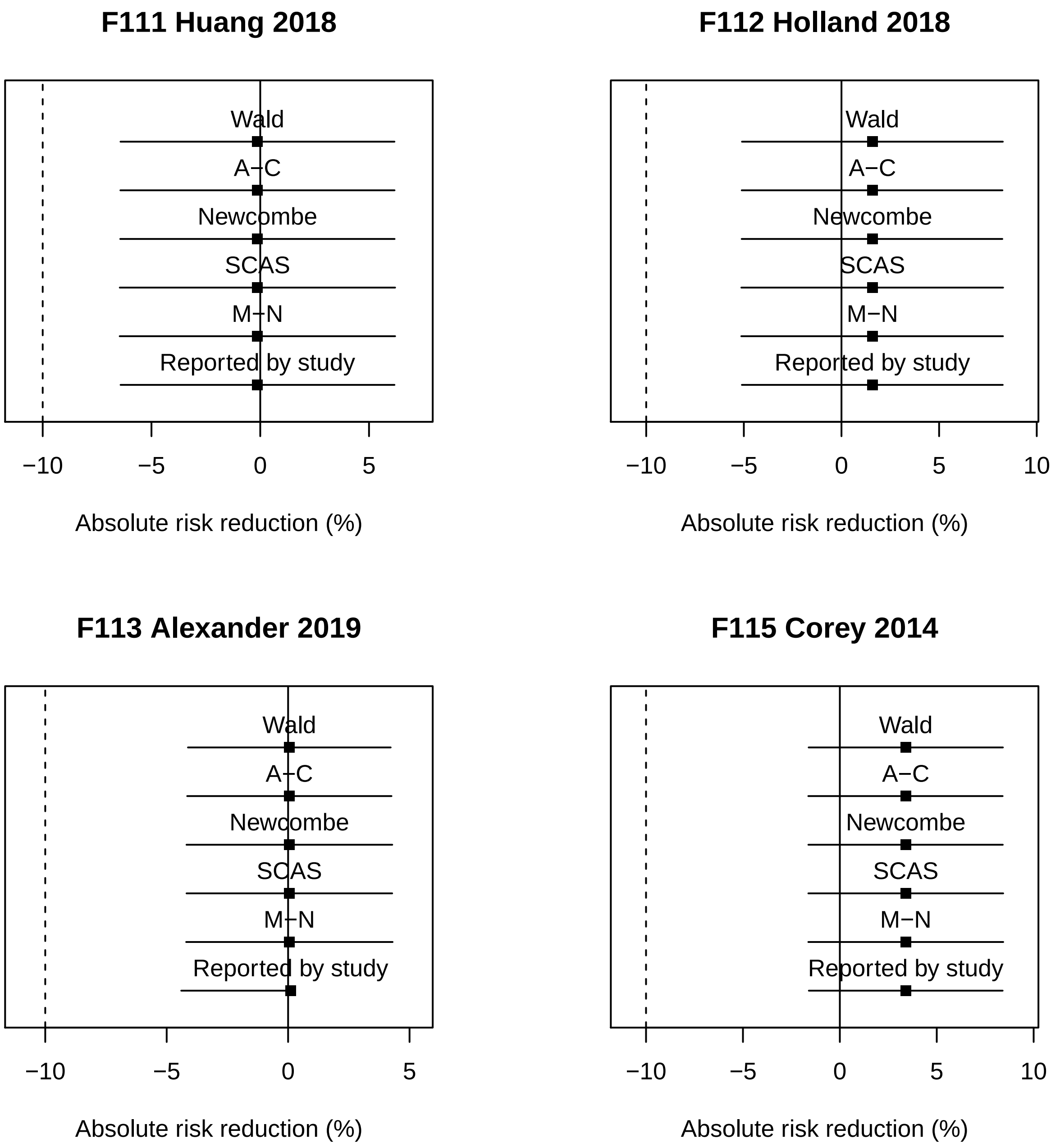
F111 Huang 2018
Wald
A−C
Newcombe
SCAS
M−N
Reported by study
−10
−5
0
5
Absolute risk reduction (%)
F112 Holland 2018
Wald
A−C
Newcombe
SCAS
M−N
Reported by study
−10
−5
0
5
10
Absolute risk reduction (%)
F113 Alexander 2019
Wald
A−C
Newcombe
SCAS
M−N
Reported by study
−10
−5
0
5
Absolute risk reduction (%)
F115 Corey 2014
Wald
A−C
Newcombe
SCAS
M−N
Reported by study
−10
−5
0
5
10
Absolute risk reduction (%)

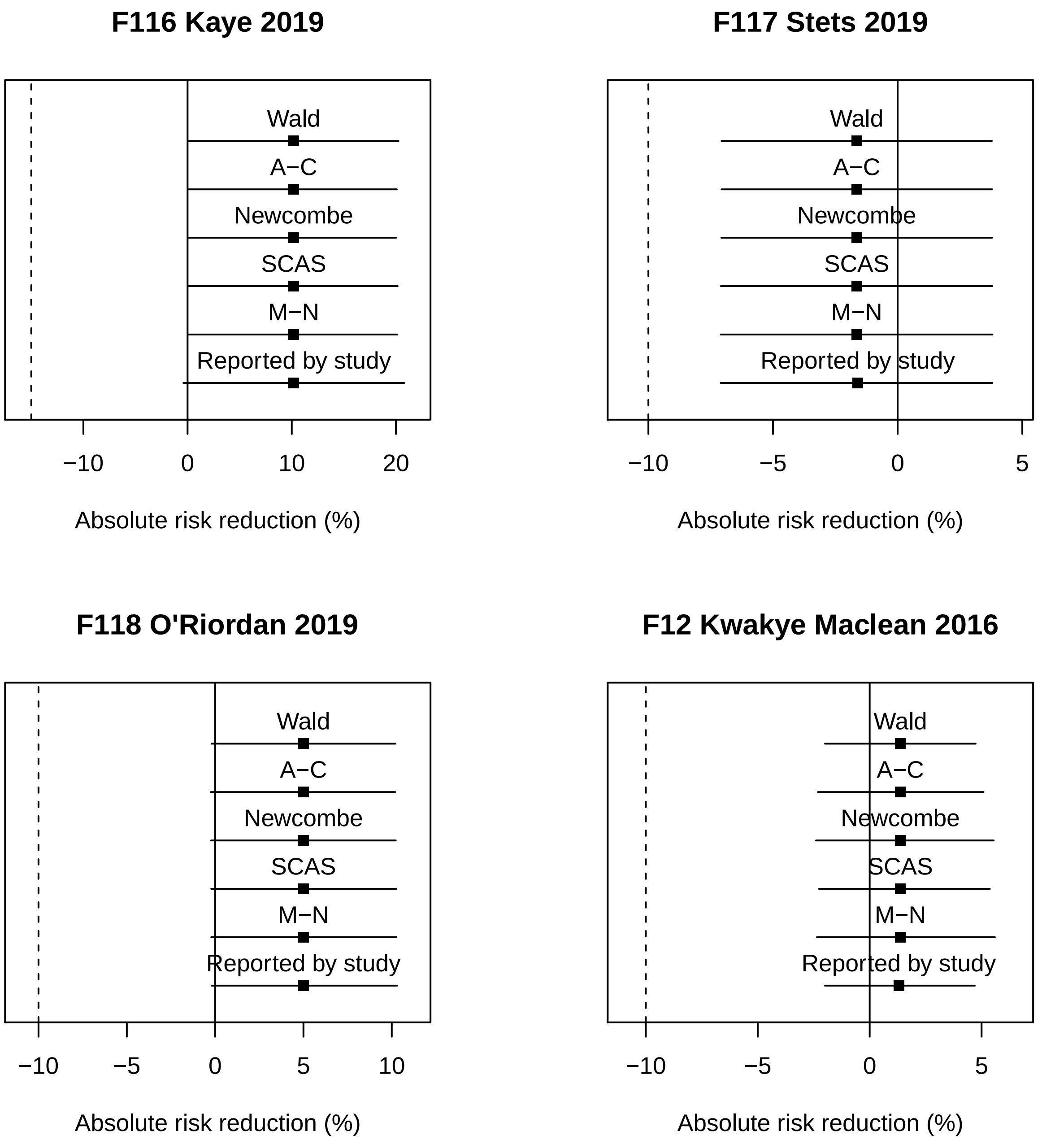

F116 Kaye 2019
Wald
A−C
Newcombe
SCAS
M−N
Reported by study
−10
0
10
20
Absolute risk reduction (%)
F117 Stets 2019
Wald
A−C
Newcombe
SCAS
M−N
Reported by study
−10
−5
0
5
Absolute risk reduction (%)
F118 O'Riordan 2019
Wald
A−C
Newcombe
SCAS
M−N
Reported by study
−10
−5
0
5
10
Absolute risk reduction (%)
F12 Kwakye Maclean 2016
Wald
A−C
Newcombe
SCAS
M−N
Reported by study
−10
−5
0
5
Absolute risk reduction (%)

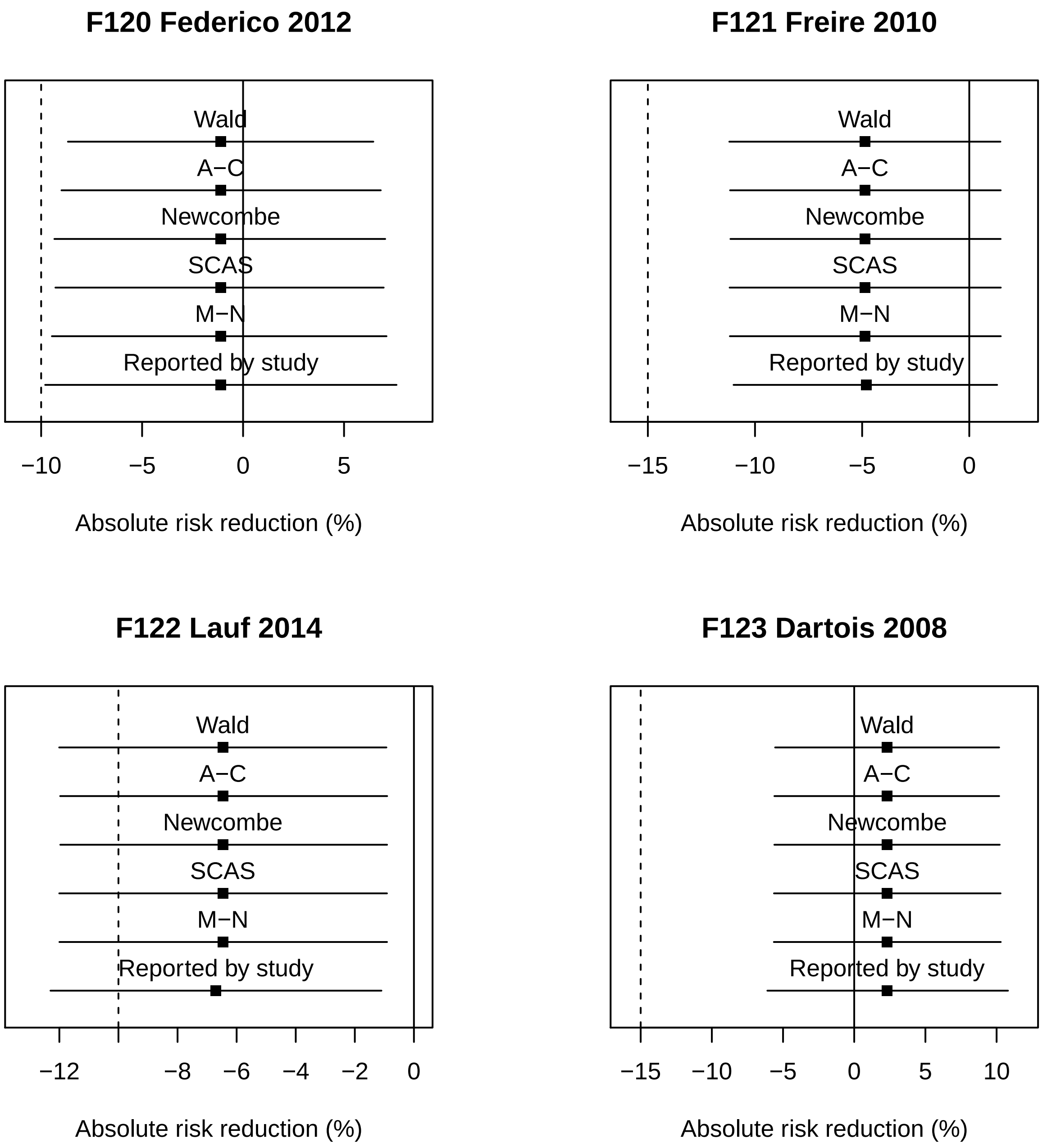

F120 Federico 2012
Wald
A−C
Newcombe
SCAS
M−N
Reported by study
−10
−5
0
5
Absolute risk reduction (%)
F121 Freire 2010
Wald
A−C
Newcombe
SCAS
M−N
Reported by study
−15
−10
−5
0
Absolute risk reduction (%)
F122 Lauf 2014
Wald
A−C
Newcombe
SCAS
M−N
Reported by study
−12
−8
−6
−4
−2
0
Absolute risk reduction (%)
F123 Dartois 2008
Wald
A−C
Newcombe
SCAS
M−N
Reported by study
−15
−10
−5
0
5
10
Absolute risk reduction (%)

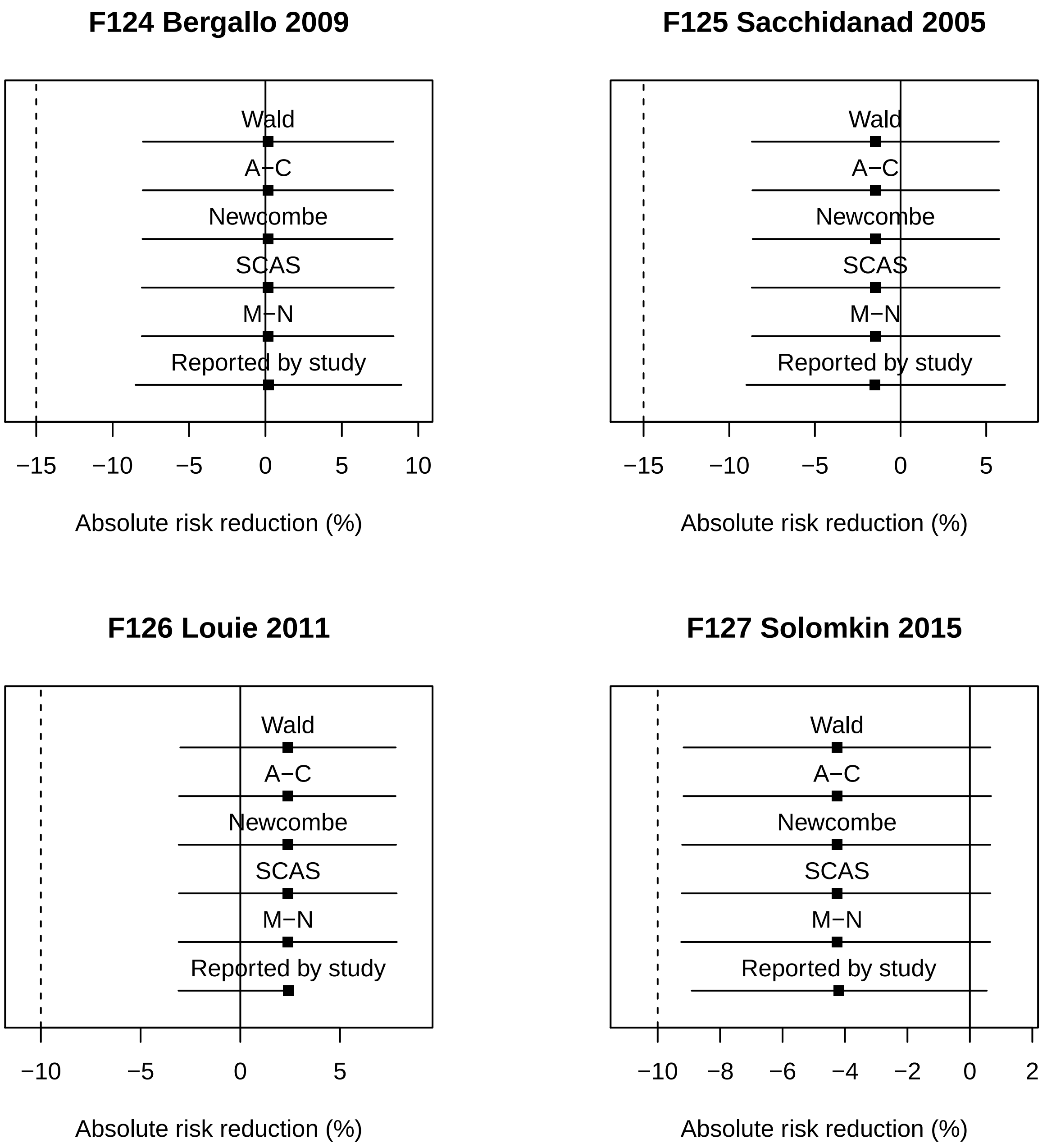
F124 Bergallo 2009
Wald
A−C
Newcombe
SCAS
M−N
Reported by study
−15
−10
−5
0
5
10
Absolute risk reduction (%)
F125 Sacchidanad 2005
Wald
A−C
Newcombe
SCAS
M−N
Reported by study
−15
−10
−5
0
5
Absolute risk reduction (%)
F126 Louie 2011
Wald
A−C
Newcombe
SCAS
M−N
Reported by study
−10
−5
0
5
Absolute risk reduction (%)
F127 Solomkin 2015
Wald
A−C
Newcombe
SCAS
M−N
Reported by study
−10
−8
−6
−4
−2
0
2
Absolute risk reduction (%)

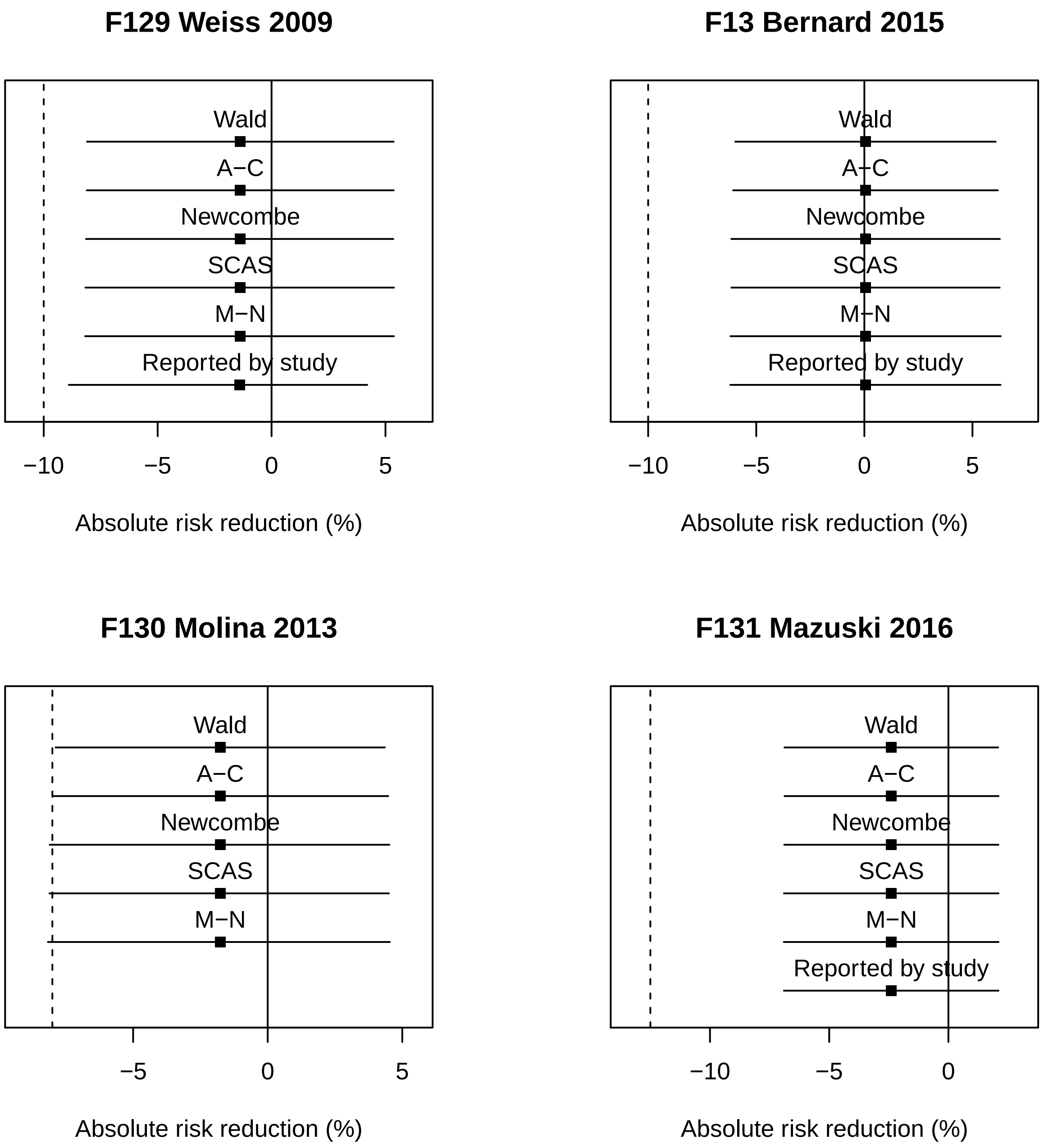
F129 Weiss 2009
Wald
A−C
Newcombe
SCAS
M−N
Reported by study
−10
−5
0
5
Absolute risk reduction (%)
F13 Bernard 2015
Wald
A−C
Newcombe
SCAS
M−N
Reported by study
−10
−5
0
5
Absolute risk reduction (%)
F130 Molina 2013
Wald
A−C
Newcombe
SCAS
M−N
−5
0
5
Absolute risk reduction (%)
F131 Mazuski 2016
Wald
A−C
Newcombe
SCAS
M−N
Reported by study
−10
−5
0
Absolute risk reduction (%)

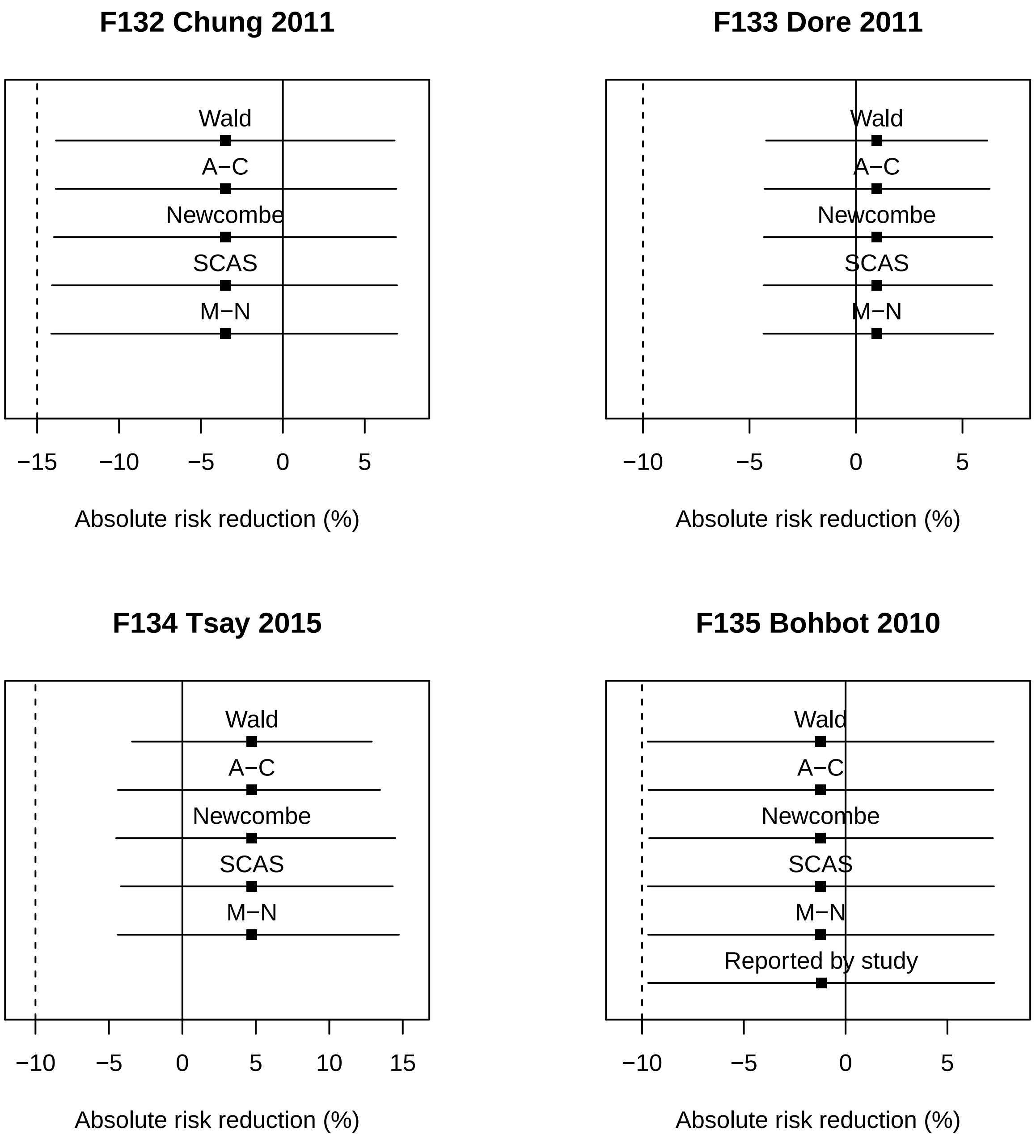
F132 Chung 2011
Wald
A−C
Newcombe
SCAS
M−N
−15 −10 −5 0 5
Absolute risk reduction (%)
F133 Dore 2011
Wald
A−C
Newcombe
SCAS
M−N
−10 −5 0 5
Absolute risk reduction (%)
F134 Tsay 2015
Wald
A−C
Newcombe
SCAS
M−N
−10 −5 0 5 10 15
Absolute risk reduction (%)
F135 Bohbot 2010
Wald
A−C
Newcombe
SCAS
M−N
Reported by study
−10 −5 0 5
Absolute risk reduction (%)

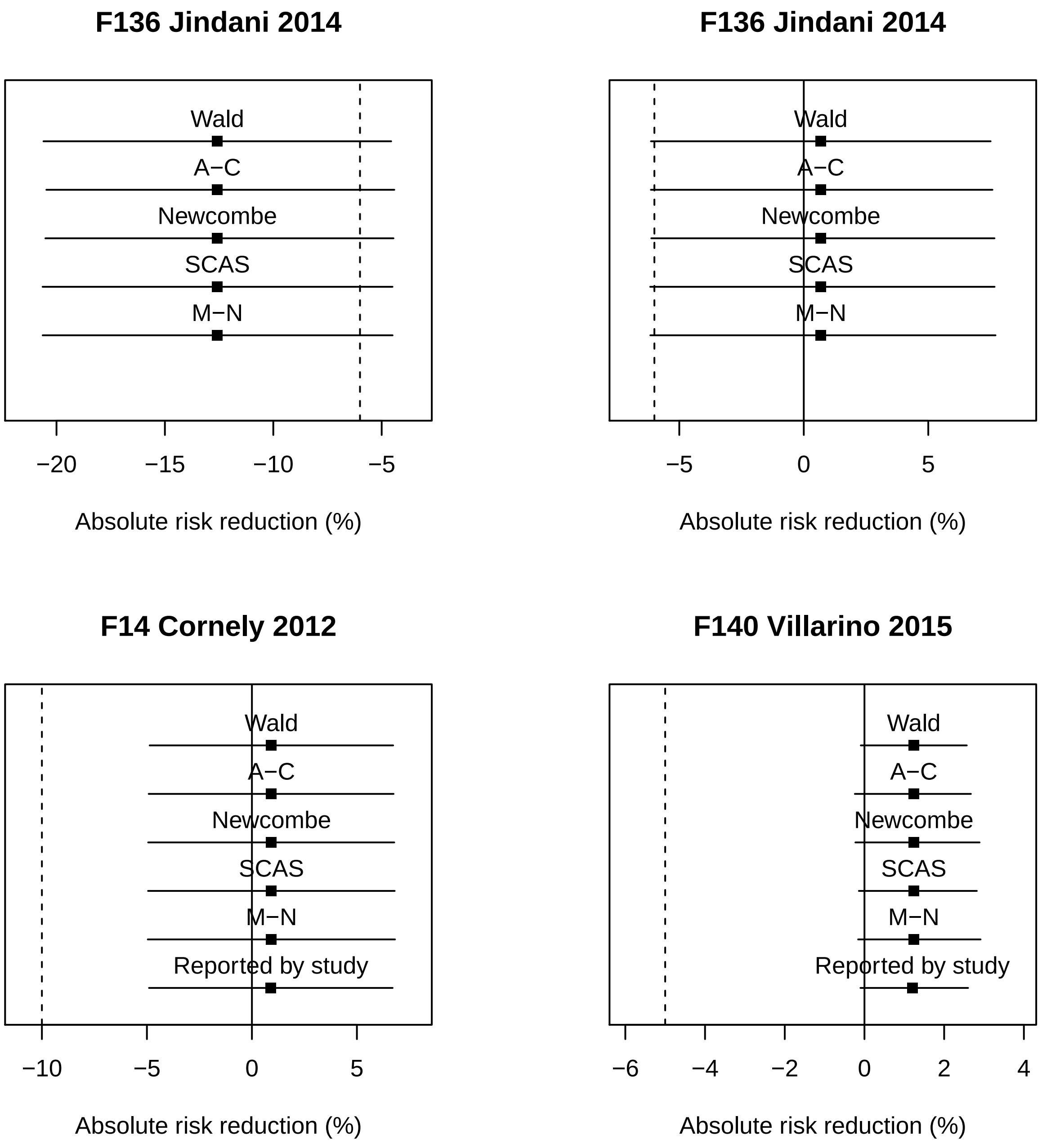
F136 Jindani 2014
Wald
A−C
Newcombe
SCAS
M−N
−20
−15
−10
−5
Absolute risk reduction (%)
F136 Jindani 2014
Wald
A−C
Newcombe
SCAS
M−N
−5
0
5
Absolute risk reduction (%)
F14 Cornely 2012
Wald
A−C
Newcombe
SCAS
M−N
Reported by study
−10
−5
0
5
Absolute risk reduction (%)
F140 Villarino 2015
Wald
A−C
Newcombe
SCAS
M−N
Reported by study
−6
−4
−2
0
2
4
Absolute risk reduction (%)

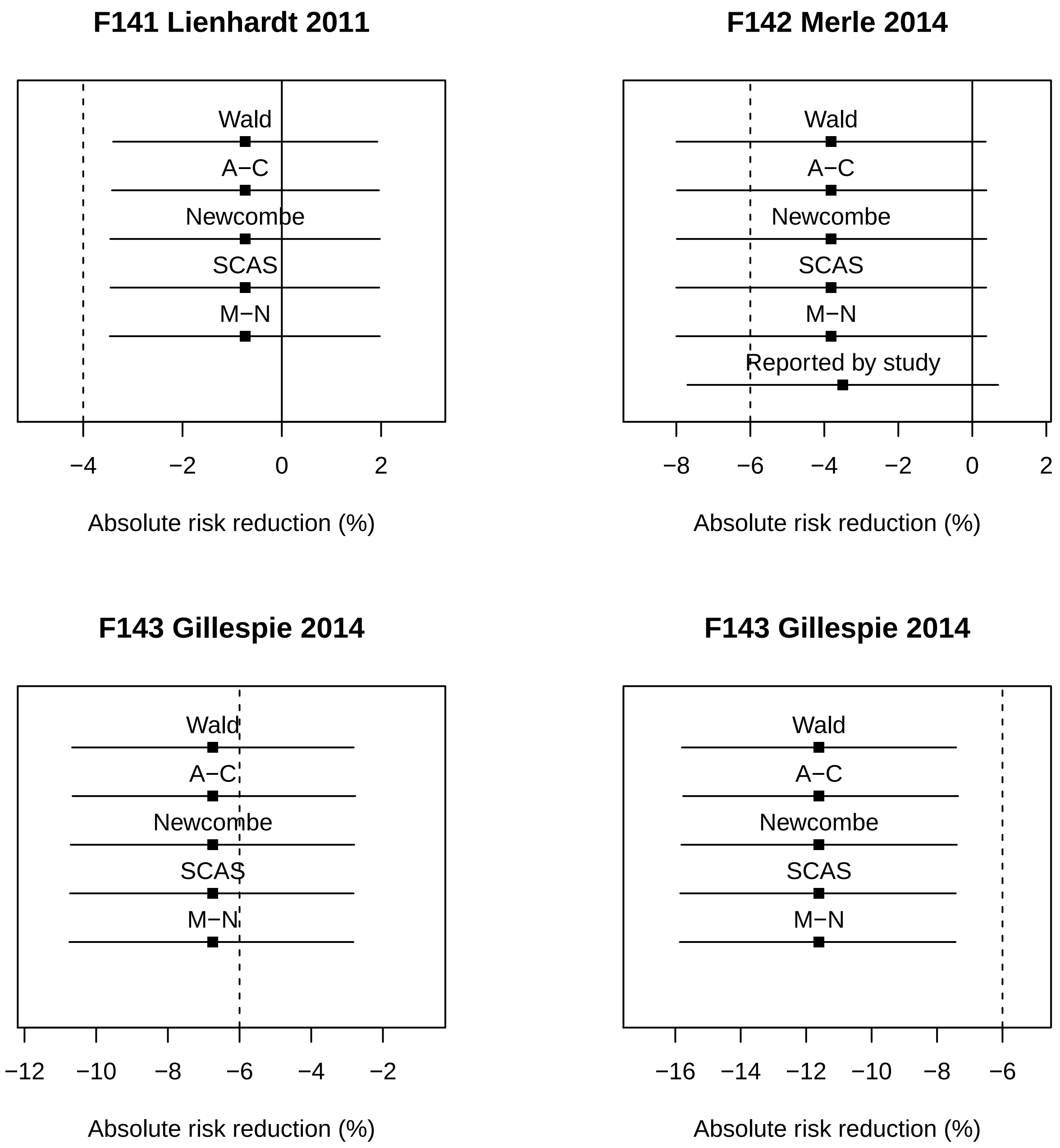
F141 Lienhardt 2011
Wald
A−C
Newcombe
SCAS
M−N
−4
−2
0
2
Absolute risk reduction (%)
F142 Merle 2014
Wald
A−C
Newcombe
SCAS
M−N
Reported by study
−8
−6
−4
−2
0
2
Absolute risk reduction (%)
F143 Gillespie 2014
Wald
A−C
Newcombe
SCAS
M−N
−12
−10
−8
−6
−4
−2
Absolute risk reduction (%)
F143 Gillespie 2014
Wald
A−C
Newcombe
SCAS
M−N
−16
−14
−12
−10
−8
−6
Absolute risk reduction (%)

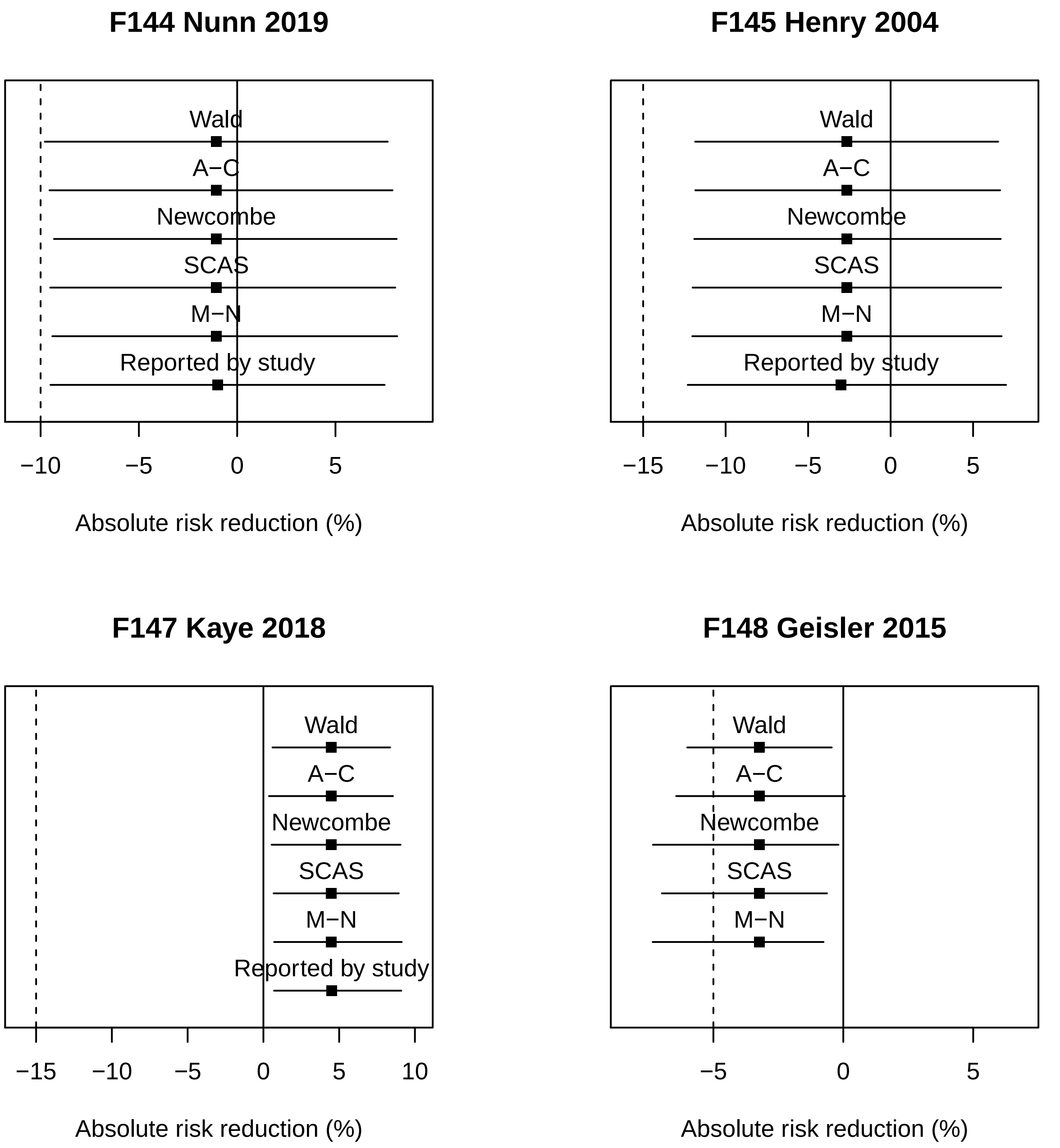

F144 Nunn 2019
Wald
A−C
Newcombe
SCAS
M−N
Reported by study
−10
−5
0
5
Absolute risk reduction (%)
F145 Henry 2004
Wald
A−C
Newcombe
SCAS
M−N
Reported by study
−15
−10
−5
0
5
Absolute risk reduction (%)
F147 Kaye 2018
Wald
A−C
Newcombe
SCAS
M−N
Reported by study
−15
−10
−5
0
5
10
Absolute risk reduction (%)
F148 Geisler 2015
Wald
A−C
Newcombe
SCAS
M−N
−5
0
5
Absolute risk reduction (%)

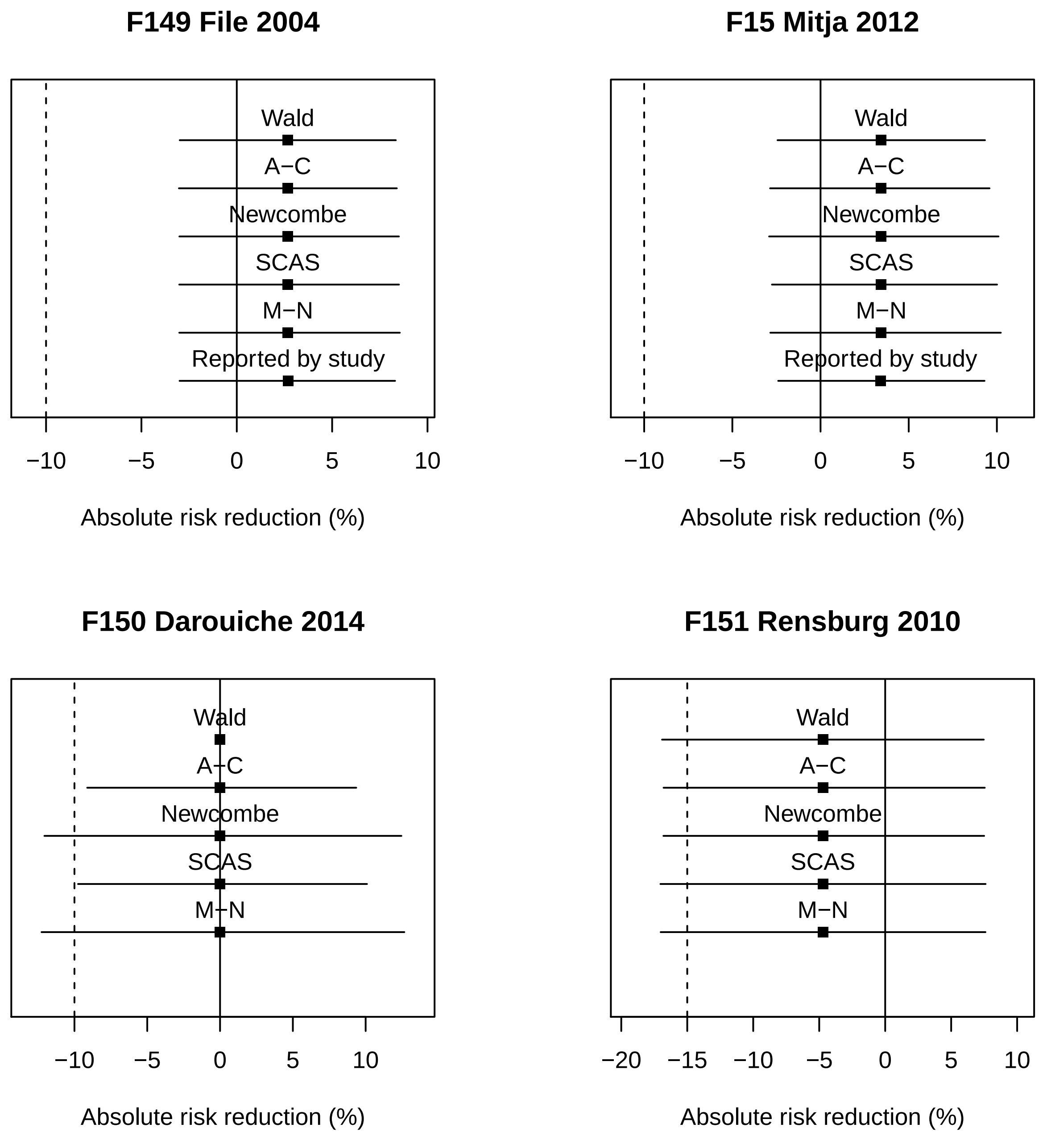
F149 File 2004
Wald
A−C
Newcombe
SCAS
M−N
Reported by study
−10
−5
0
5
10
Absolute risk reduction (%)
F15 Mitja 2012
Wald
A−C
Newcombe
SCAS
M−N
Reported by study
−10
−5
0
5
10
Absolute risk reduction (%)
F150 Darouiche 2014
Wald
A−C
Newcombe
SCAS
M−N
−10
−5
0
5
10
Absolute risk reduction (%)
F151 Rensburg 2010
Wald
A−C
Newcombe
SCAS
M−N
−20
−15
−10
−5
0
5
10
Absolute risk reduction (%)

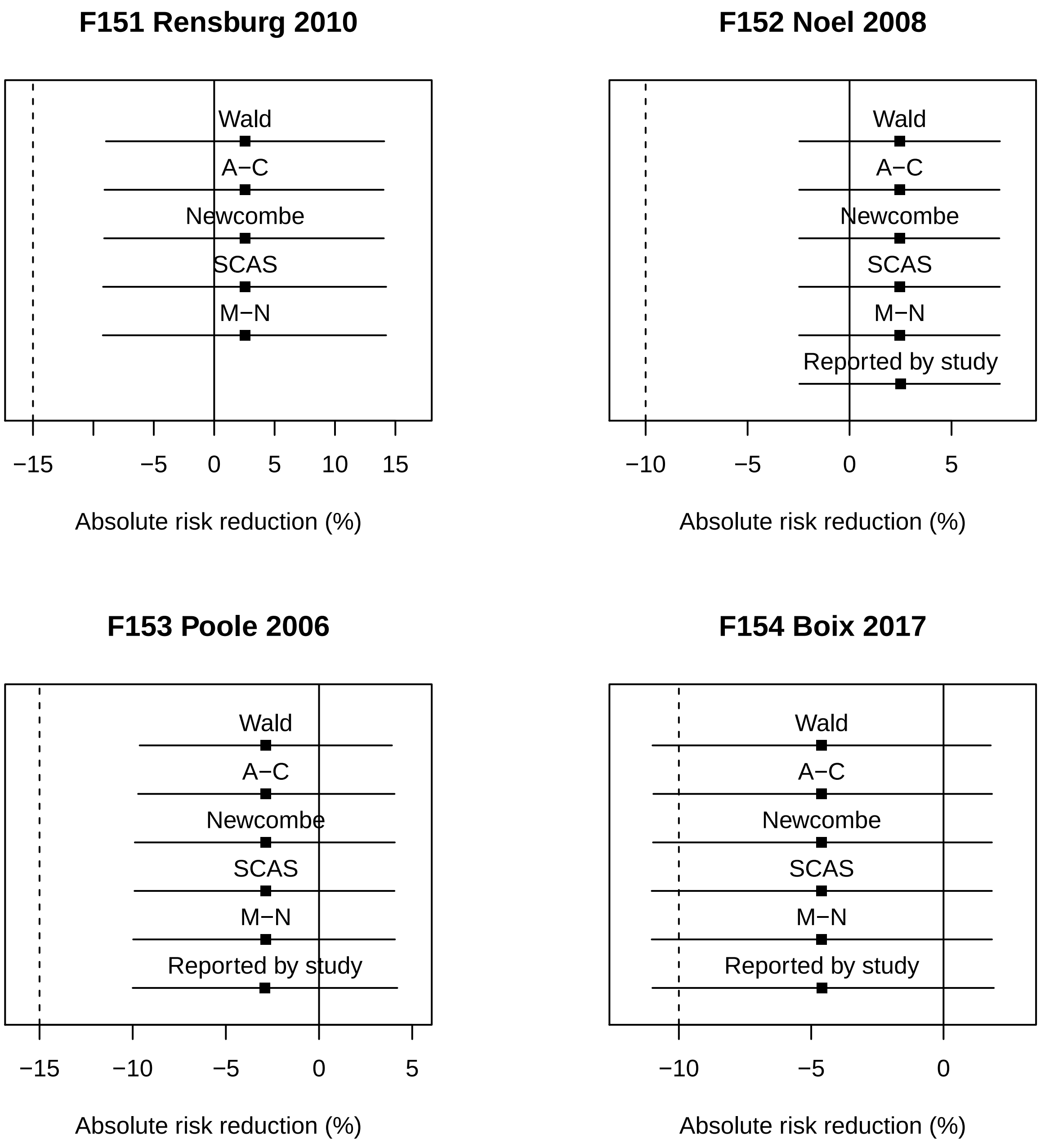
F151 Rensburg 2010
Wald
A−C
Newcombe
SCAS
M−N
−15
−5
0
5
10
15
Absolute risk reduction (%)
F152 Noel 2008
Wald
A−C
Newcombe
SCAS
M−N
Reported by study
−10
−5
0
5
Absolute risk reduction (%)
F153 Poole 2006
Wald
A−C
Newcombe
SCAS
M−N
Reported by study
−15
−10
−5
0
5
Absolute risk reduction (%)
F154 Boix 2017
Wald
A−C
Newcombe
SCAS
M−N
Reported by study
−10
−5
0
Absolute risk reduction (%)

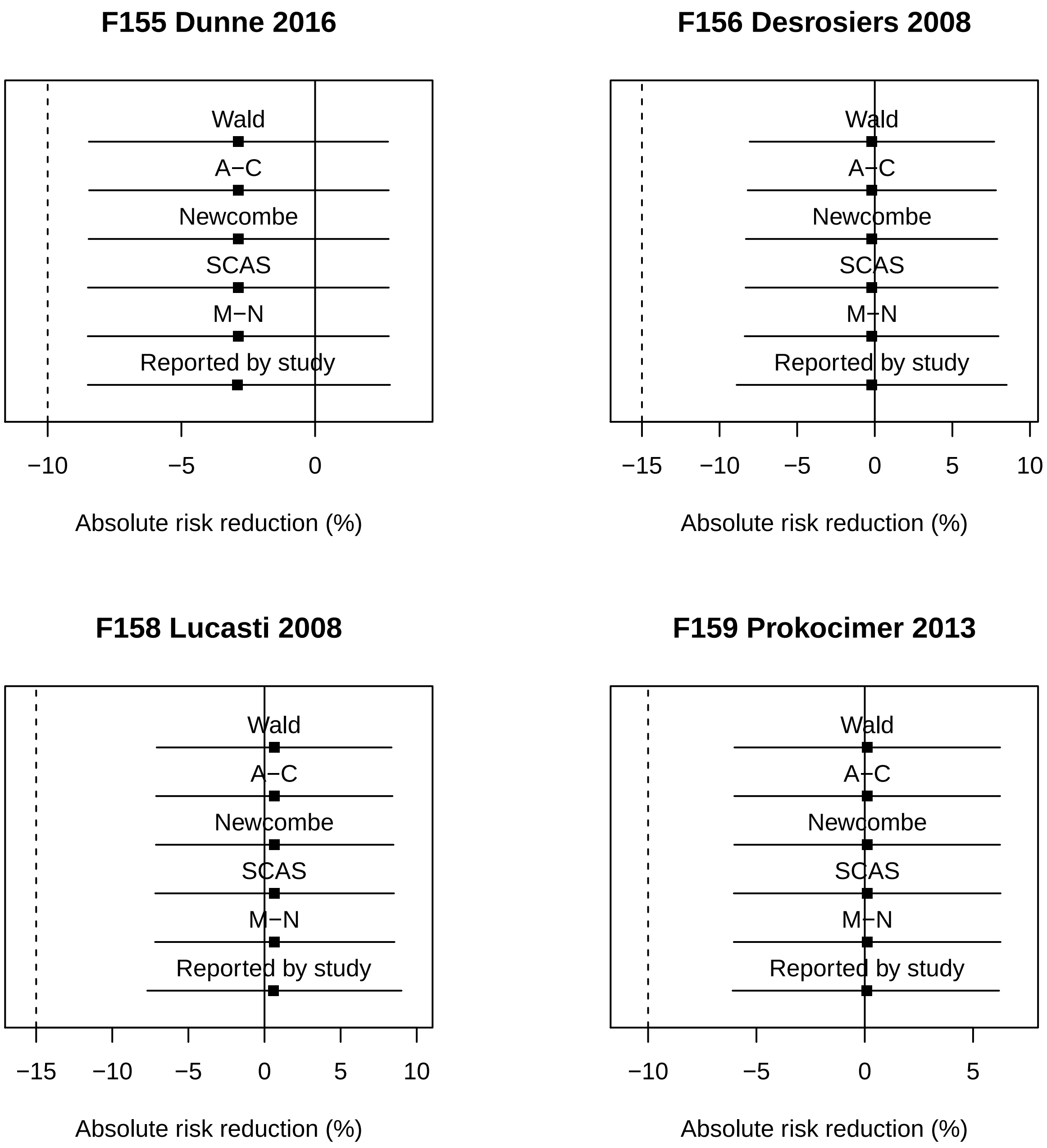
F155 Dunne 2016
Wald
A−C
Newcombe
SCAS
M−N
Reported by study
−10
−5
0
Absolute risk reduction (%)
F156 Desrosiers 2008
Wald
A−C
Newcombe
SCAS
M−N
Reported by study
−15
−10
−5
0
5
10
Absolute risk reduction (%)
F158 Lucasti 2008
Wald
A−C
Newcombe
SCAS
M−N
Reported by study
−15
−10
−5
0
5
10
Absolute risk reduction (%)
F159 Prokocimer 2013
Wald
A−C
Newcombe
SCAS
M−N
Reported by study
−10
−5
0
5
Absolute risk reduction (%)

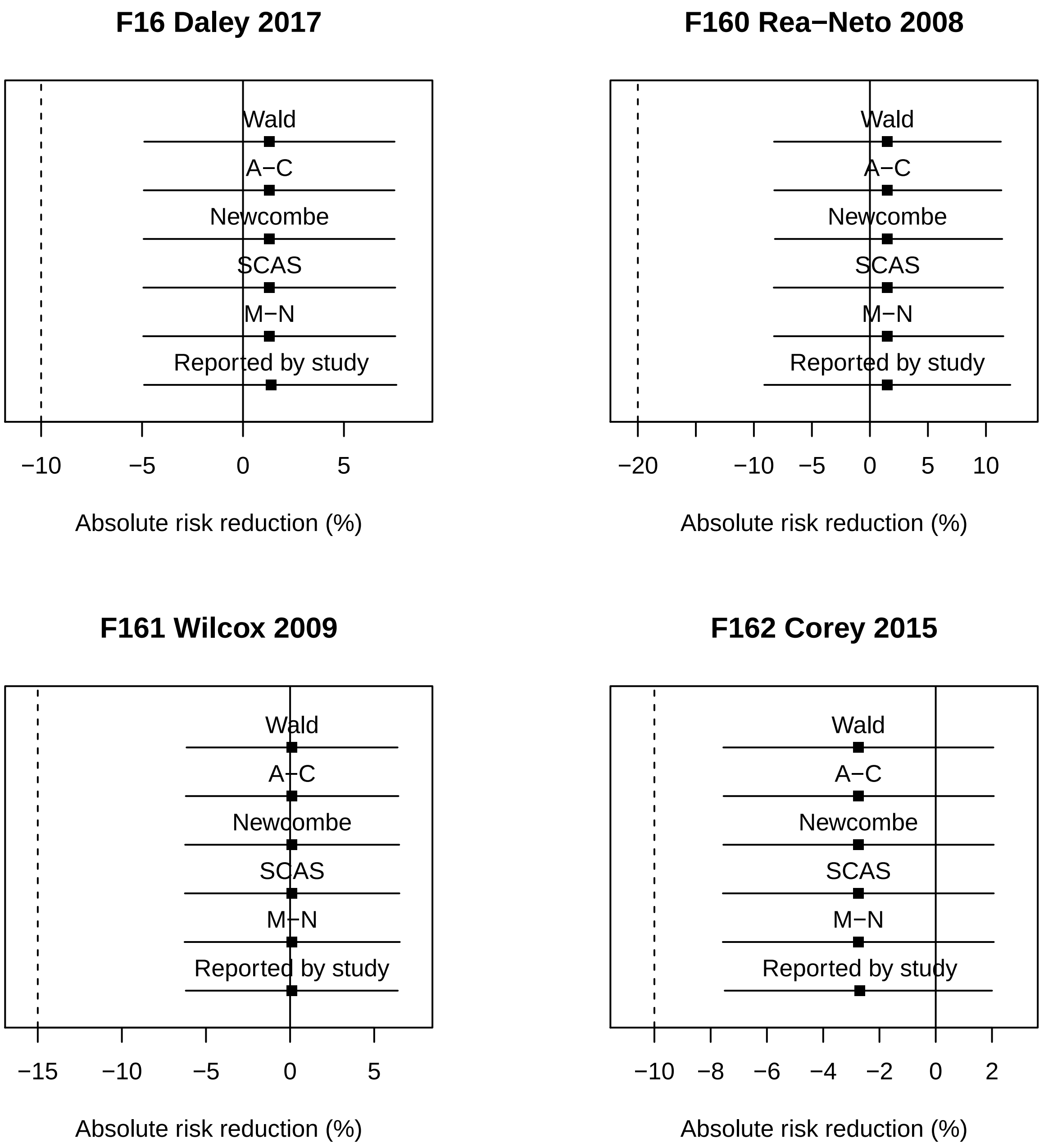

F16 Daley 2017
Wald
A−C
Newcombe
SCAS
M−N
Reported by study
−10
−5
0
5
Absolute risk reduction (%)
F160 Rea−Neto 2008
Wald
A−C
Newcombe
SCAS
M−N
Reported by study
−20
−10
−5
0
5
10
Absolute risk reduction (%)
F161 Wilcox 2009
Wald
A−C
Newcombe
SCAS
M−N
Reported by study
−15
−10
−5
0
5
Absolute risk reduction (%)
F162 Corey 2015
Wald
A−C
Newcombe
SCAS
M−N
Reported by study
−10
−8
−6
−4
−2
0
2
Absolute risk reduction (%)

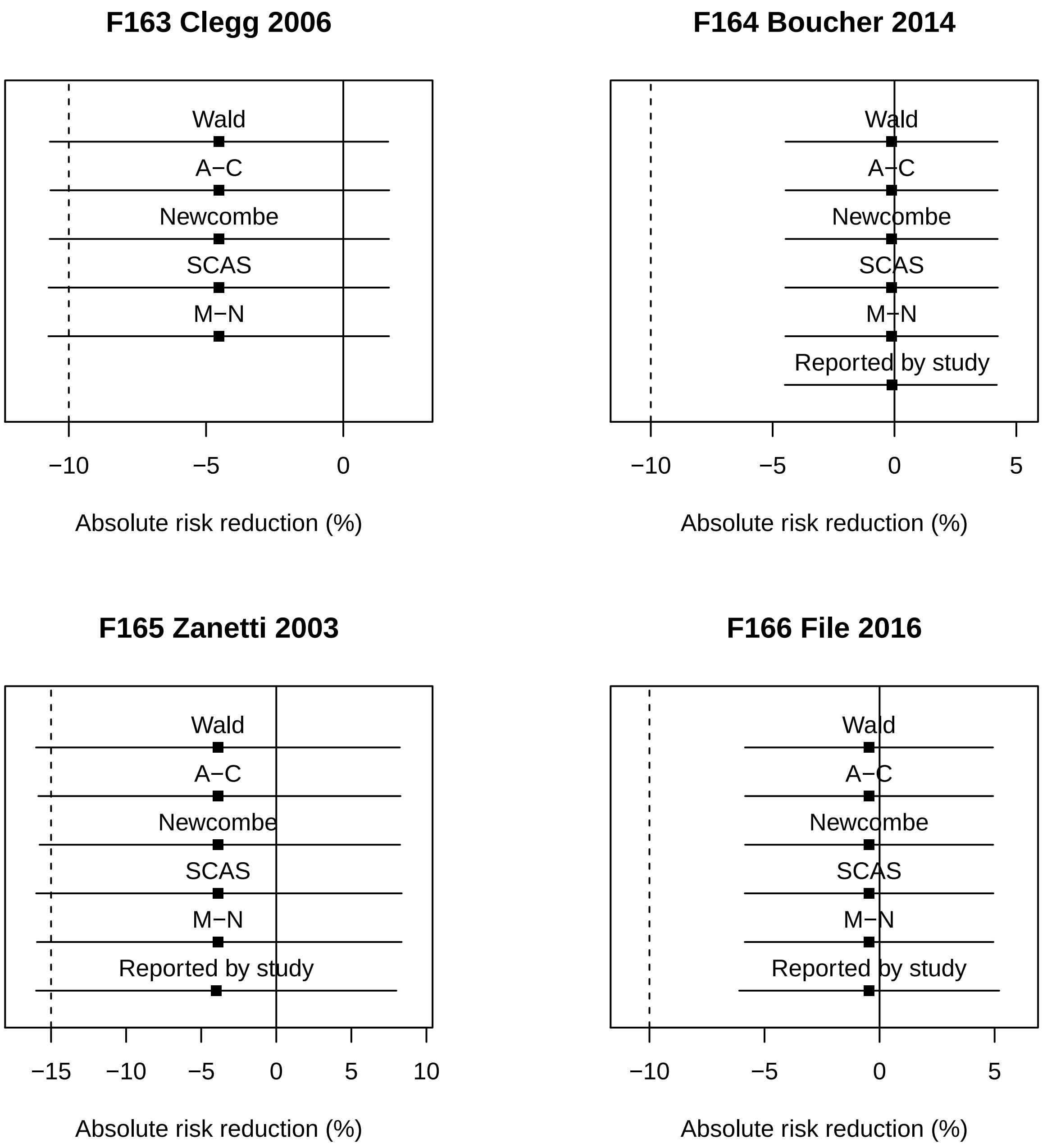
F163 Clegg 2006
Wald
A−C
Newcombe
SCAS
M−N
−10
−5
0
Absolute risk reduction (%)
F164 Boucher 2014
Wald
A−C
Newcombe
SCAS
M−N
Reported by study
−10
−5
0
5
Absolute risk reduction (%)
F165 Zanetti 2003
Wald
A−C
Newcombe
SCAS
M−N
Reported by study
−15
−10
−5
0
5
10
Absolute risk reduction (%)
F166 File 2016
Wald
A−C
Newcombe
SCAS
M−N
Reported by study
−10
−5
0
5
Absolute risk reduction (%)

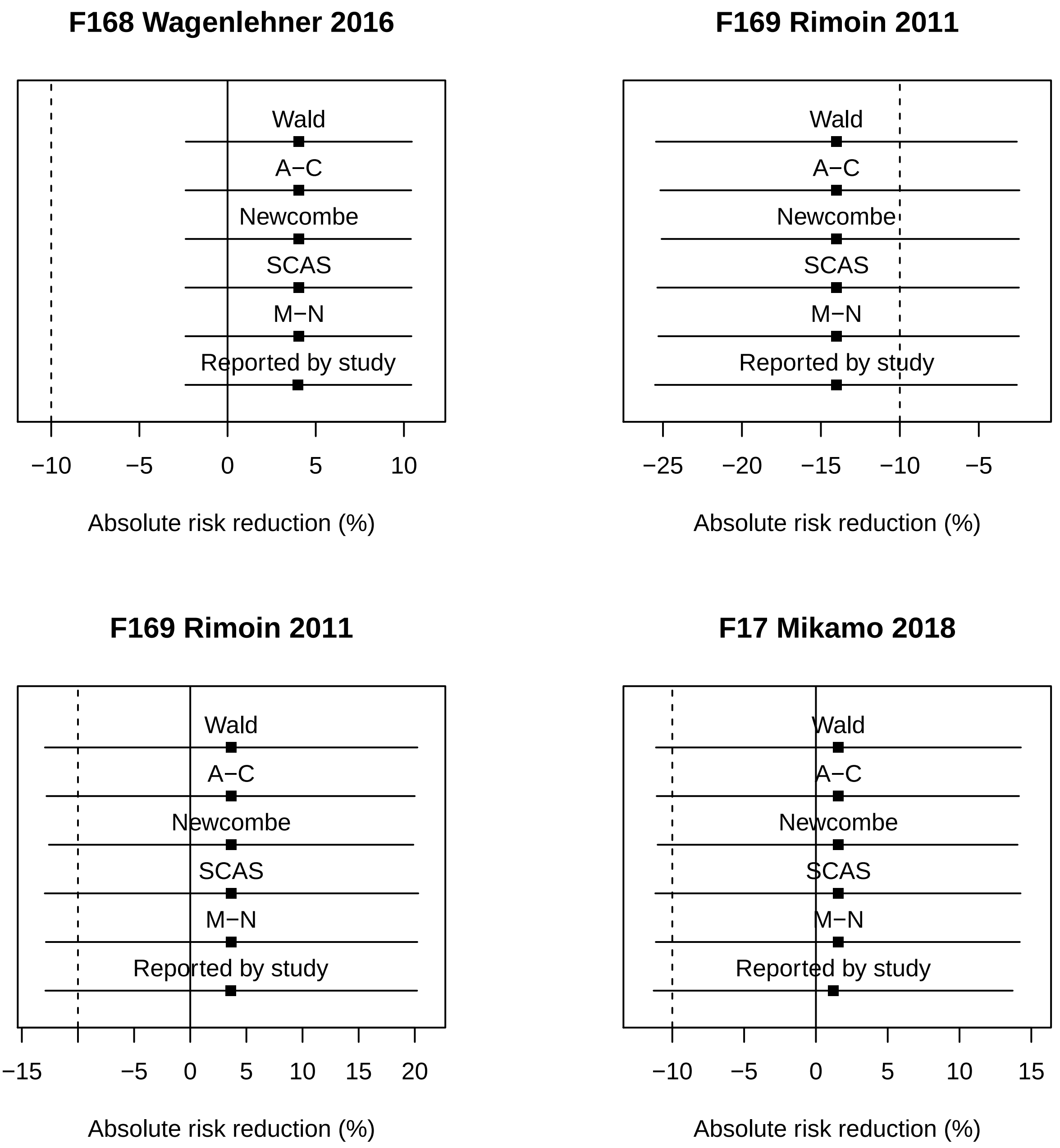
F168 Wagenlehner 2016
Wald
A−C
Newcombe
SCAS
M−N
Reported by study
−10
−5
0
5
10
Absolute risk reduction (%)
F169 Rimoin 2011
Wald
A−C
Newcombe
SCAS
M−N
Reported by study
−25
−20
−15
−10
−5
Absolute risk reduction (%)
F169 Rimoin 2011
Wald
A−C
Newcombe
SCAS
M−N
Reported by study
−15
−5
0
5
10
15
20
Absolute risk reduction (%)
F17 Mikamo 2018
Wald
A−C
Newcombe
SCAS
M−N
Reported by study
−10
−5
0
5
10
15
Absolute risk reduction (%)

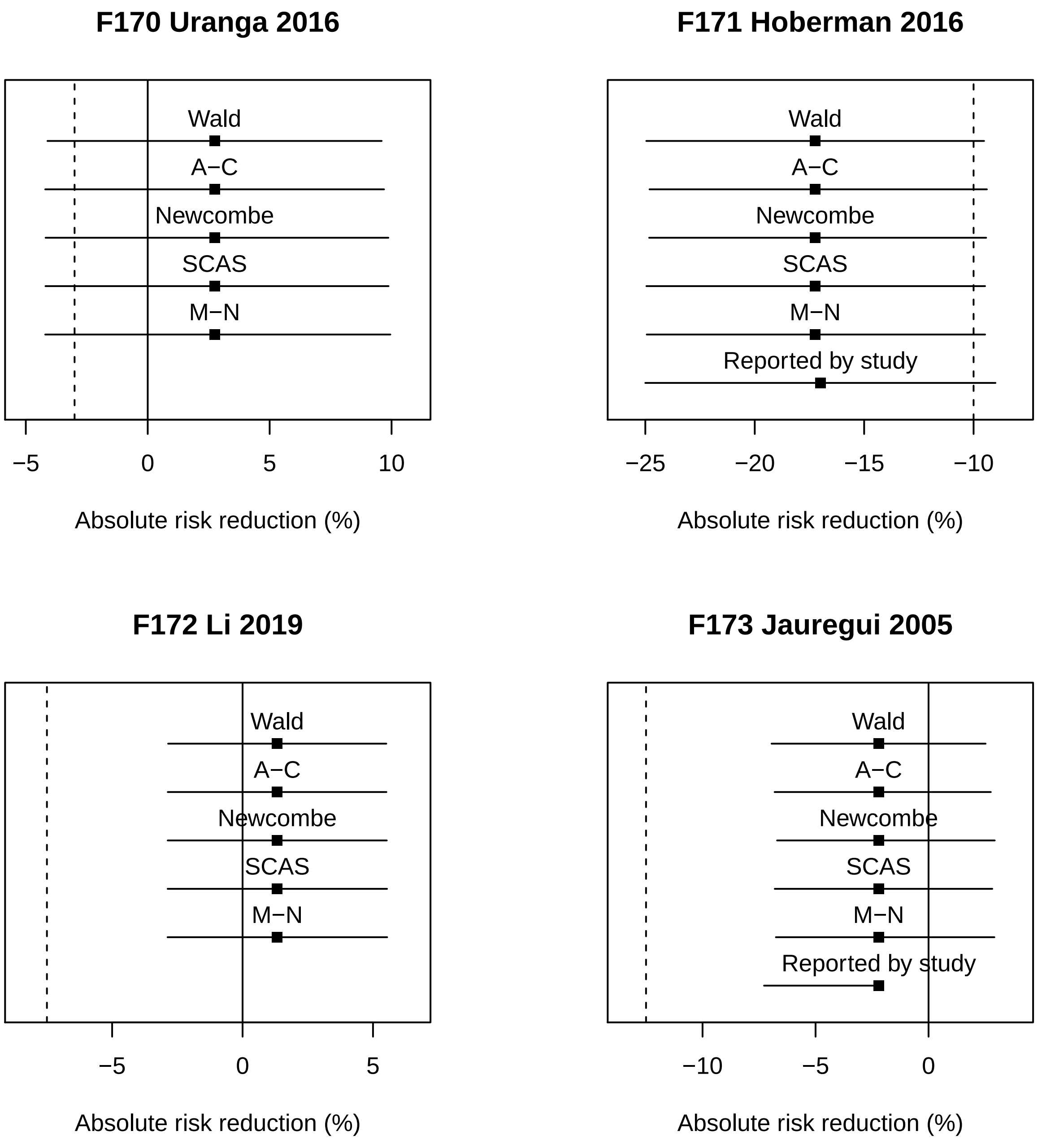
F170 Uranga 2016
Wald
A−C
Newcombe
SCAS
M−N
−5
0
5
10
Absolute risk reduction (%)
F171 Hoberman 2016
Wald
A−C
Newcombe
SCAS
M−N
Reported by study
−25
−20
−15
−10
Absolute risk reduction (%)
F172 Li 2019
Wald
A−C
Newcombe
SCAS
M−N
−5
0
5
Absolute risk reduction (%)
F173 Jauregui 2005
Wald
A−C
Newcombe
SCAS
M−N
Reported by study
−10
−5
0
Absolute risk reduction (%)

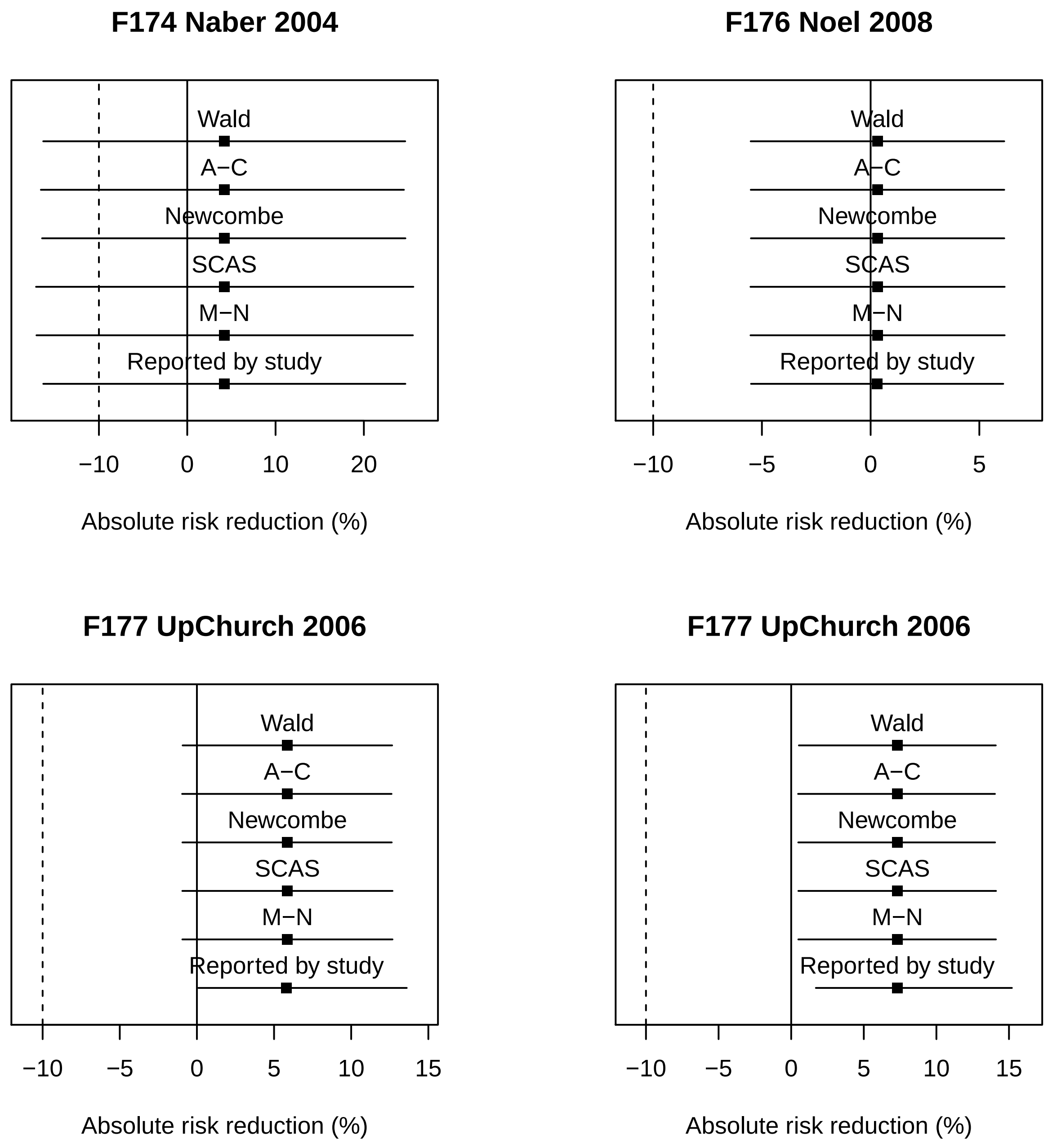
F174 Naber 2004
Wald
A−C
Newcombe
SCAS
M−N
Reported by study
−10
0
10
20
Absolute risk reduction (%)
F176 Noel 2008
Wald
A−C
Newcombe
SCAS
M−N
Reported by study
−10
−5
0
5
Absolute risk reduction (%)
F177 UpChurch 2006
Wald
A−C
Newcombe
SCAS
M−N
Reported by study
−10
−5
0
5
10
15
Absolute risk reduction (%)
F177 UpChurch 2006
Wald
A−C
Newcombe
SCAS
M−N
Reported by study
−10
−5
0
5
10
15
Absolute risk reduction (%)

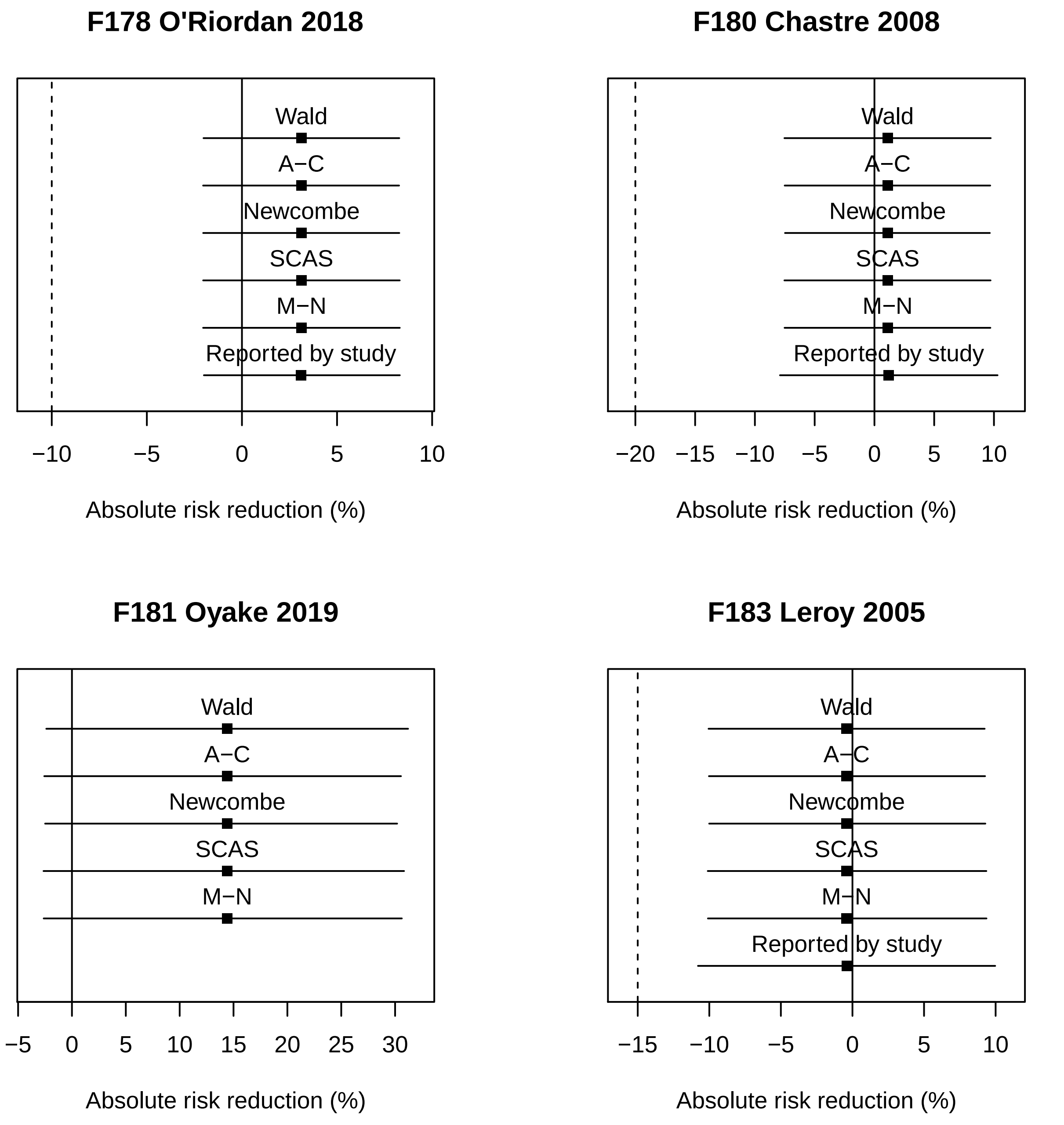
F178 O'Riordan 2018
Wald
A−C
Newcombe
SCAS
M−N
Reported by study
−10
−5
0
5
10
Absolute risk reduction (%)
F180 Chastre 2008
Wald
A−C
Newcombe
SCAS
M−N
Reported by study
−20
−15
−10
−5
0
5
10
Absolute risk reduction (%)
F181 Oyake 2019
Wald
A−C
Newcombe
SCAS
M−N
−5
0
5
10
15
20
25
30
Absolute risk reduction (%)
F183 Leroy 2005
Wald
A−C
Newcombe
SCAS
M−N
Reported by study
−15
−10
−5
0
5
10
Absolute risk reduction (%)

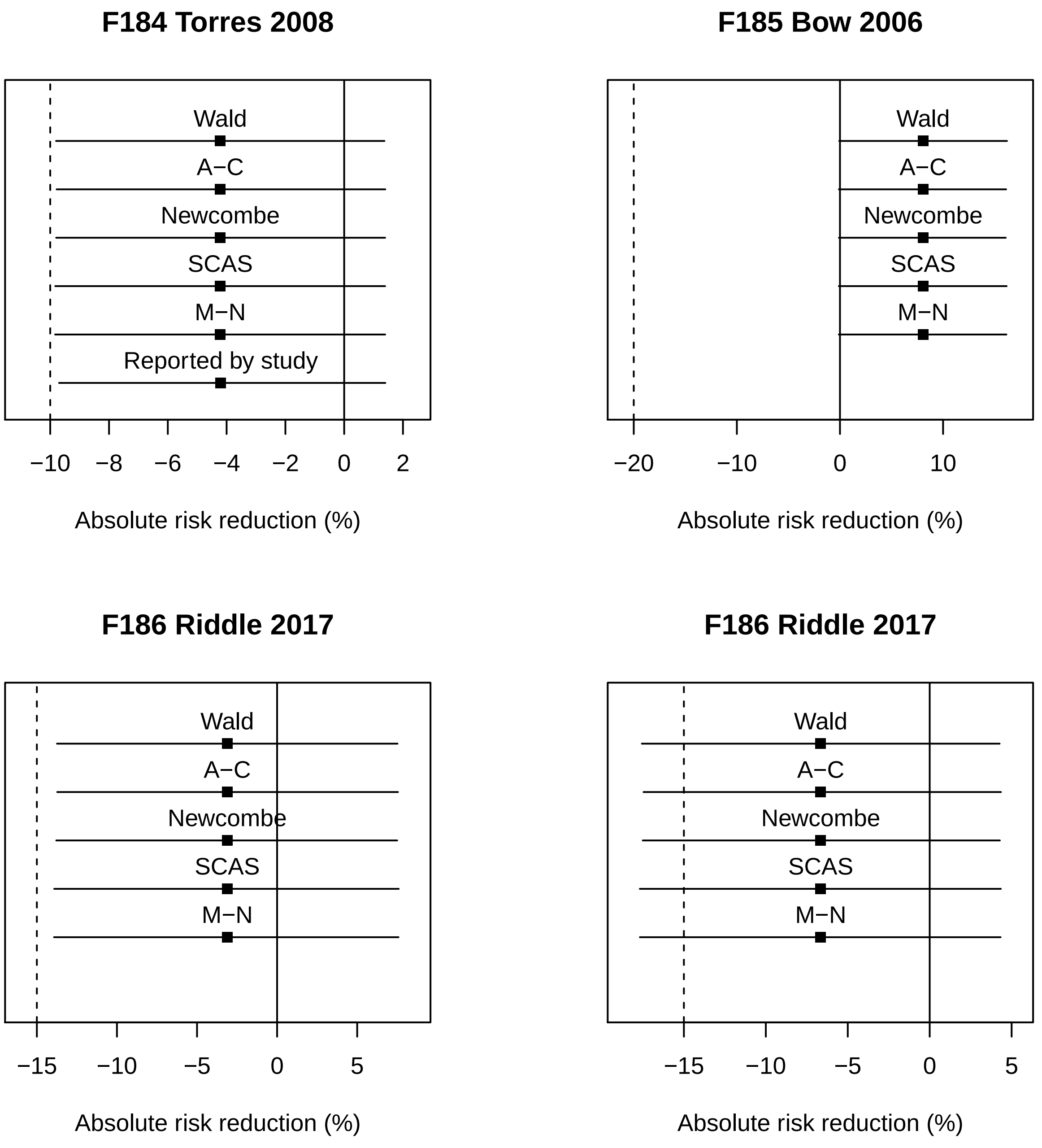

F184 Torres 2008
Wald
A−C
Newcombe
SCAS
M−N
Reported by study
−10
−8
−6
−4
−2
0
2
Absolute risk reduction (%)
F185 Bow 2006
Wald
A−C
Newcombe
SCAS
M−N
−20
−10
0
10
Absolute risk reduction (%)
F186 Riddle 2017
Wald
A−C
Newcombe
SCAS
M−N
−15
−10
−5
0
5
Absolute risk reduction (%)
F186 Riddle 2017
Wald
A−C
Newcombe
SCAS
M−N
−15
−10
−5
0
5
Absolute risk reduction (%)

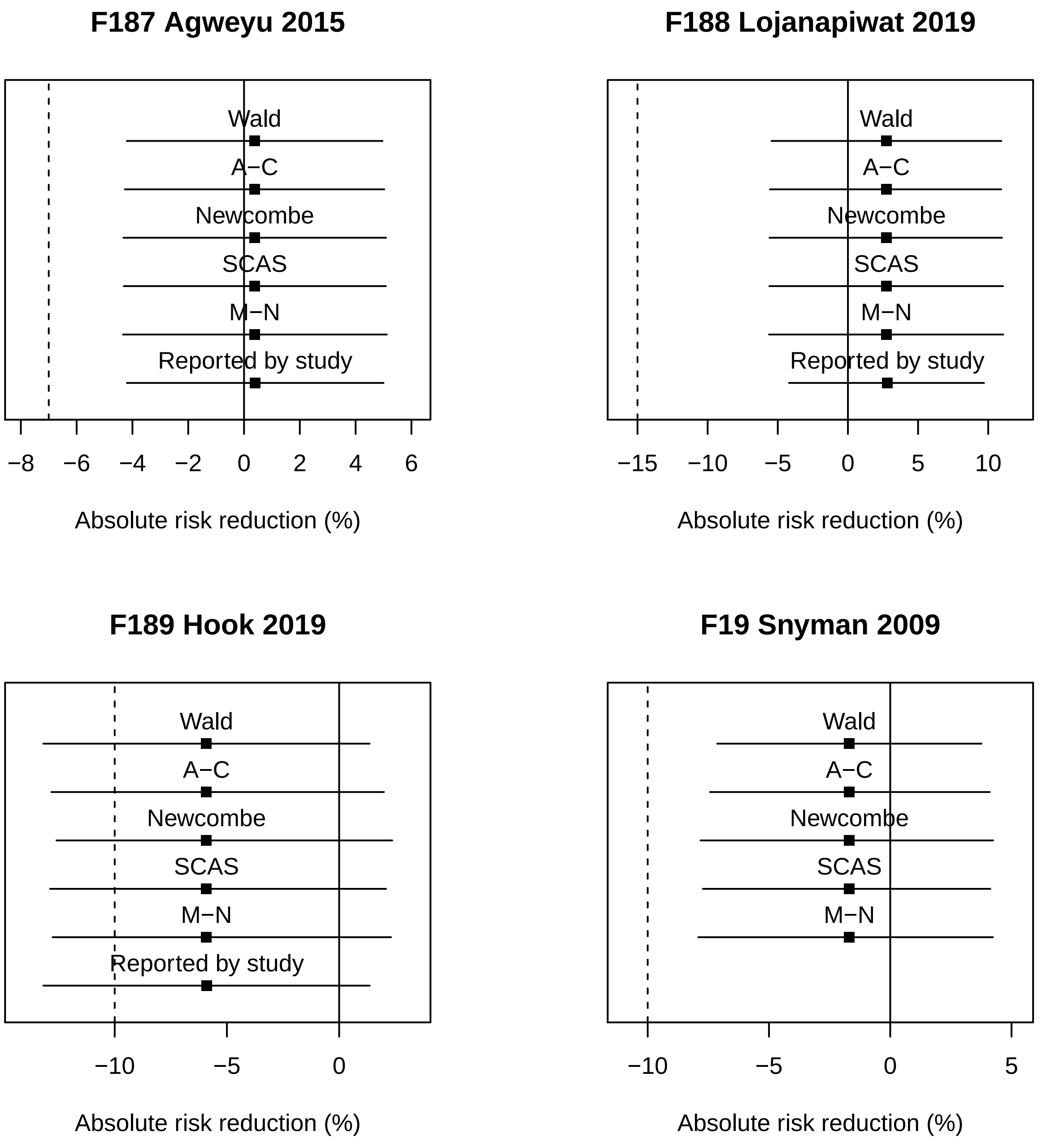
F187 Agweyu 2015
Wald
A−C
Newcombe
SCAS
M−N
Reported by study
−8
−6
−4
−2
0
2
4
6
Absolute risk reduction (%)
F188 Lojanapiwat 2019
Wald
A−C
Newcombe
SCAS
M−N
Reported by study
−15
−10
−5
0
5
10
Absolute risk reduction (%)
F189 Hook 2019
Wald
A−C
Newcombe
SCAS
M−N
Reported by study
−10
−5
0
Absolute risk reduction (%)
F19 Snyman 2009
Wald
A−C
Newcombe
SCAS
M−N
−10
−5
0
5
Absolute risk reduction (%)

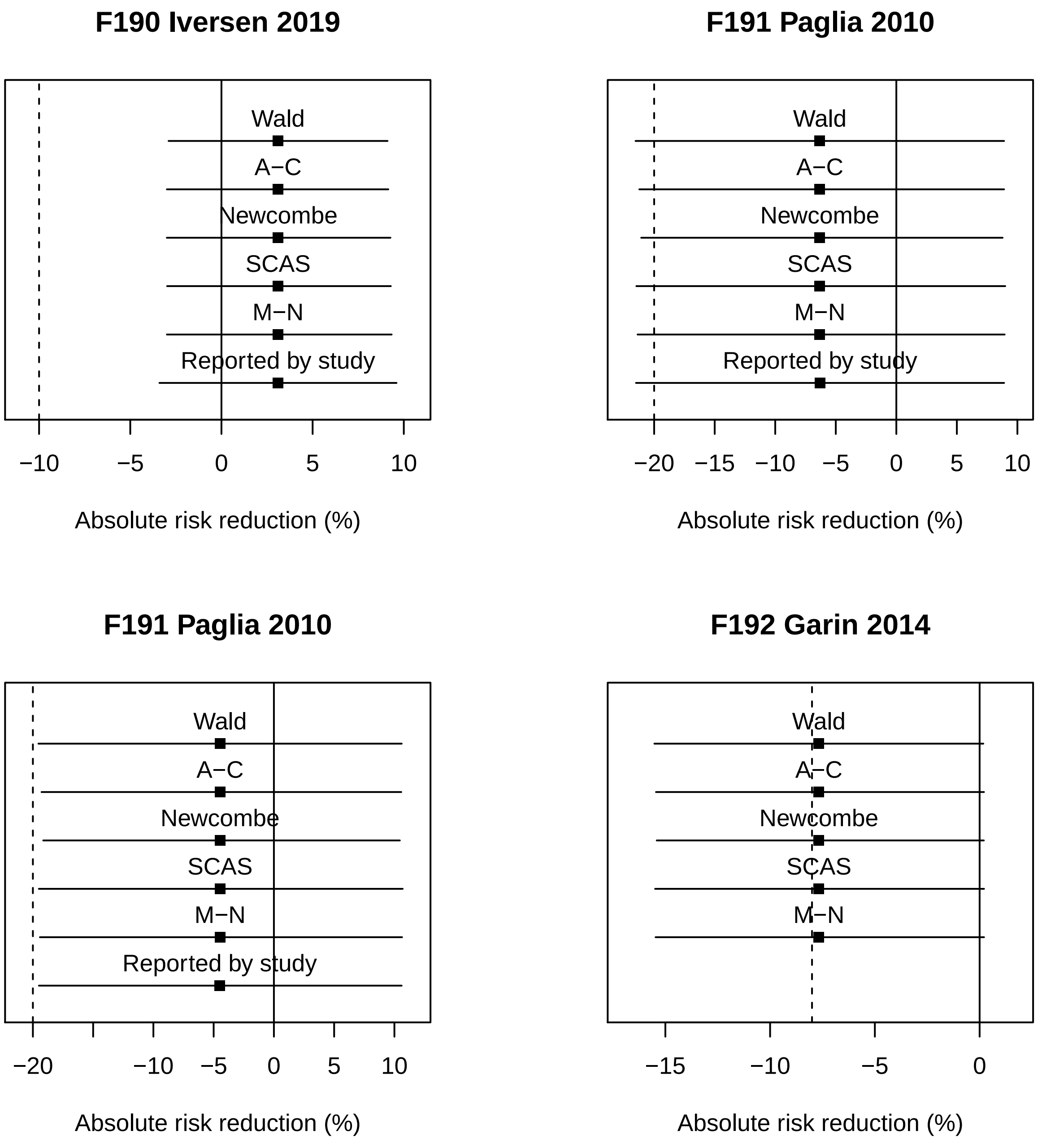
F190 Iversen 2019
Wald
A−C
Newcombe
SCAS
M−N
Reported by study
−10
−5
0
5
10
Absolute risk reduction (%)
F191 Paglia 2010
Wald
A−C
Newcombe
SCAS
M−N
Reported by study
−20
−15
−10
−5
0
5
10
Absolute risk reduction (%)
F191 Paglia 2010
Wald
A−C
Newcombe
SCAS
M−N
Reported by study
−20
−10
−5
0
5
10
Absolute risk reduction (%)
F192 Garin 2014
Wald
A−C
Newcombe
SCAS
M−N
−15
−10
−5
0
Absolute risk reduction (%)

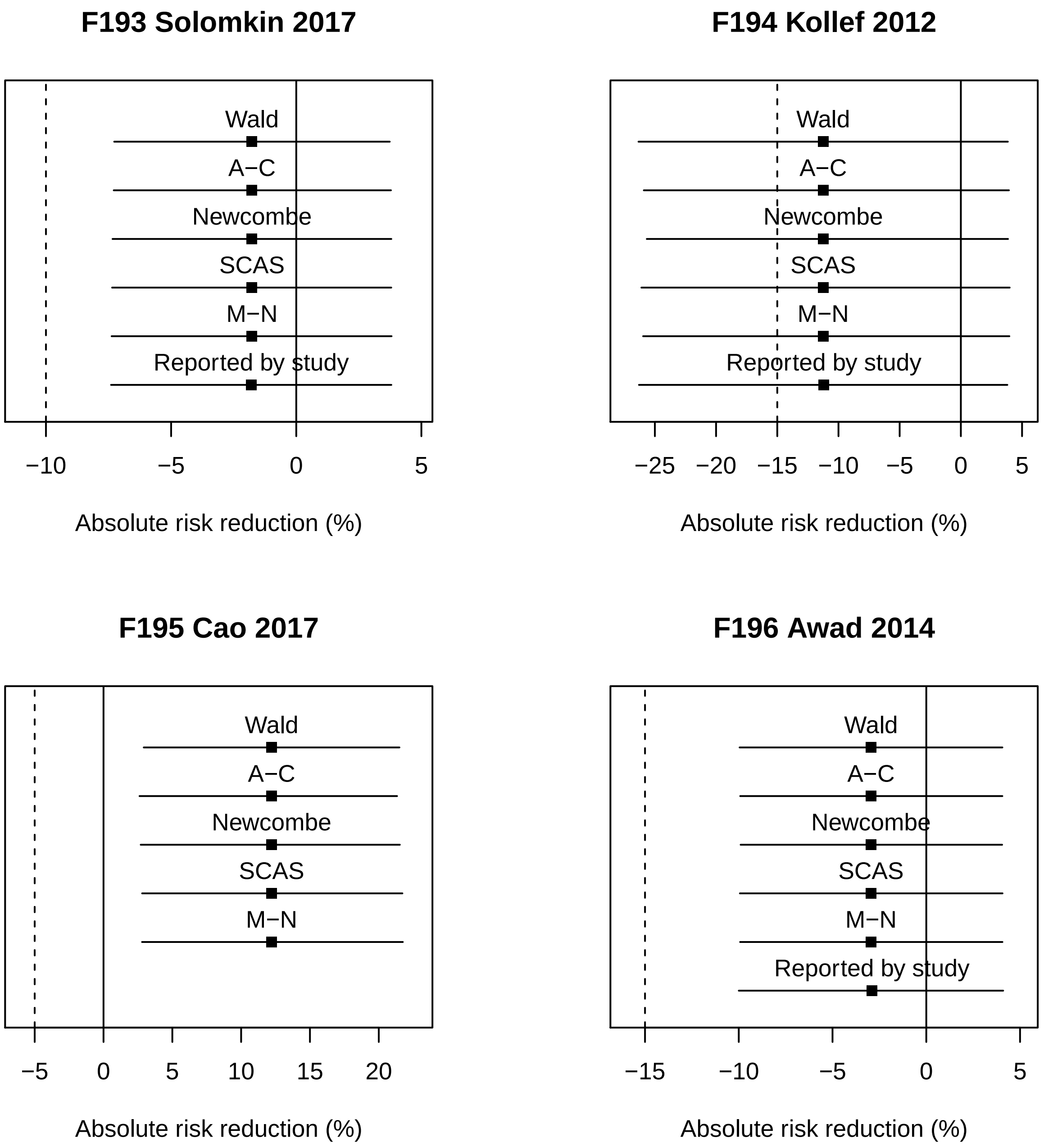

F193 Solomkin 2017
Wald
A−C
Newcombe
SCAS
M−N
Reported by study
−10
−5
0
5
Absolute risk reduction (%)
F194 Kollef 2012
Wald
A−C
Newcombe
SCAS
M−N
Reported by study
−25
−20
−15
−10
−5
0
5
Absolute risk reduction (%)
F195 Cao 2017
Wald
A−C
Newcombe
SCAS
M−N
−5
0
5
10
15
20
Absolute risk reduction (%)
F196 Awad 2014
Wald
A−C
Newcombe
SCAS
M−N
Reported by study
−15
−10
−5
0
5
Absolute risk reduction (%)

F197 Yoon 2013

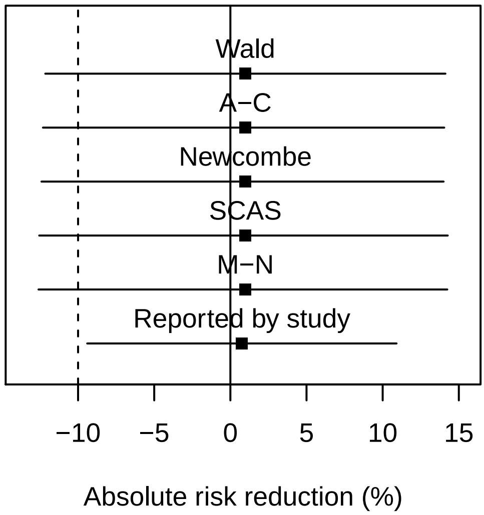

F198 Bradley 2007

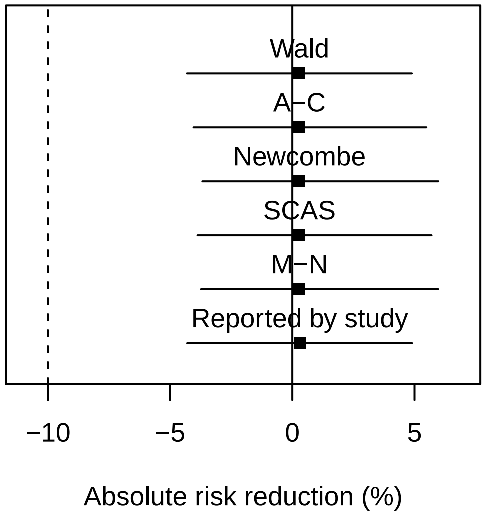

F2 van Nieuwkoop 2017

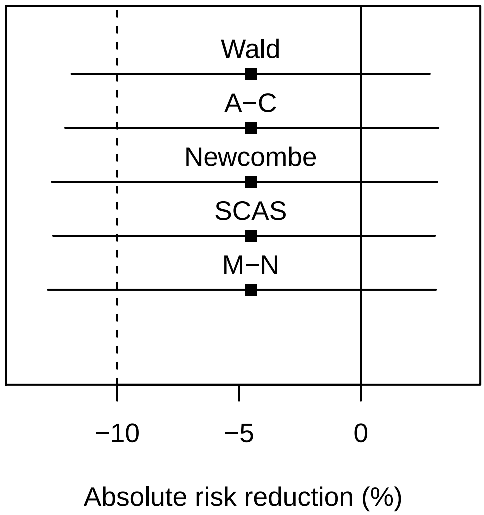

F20 Moran 2014

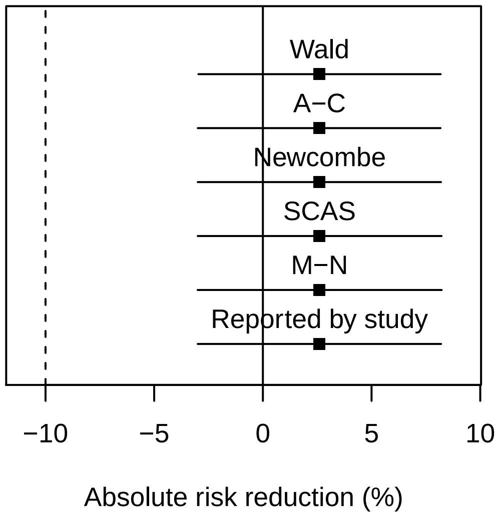

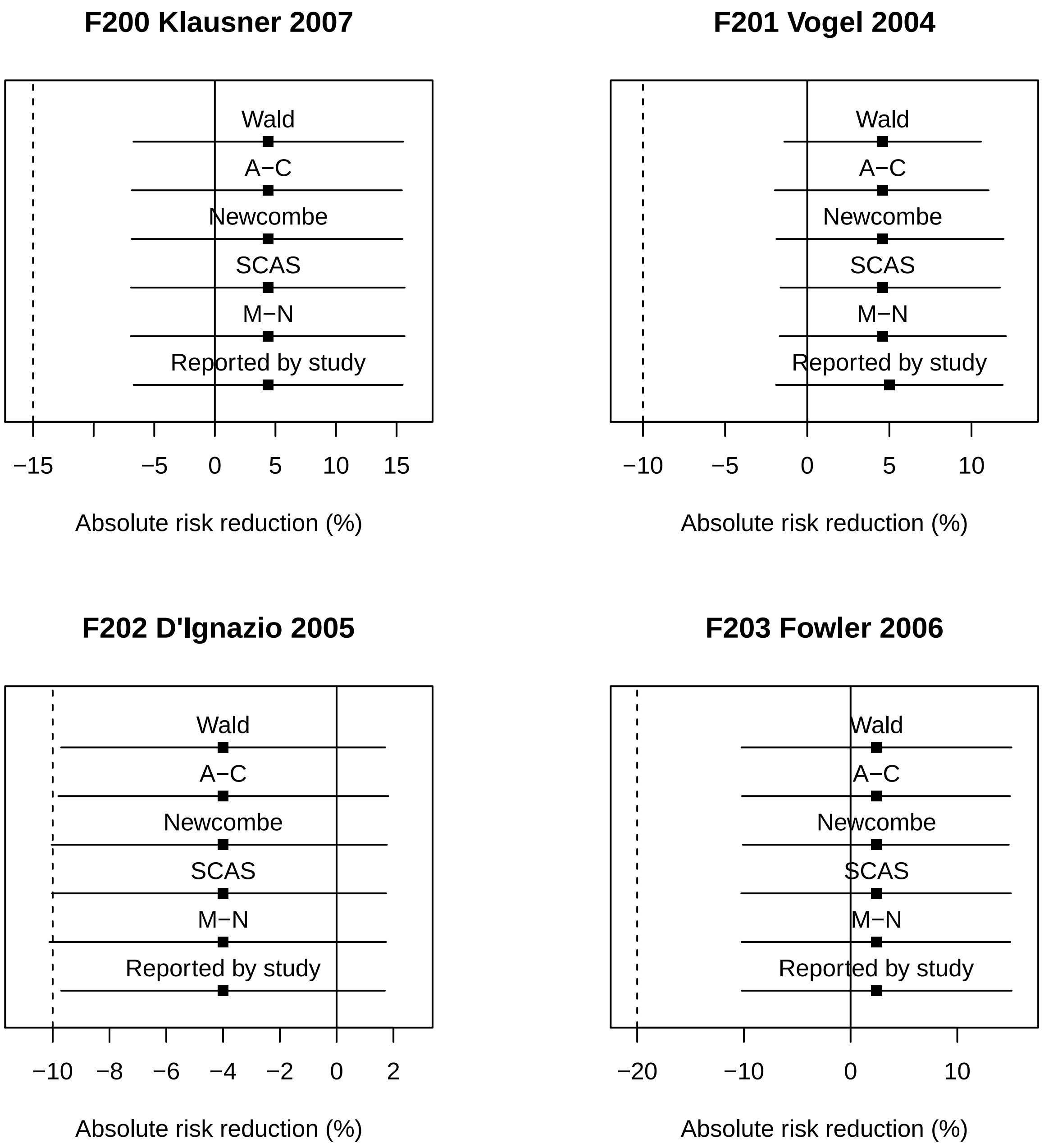

F200 Klausner 2007
Wald
A−C
Newcombe
SCAS
M−N
Reported by study
−15
−5
0
5
10
15
Absolute risk reduction (%)
F201 Vogel 2004
Wald
A−C
Newcombe
SCAS
M−N
Reported by study
−10
−5
0
5
10
Absolute risk reduction (%)
F202 D'Ignazio 2005
Wald
A−C
Newcombe
SCAS
M−N
Reported by study
−10
−8
−6
−4
−2
0
2
Absolute risk reduction (%)
F203 Fowler 2006
Wald
A−C
Newcombe
SCAS
M−N
Reported by study
−20
−10
0
10
Absolute risk reduction (%)

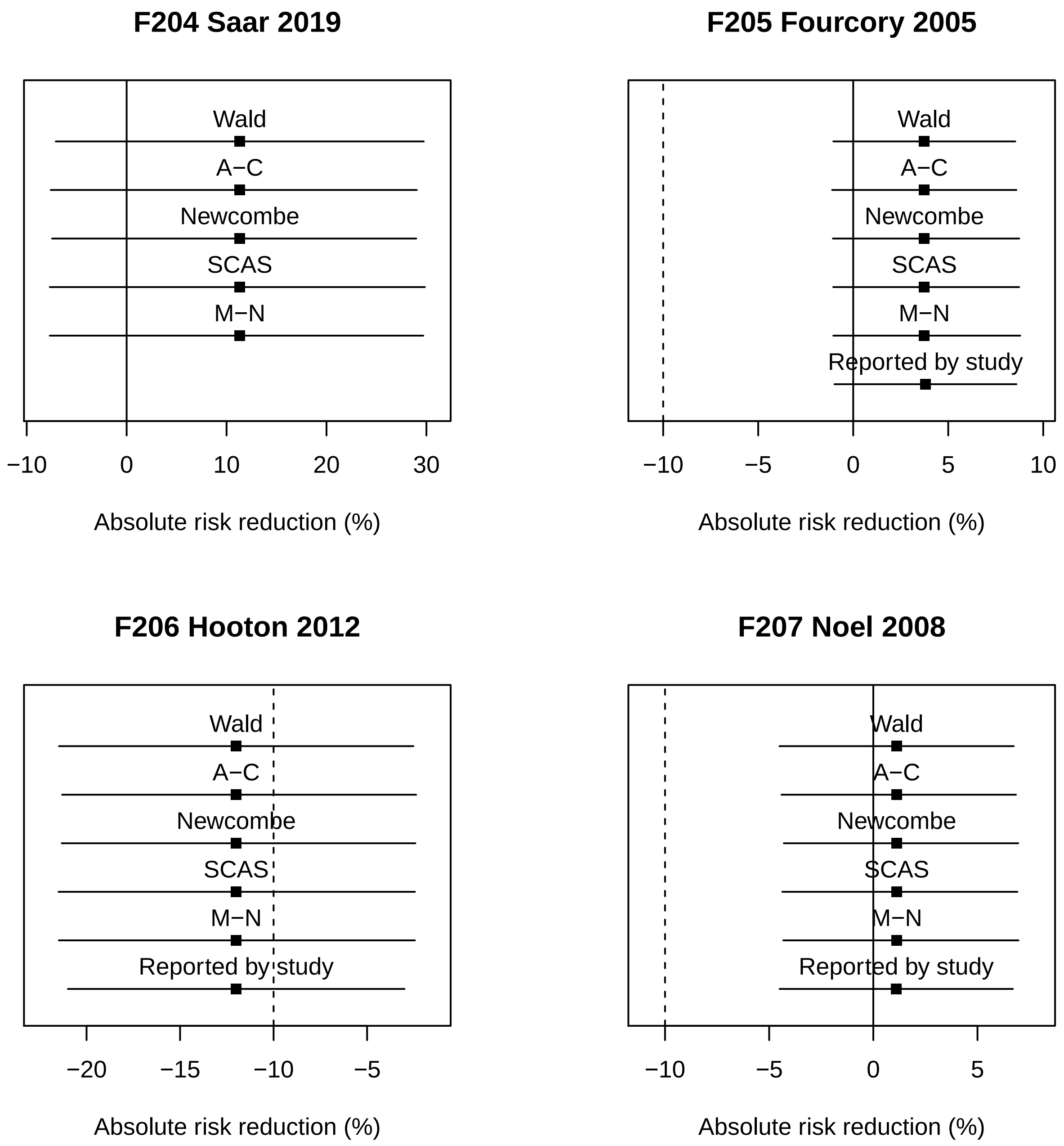

F204 Saar 2019
Wald
A−C
Newcombe
SCAS
M−N
−10
0
10
20
30
Absolute risk reduction (%)
F205 Fourcory 2005
Wald
A−C
Newcombe
SCAS
M−N
Reported by study
−10
−5
0
5
10
Absolute risk reduction (%)
F206 Hooton 2012
Wald
A−C
Newcombe
SCAS
M−N
Reported by study
−20
−15
−10
−5
Absolute risk reduction (%)
F207 Noel 2008
Wald
A−C
Newcombe
SCAS
M−N
Reported by study
−10
−5
0
5
Absolute risk reduction (%)

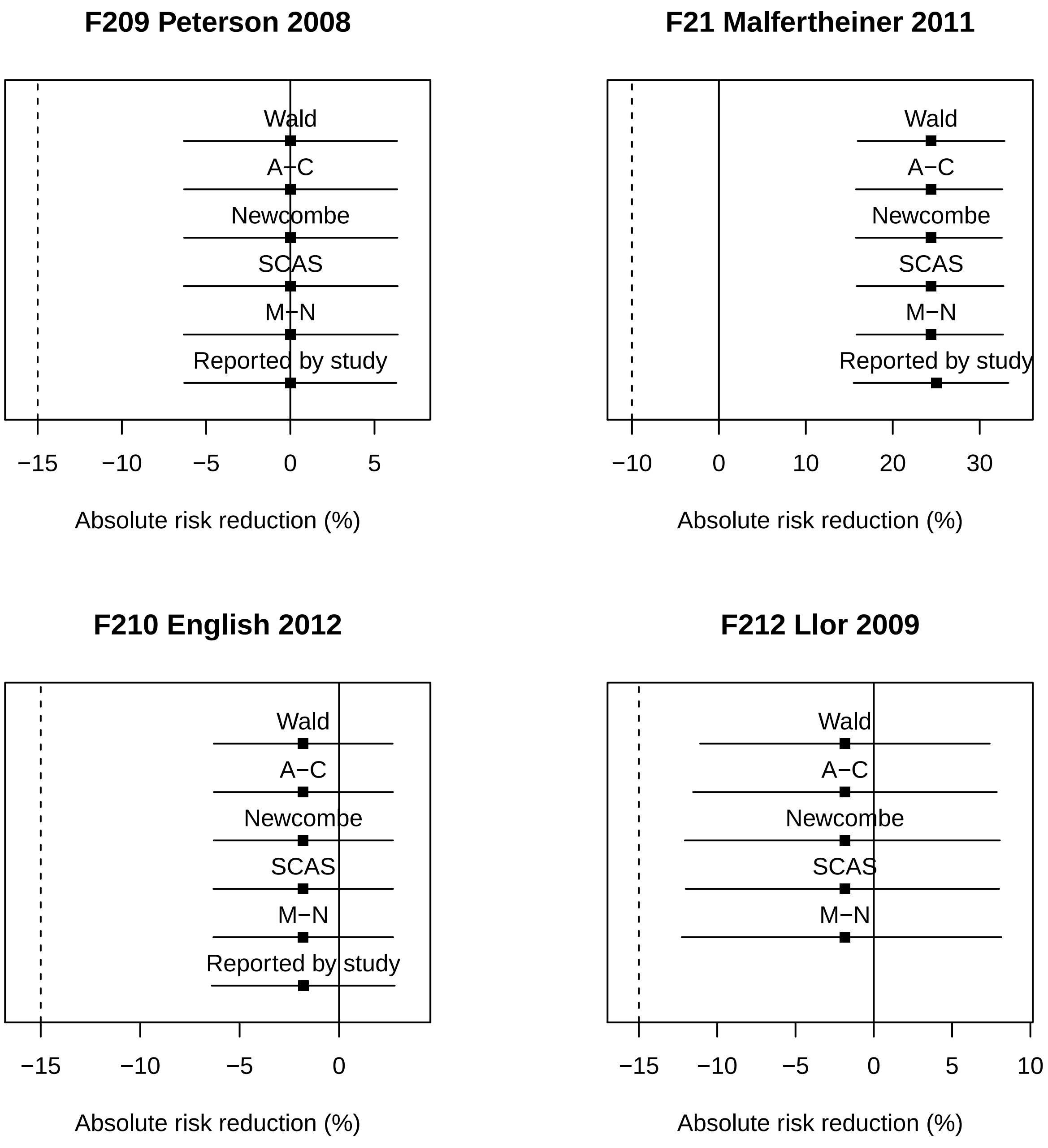
F209 Peterson 2008
Wald
A−C
Newcombe
SCAS
M−N
Reported by study
−15
−10
−5
0
5
Absolute risk reduction (%)
F21 Malfertheiner 2011
Wald
A−C
Newcombe
SCAS
M−N
Reported by study
−10
0
10
20
30
Absolute risk reduction (%)
F210 English 2012
Wald
A−C
Newcombe
SCAS
M−N
Reported by study
−15
−10
−5
0
Absolute risk reduction (%)
F212 Llor 2009
Wald
A−C
Newcombe
SCAS
M−N
−15
−10
−5
0
5
10
Absolute risk reduction (%)

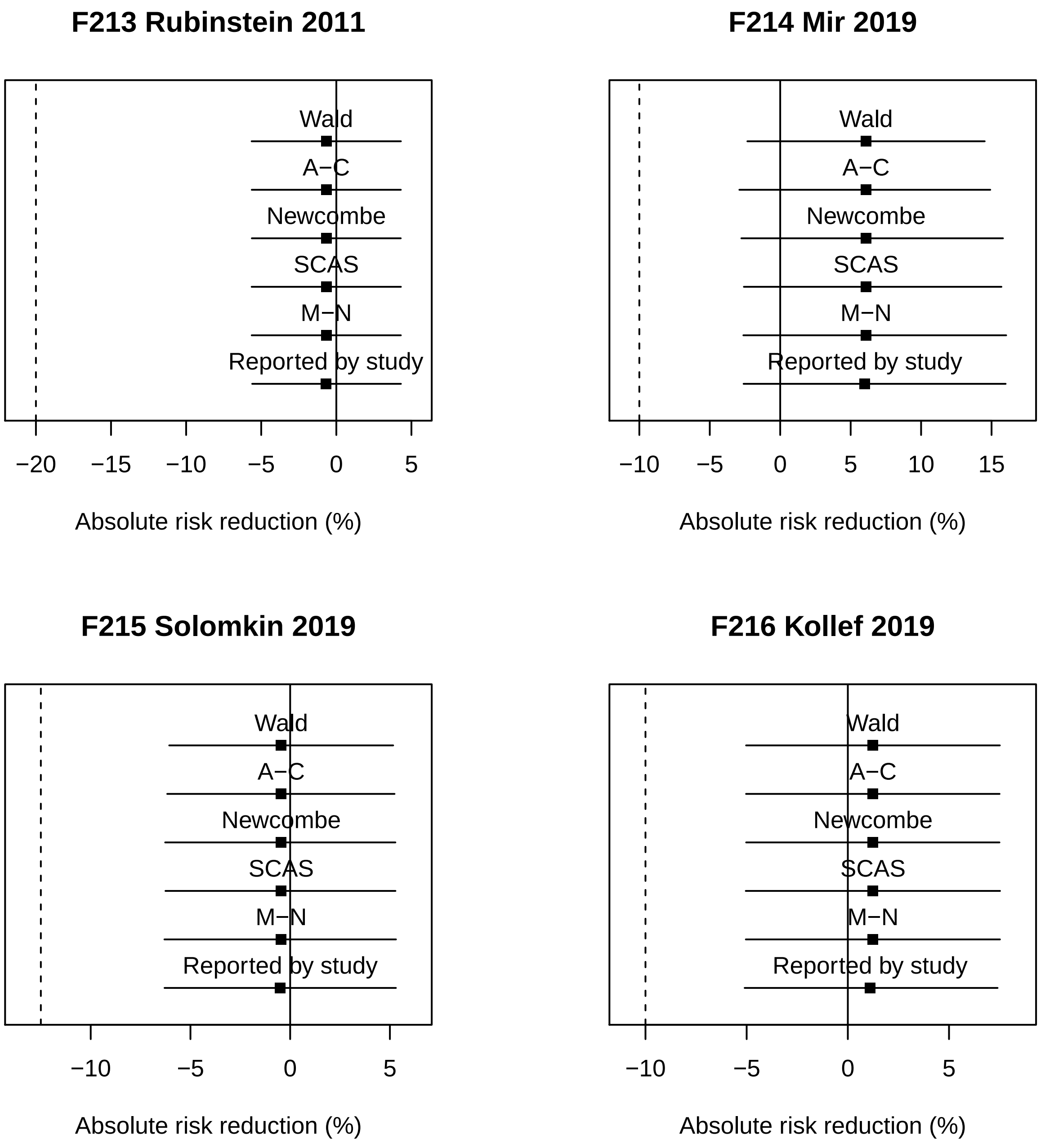
F213 Rubinstein 2011
Wald
A−C
Newcombe
SCAS
M−N
Reported by study
−20
−15
−10
−5
0
5
Absolute risk reduction (%)
F214 Mir 2019
Wald
A−C
Newcombe
SCAS
M−N
Reported by study
−10
−5
0
5
10
15
Absolute risk reduction (%)
F215 Solomkin 2019
Wald
A−C
Newcombe
SCAS
M−N
Reported by study
−10
−5
0
5
Absolute risk reduction (%)
F216 Kollef 2019
Wald
A−C
Newcombe
SCAS
M−N
Reported by study
−10
−5
0
5
Absolute risk reduction (%)

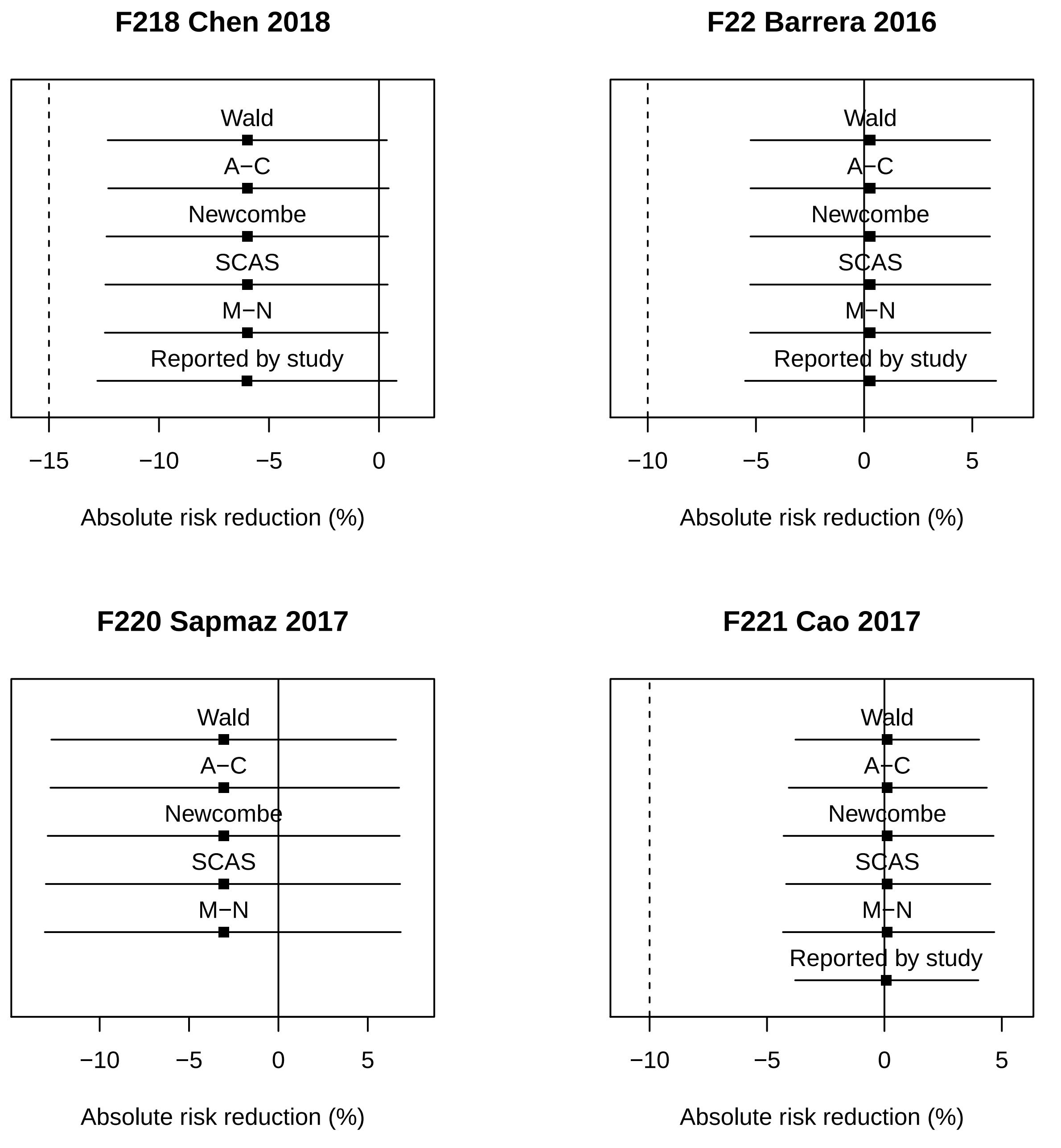
F218 Chen 2018
Wald
A−C
Newcombe
SCAS
M−N
Reported by study
−15
−10
−5
0
Absolute risk reduction (%)
F22 Barrera 2016
Wald
A−C
Newcombe
SCAS
M−N
Reported by study
−10
−5
0
5
Absolute risk reduction (%)
F220 Sapmaz 2017
Wald
A−C
Newcombe
SCAS
M−N
−10
−5
0
5
Absolute risk reduction (%)
F221 Cao 2017
Wald
A−C
Newcombe
SCAS
M−N
Reported by study
−10
−5
0
5
Absolute risk reduction (%)

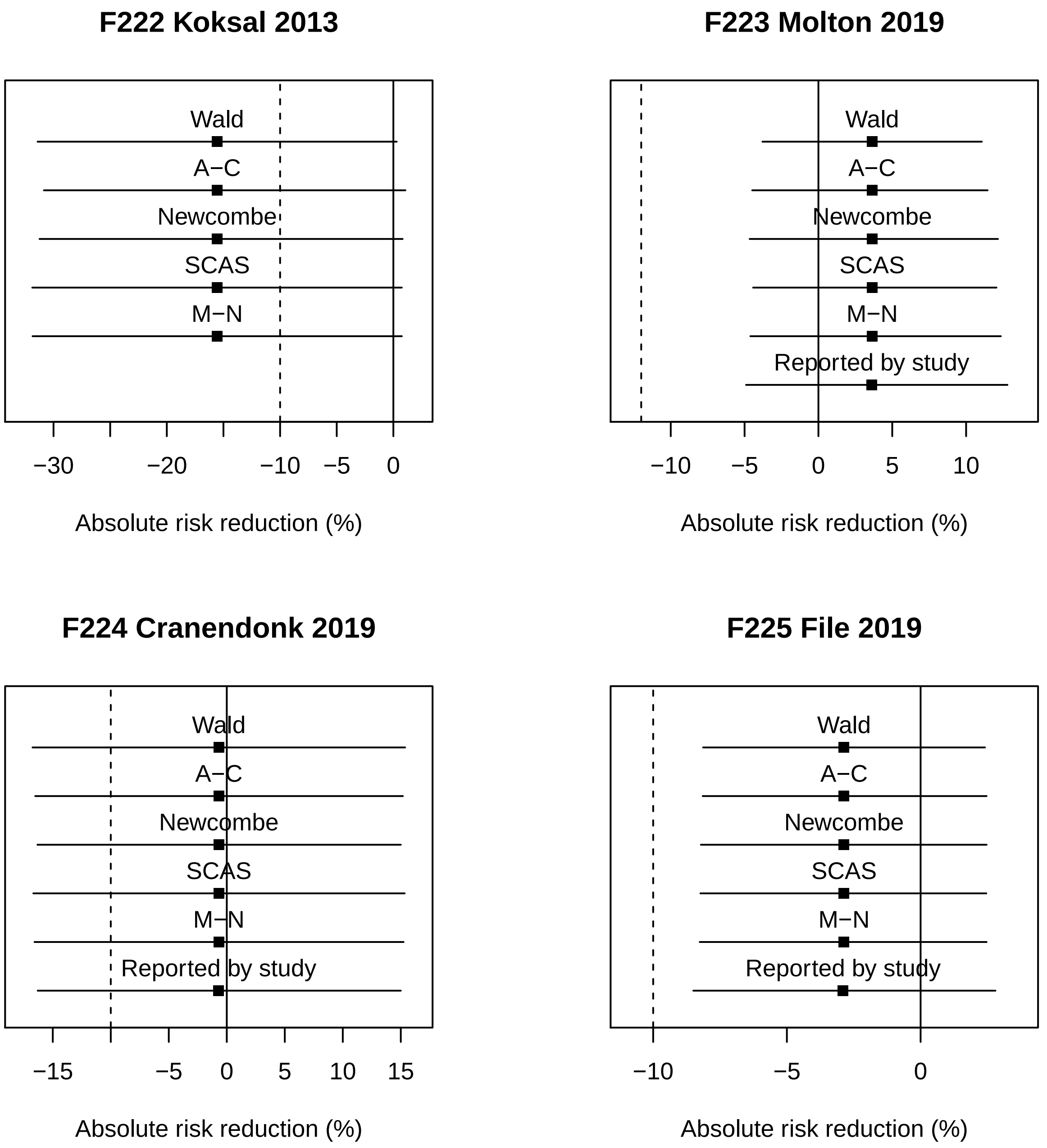

F222 Koksal 2013
Wald
A−C
Newcombe
SCAS
M−N
−30
−20
−10
−5
0
Absolute risk reduction (%)
F223 Molton 2019
Wald
A−C
Newcombe
SCAS
M−N
Reported by study
−10
−5
0
5
10
Absolute risk reduction (%)
F224 Cranendonk 2019
Wald
A−C
Newcombe
SCAS
M−N
Reported by study
−15
−5
0
5
10
15
Absolute risk reduction (%)
F225 File 2019
Wald
A−C
Newcombe
SCAS
M−N
Reported by study
−10
−5
0
Absolute risk reduction (%)

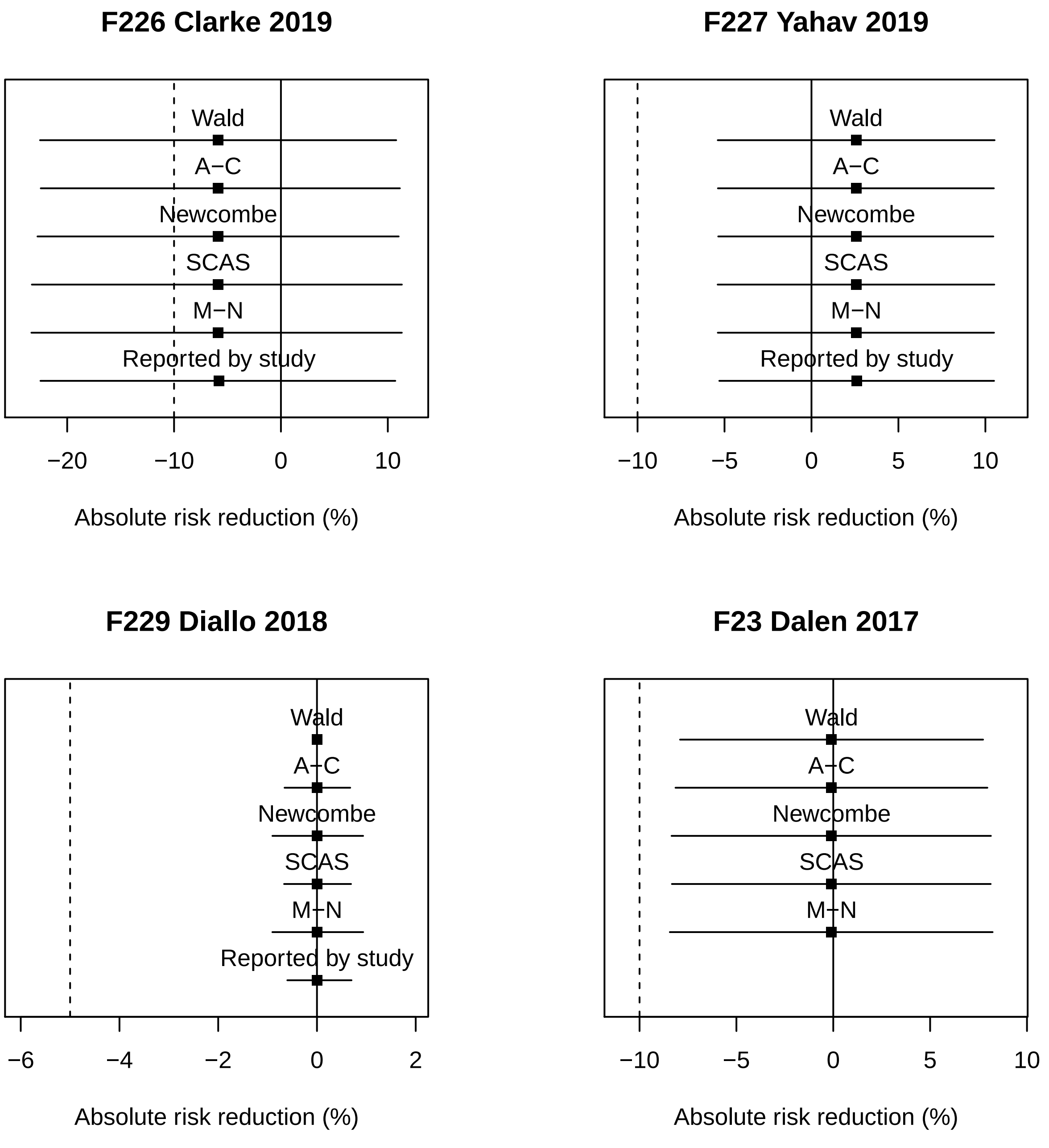

F226 Clarke 2019
Wald
A−C
Newcombe
SCAS
M−N
Reported by study
−20
−10
0
10
Absolute risk reduction (%)
F227 Yahav 2019
Wald
A−C
Newcombe
SCAS
M−N
Reported by study
−10
−5
0
5
10
Absolute risk reduction (%)
F229 Diallo 2018
Wald
A−C
Newcombe
SCAS
M−N
Reported by study
−6
−4
−2
0
2
Absolute risk reduction (%)
F23 Dalen 2017
Wald
A−C
Newcombe
SCAS
M−N
−10
−5
0
5
10
Absolute risk reduction (%)

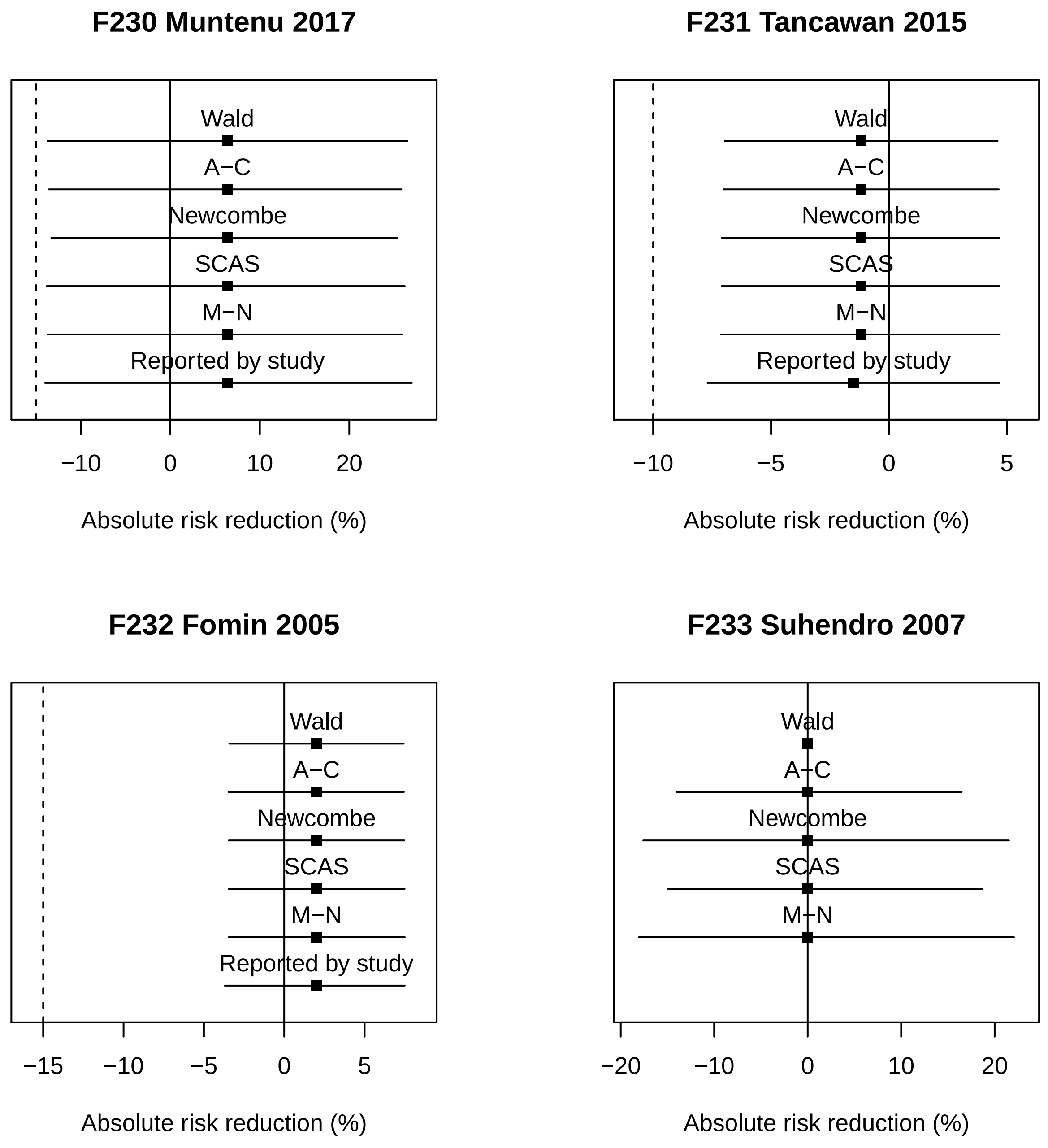
F230 Muntenu 2017
Wald
A−C
Newcombe
SCAS
M−N
Reported by study
−10
0
10
20
Absolute risk reduction (%)
F231 Tancawan 2015
Wald
A−C
Newcombe
SCAS
M−N
Reported by study
−10
−5
0
5
Absolute risk reduction (%)
F232 Fomin 2005
Wald
A−C
Newcombe
SCAS
M−N
Reported by study
−15
−10
−5
0
5
Absolute risk reduction (%)
F233 Suhendro 2007
Wald
A−C
Newcombe
SCAS
M−N
−20
−10
0
10
20
Absolute risk reduction (%)

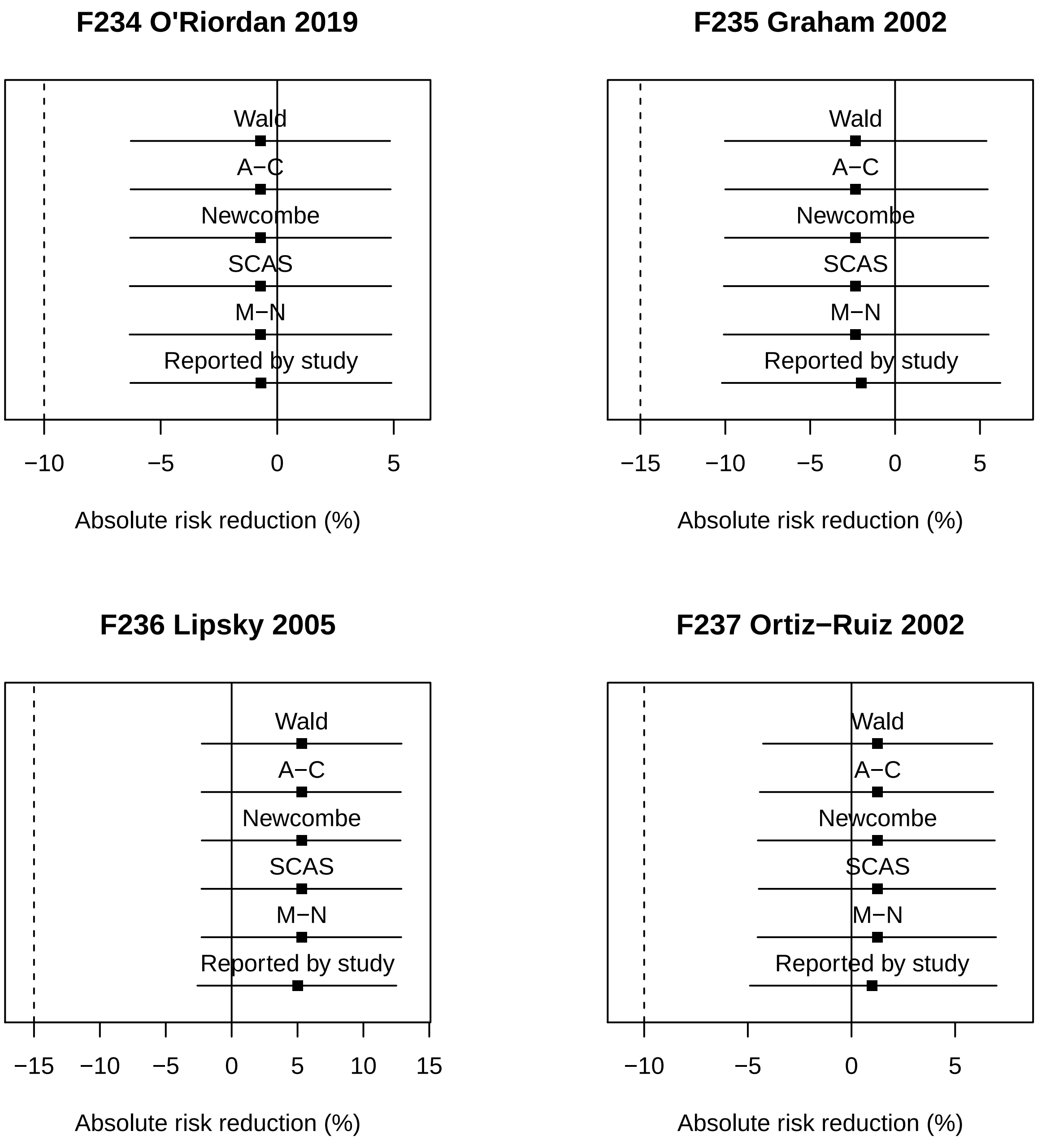

F234 O'Riordan 2019
Wald
A−C
Newcombe
SCAS
M−N
Reported by study
−10
−5
0
5
Absolute risk reduction (%)
F235 Graham 2002
Wald
A−C
Newcombe
SCAS
M−N
Reported by study
−15
−10
−5
0
5
Absolute risk reduction (%)
F236 Lipsky 2005
Wald
A−C
Newcombe
SCAS
M−N
Reported by study
−15
−10
−5
0
5
10
15
Absolute risk reduction (%)
F237 Ortiz−Ruiz 2002
Wald
A−C
Newcombe
SCAS
M−N
Reported by study
−10
−5
0
5
Absolute risk reduction (%)

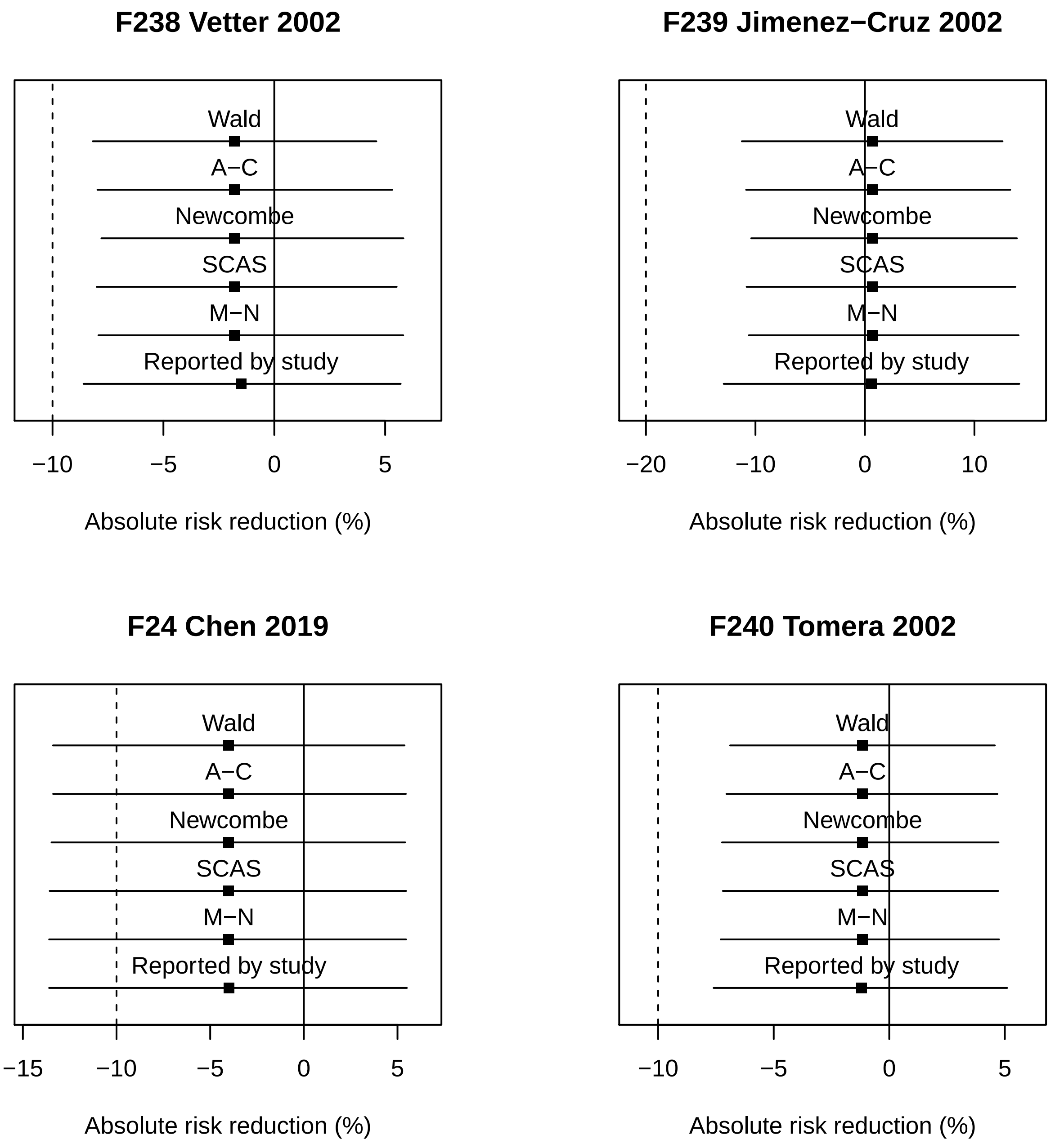
F238 Vetter 2002
Wald
A−C
Newcombe
SCAS
M−N
Reported by study
−10
−5
0
5
Absolute risk reduction (%)
F239 Jimenez−Cruz 2002
Wald
A−C
Newcombe
SCAS
M−N
Reported by study
−20
−10
0
10
Absolute risk reduction (%)
F24 Chen 2019
Wald
A−C
Newcombe
SCAS
M−N
Reported by study
−15
−10
−5
0
5
Absolute risk reduction (%)
F240 Tomera 2002
Wald
A−C
Newcombe
SCAS
M−N
Reported by study
−10
−5
0
5
Absolute risk reduction (%)

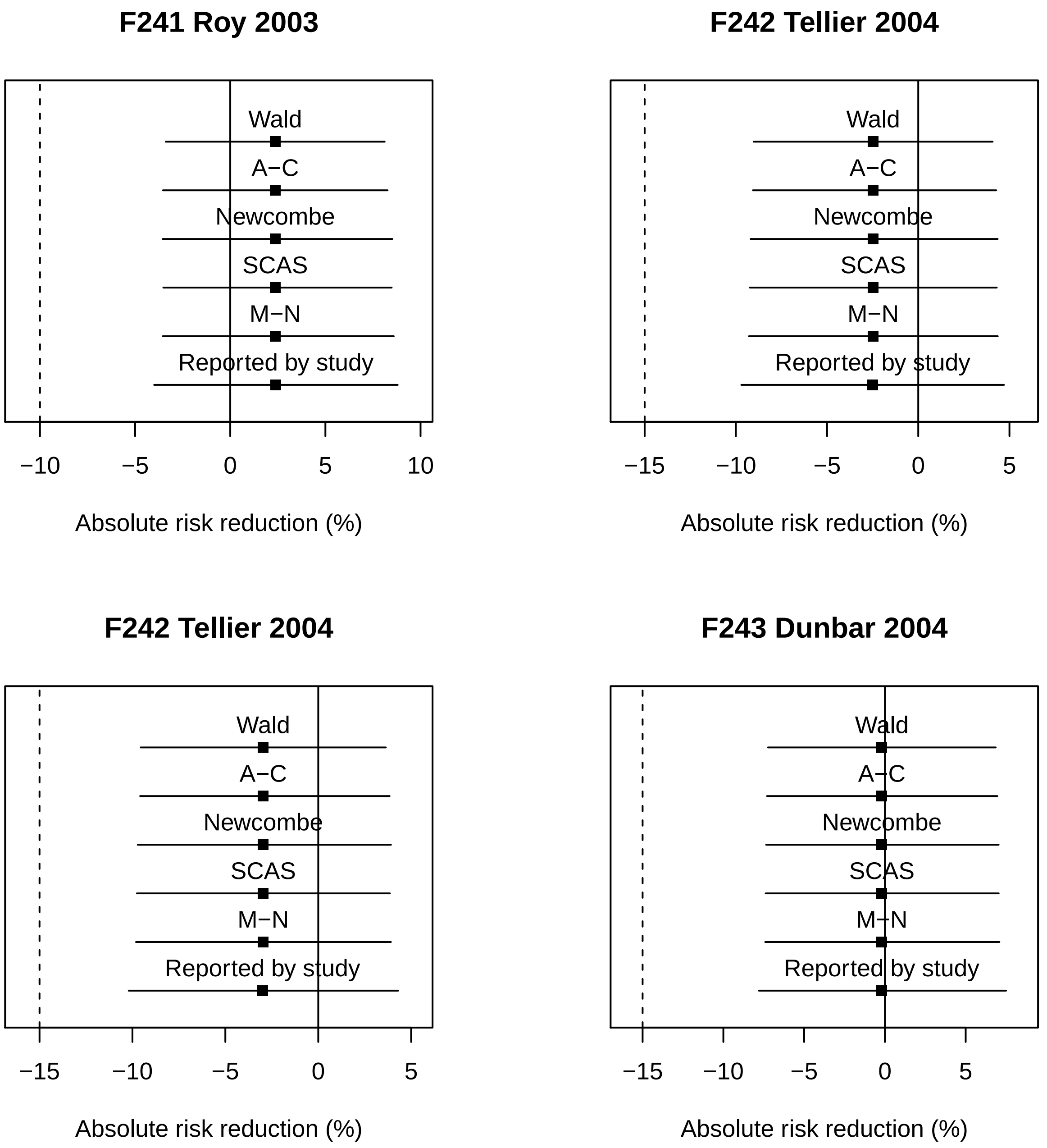
F241 Roy 2003
Wald
A−C
Newcombe
SCAS
M−N
Reported by study
−10
−5
0
5
10
Absolute risk reduction (%)
F242 Tellier 2004
Wald
A−C
Newcombe
SCAS
M−N
Reported by study
−15
−10
−5
0
5
Absolute risk reduction (%)
F242 Tellier 2004
Wald
A−C
Newcombe
SCAS
M−N
Reported by study
−15
−10
−5
0
5
Absolute risk reduction (%)
F243 Dunbar 2004
Wald
A−C
Newcombe
SCAS
M−N
Reported by study
−15
−10
−5
0
5
Absolute risk reduction (%)

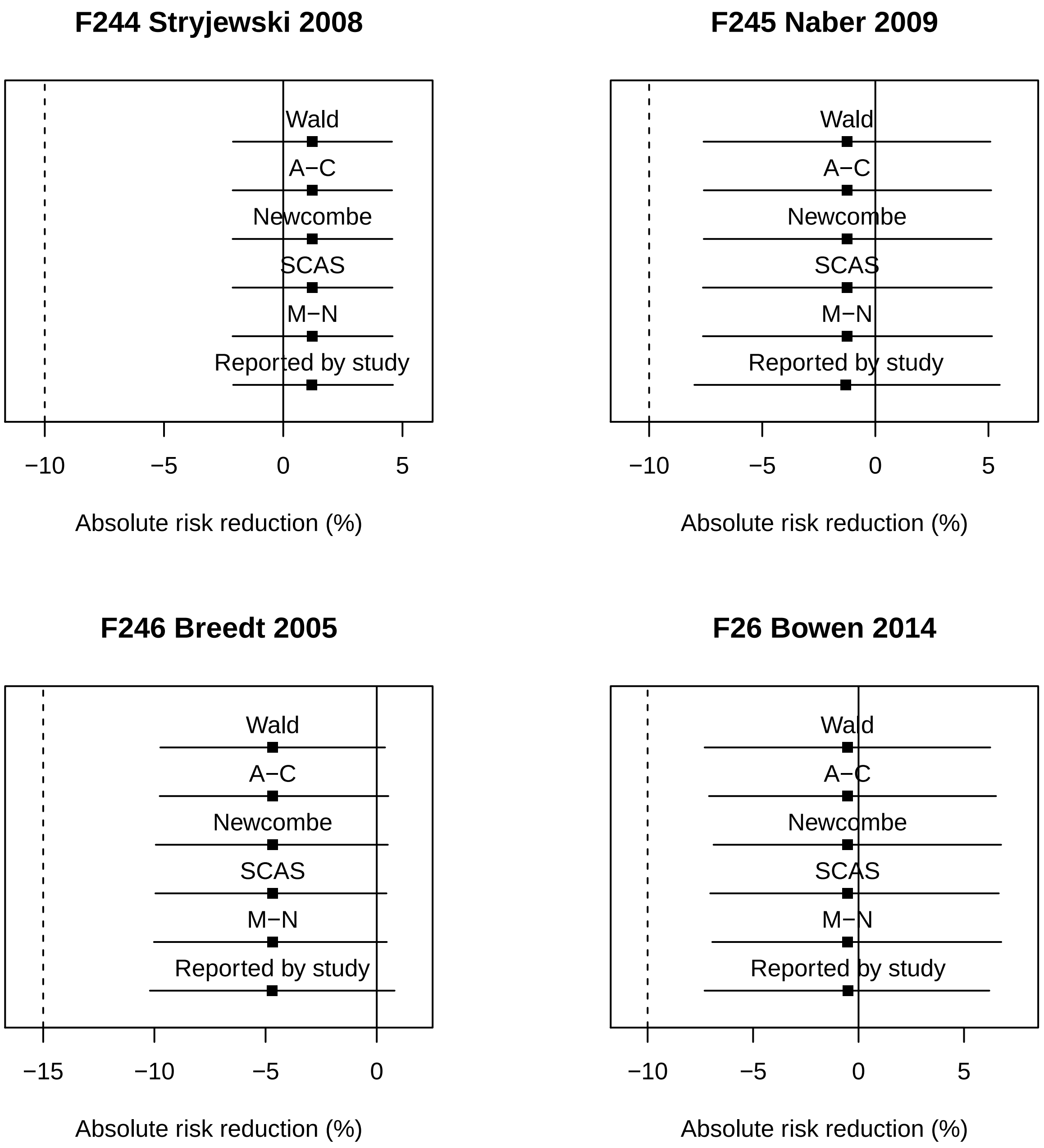
F244 Stryjewski 2008
Wald
A−C
Newcombe
SCAS
M−N
Reported by study
−10
−5
0
5
Absolute risk reduction (%)
F245 Naber 2009
Wald
A−C
Newcombe
SCAS
M−N
Reported by study
−10
−5
0
5
Absolute risk reduction (%)
F246 Breedt 2005
Wald
A−C
Newcombe
SCAS
M−N
Reported by study
−15
−10
−5
0
Absolute risk reduction (%)
F26 Bowen 2014
Wald
A−C
Newcombe
SCAS
M−N
Reported by study
−10
−5
0
5
Absolute risk reduction (%)

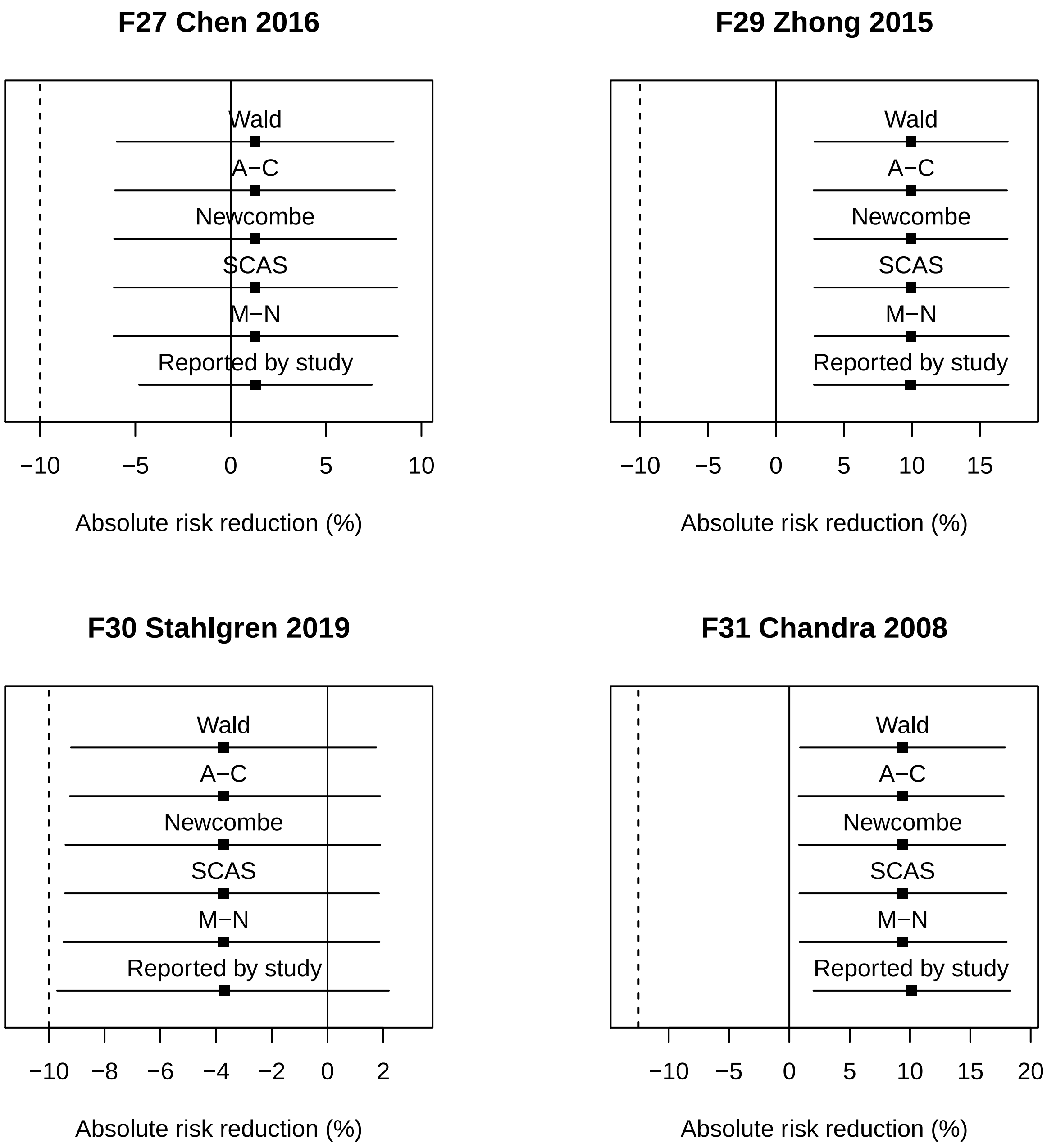
F27 Chen 2016
Wald
A−C
Newcombe
SCAS
M−N
Reported by study
−10
−5
0
5
10
Absolute risk reduction (%)
F29 Zhong 2015
Wald
A−C
Newcombe
SCAS
M−N
Reported by study
−10
−5
0
5
10
15
Absolute risk reduction (%)
F30 Stahlgren 2019
Wald
A−C
Newcombe
SCAS
M−N
Reported by study
−10
−8
−6
−4
−2
0
2
Absolute risk reduction (%)
F31 Chandra 2008
Wald
A−C
Newcombe
SCAS
M−N
Reported by study
−10
−5
0
5
10
15
20
Absolute risk reduction (%)

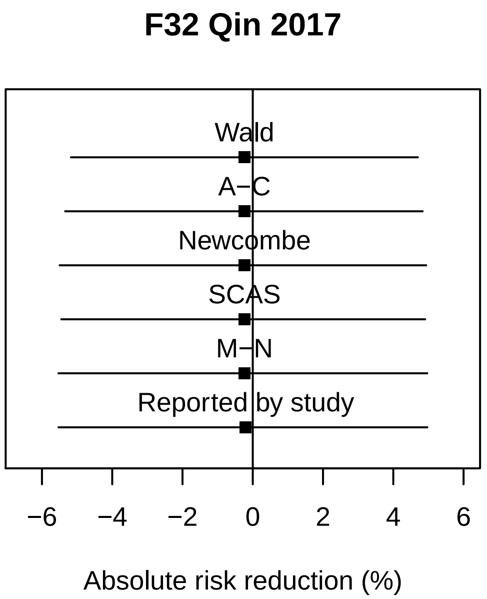
F32 Qin 2017
Wald
A−C
Newcombe
SCAS
M−N
Reported by study
−6
−4
−2
0
2
4
6
Absolute risk reduction (%)

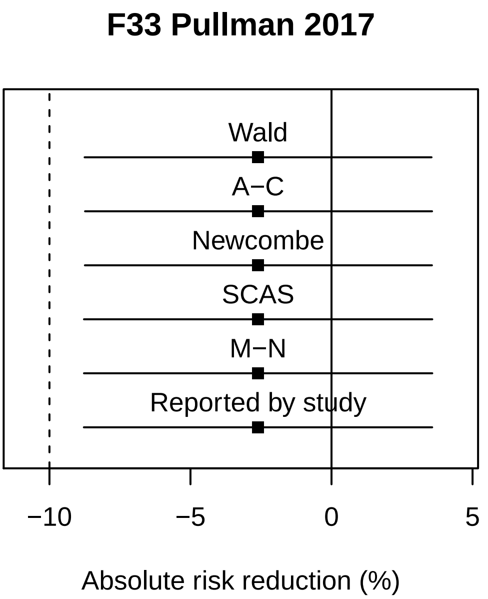
F33 Pullman 2017
Wald
A−C
Newcombe
SCAS
M−N
Reported by study
−10
−5
0
5
Absolute risk reduction (%)

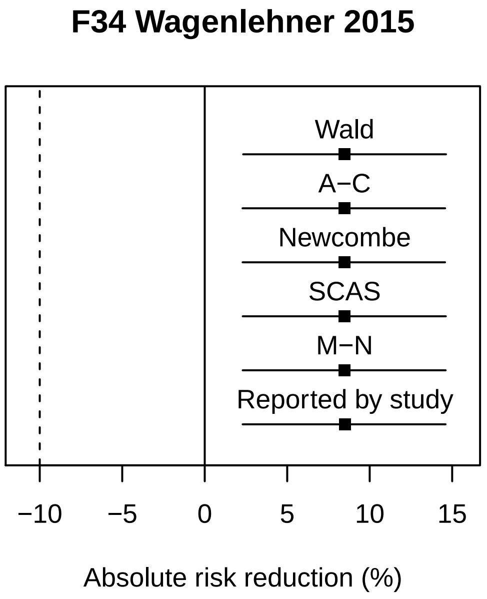
F34 Wagenlehner 2015
Wald
A−C
Newcombe
SCAS
M−N
Reported by study
−10
−5
0
5
10
15
Absolute risk reduction (%)

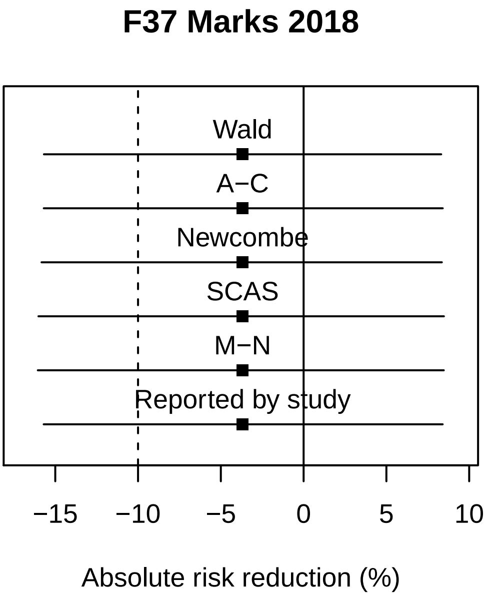
F37 Marks 2018
Wald
A−C
Newcombe
SCAS
M−N
Reported by study
−15
−10
−5
0
5
10
Absolute risk reduction (%)

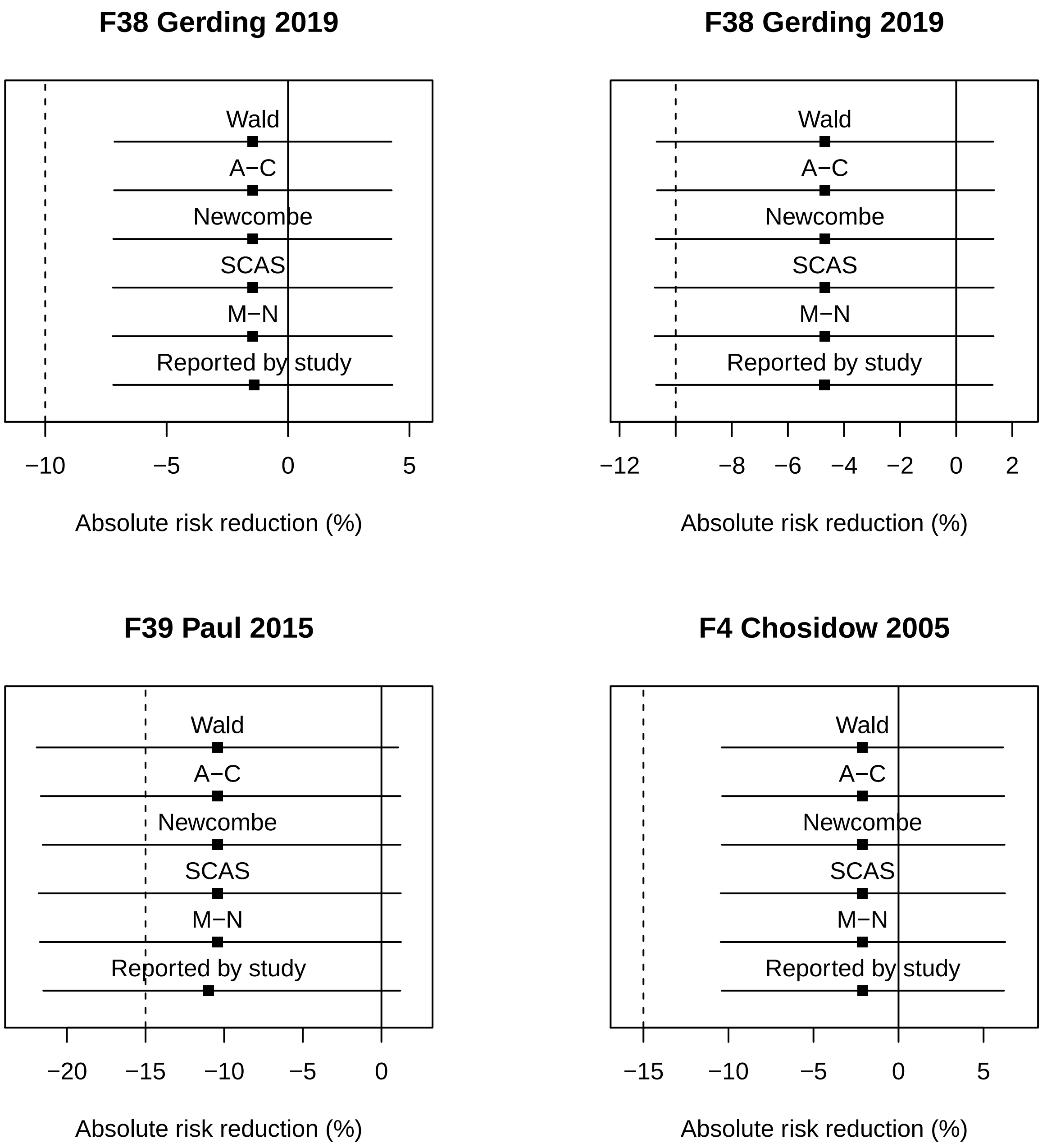
F38 Gerding 2019
Wald
A−C
Newcombe
SCAS
M−N
Reported by study
−10
−5
0
5
Absolute risk reduction (%)
F38 Gerding 2019
Wald
A−C
Newcombe
SCAS
M−N
Reported by study
−12
−8
−6
−4
−2
0
2
Absolute risk reduction (%)
F39 Paul 2015
Wald
A−C
Newcombe
SCAS
M−N
Reported by study
−20
−15
−10
−5
0
Absolute risk reduction (%)
F4 Chosidow 2005
Wald
A−C
Newcombe
SCAS
M−N
Reported by study
−15
−10
−5
0
5
Absolute risk reduction (%)

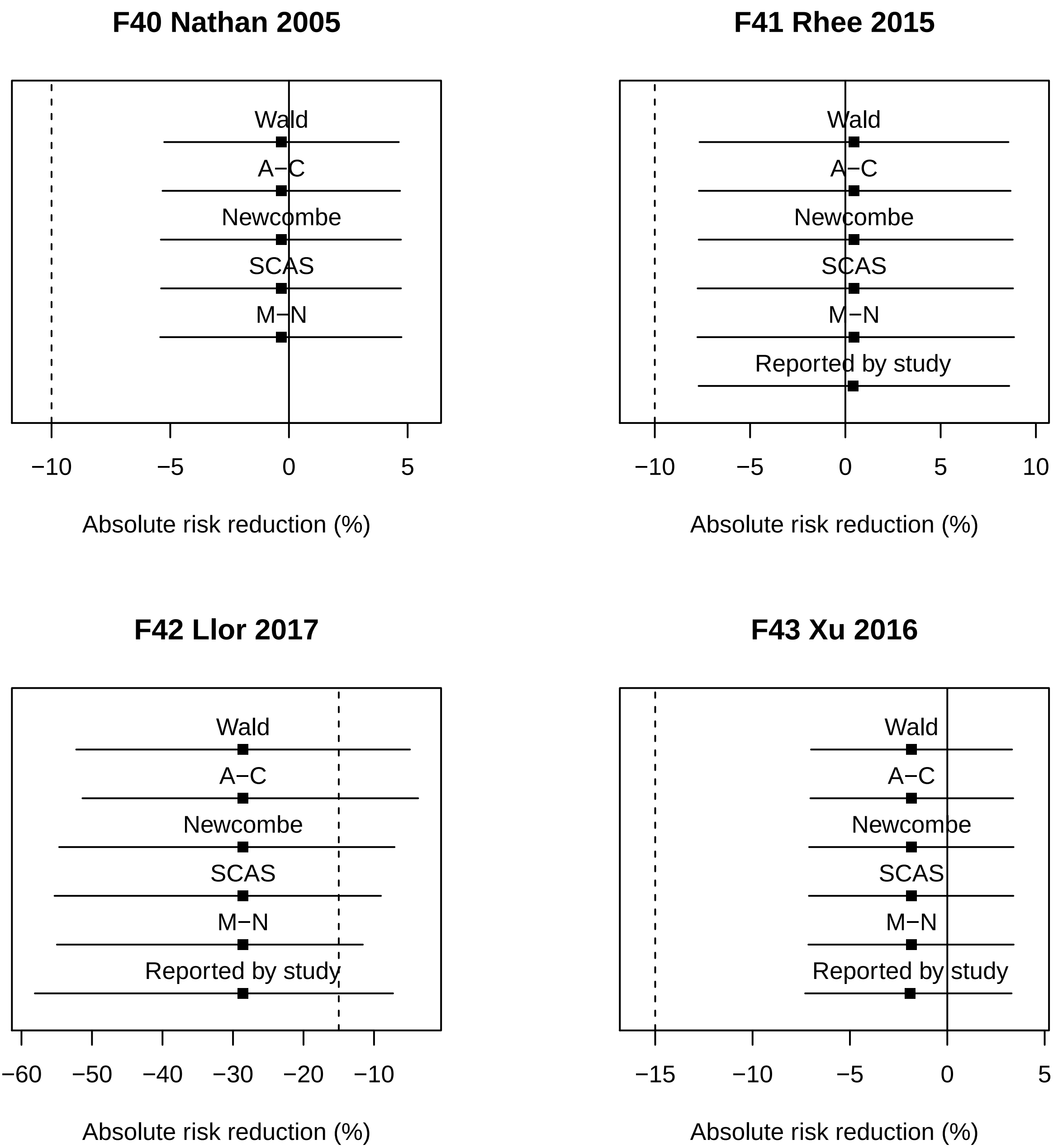
F40 Nathan 2005
Wald
A−C
Newcombe
SCAS
M−N
−10
−5
0
5
Absolute risk reduction (%)
F41 Rhee 2015
Wald
A−C
Newcombe
SCAS
M−N
Reported by study
−10
−5
0
5
10
Absolute risk reduction (%)
F42 Llor 2017
Wald
A−C
Newcombe
SCAS
M−N
Reported by study
−60
−50
−40
−30
−20
−10
Absolute risk reduction (%)
F43 Xu 2016
Wald
A−C
Newcombe
SCAS
M−N
Reported by study
−15
−10
−5
0
5
Absolute risk reduction (%)

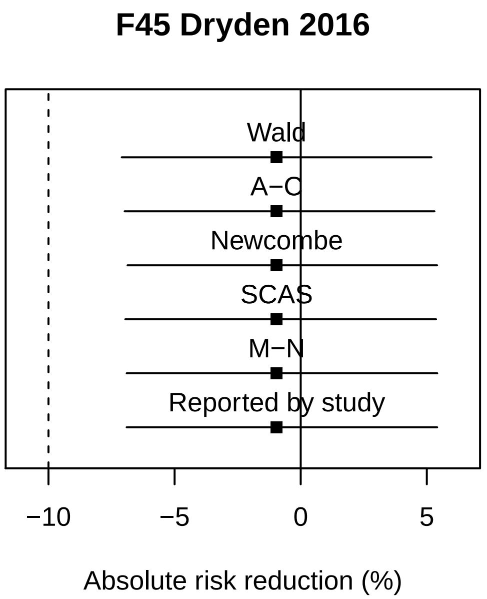
F45 Dryden 2016
Wald
A−C
Newcombe
SCAS
M−N
Reported by study
−10
−5
0
5
Absolute risk reduction (%)

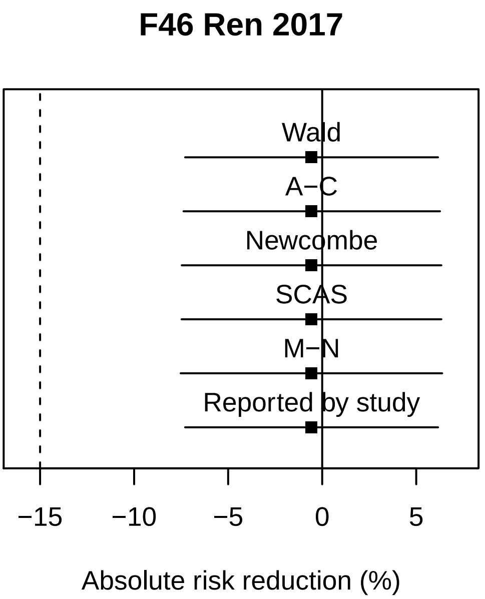
F46 Ren 2017
Wald
A−C
Newcombe
SCAS
M−N
Reported by study
−15
−10
−5
0
5
Absolute risk reduction (%)

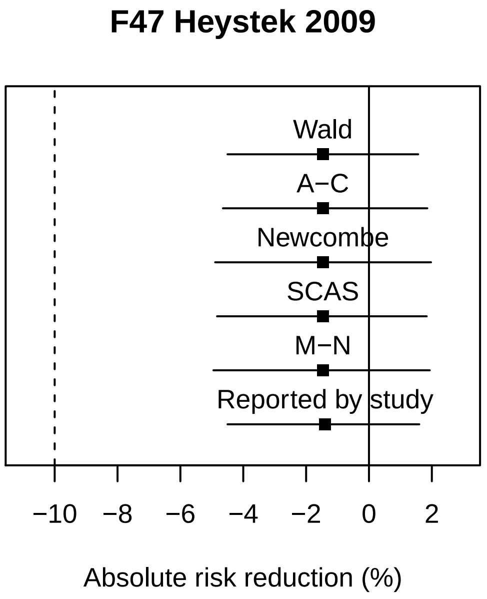
F47 Heystek 2009
Wald
A−C
Newcombe
SCAS
M−N
Reported by study
−10
−8
−6
−4
−2
0
2
Absolute risk reduction (%)

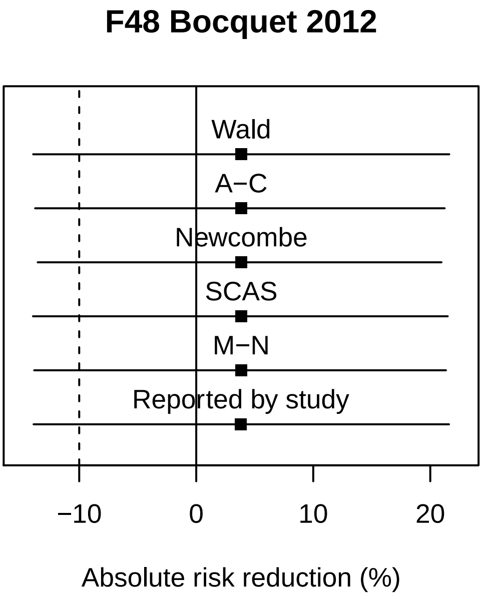
F48 Bocquet 2012
Wald
A−C
Newcombe
SCAS
M−N
Reported by study
−10
0
10
20
Absolute risk reduction (%)

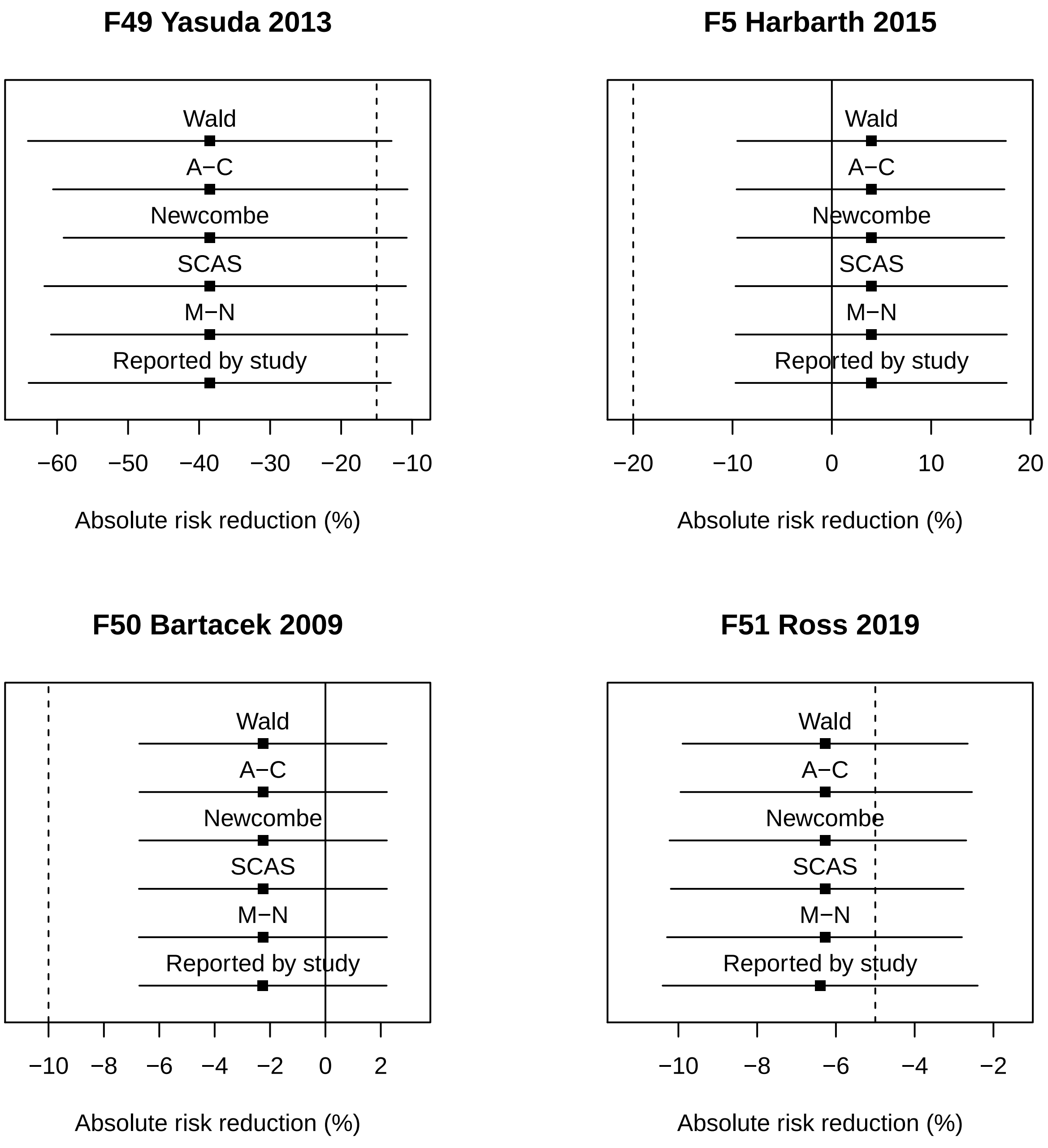
F49 Yasuda 2013
Wald
A−C
Newcombe
SCAS
M−N
Reported by study
−60
−50
−40
−30
−20
−10
Absolute risk reduction (%)
F5 Harbarth 2015
Wald
A−C
Newcombe
SCAS
M−N
Reported by study
−20
−10
0
10
20
Absolute risk reduction (%)
F50 Bartacek 2009
Wald
A−C
Newcombe
SCAS
M−N
Reported by study
−10
−8
−6
−4
−2
0
2
Absolute risk reduction (%)
F51 Ross 2019
Wald
A−C
Newcombe
SCAS
M−N
Reported by study
−10
−8
−6
−4
−2
Absolute risk reduction (%)

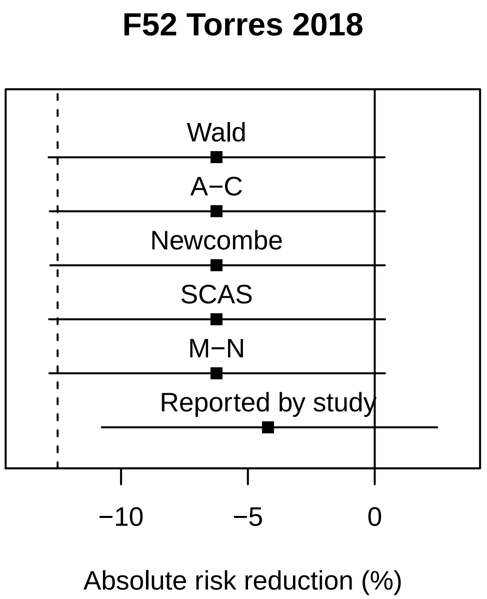
F52 Torres 2018
Wald
A−C
Newcombe
SCAS
M−N
Reported by study
−10
−5
0
Absolute risk reduction (%)

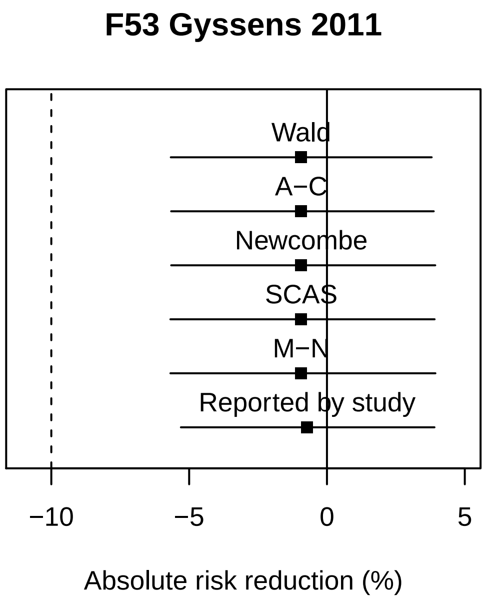
F53 Gyssens 2011
Wald
A−C
Newcombe
SCAS
M−N
Reported by study
−10
−5
0
5
Absolute risk reduction (%)

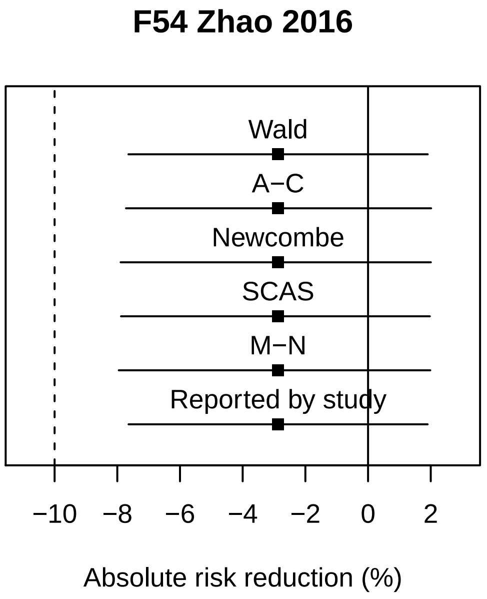
F54 Zhao 2016
Wald
A−C
Newcombe
SCAS
M−N
Reported by study
−10
−8
−6
−4
−2
0
2
Absolute risk reduction (%)

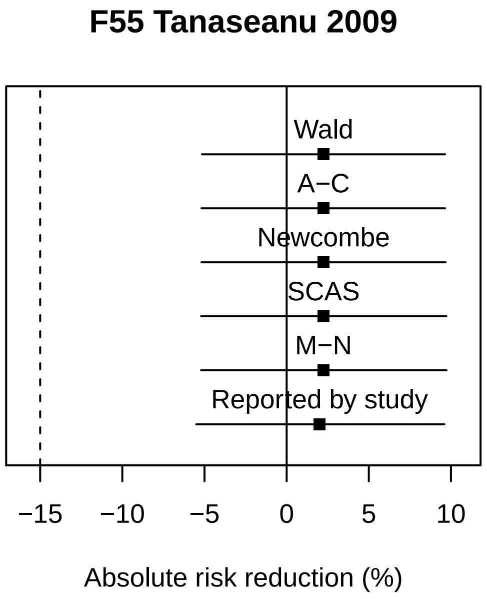
F55 Tanaseanu 2009
Wald
A−C
Newcombe
SCAS
M−N
Reported by study
−15
−10
−5
0
5
10
Absolute risk reduction (%)

F56 Towfigh 2009

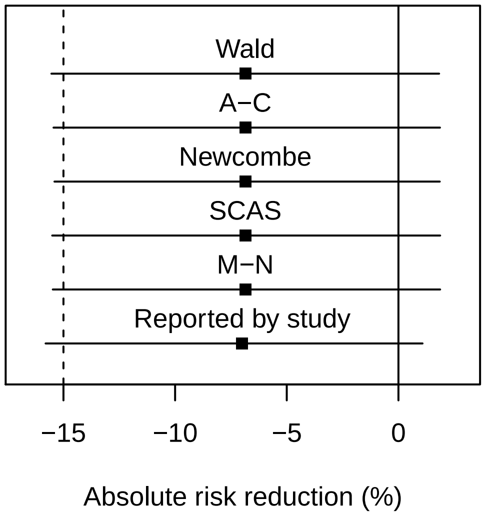

F57 Yakovlev 2006

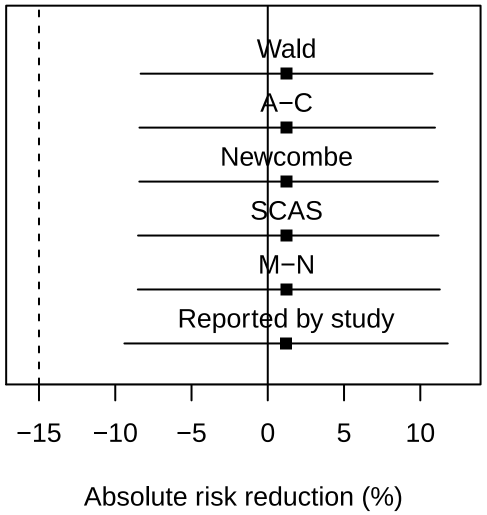

F58 Tazuma 2015

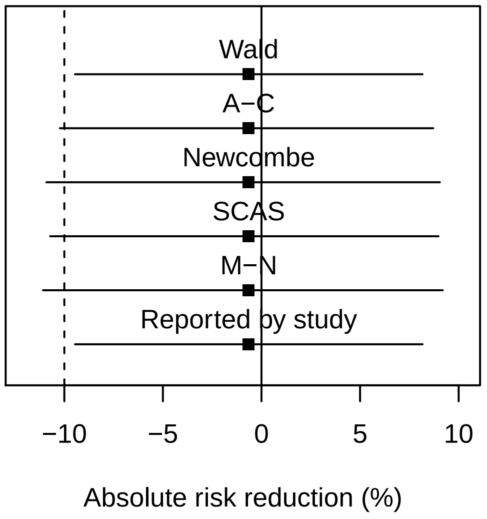

F59 Nakane 2015

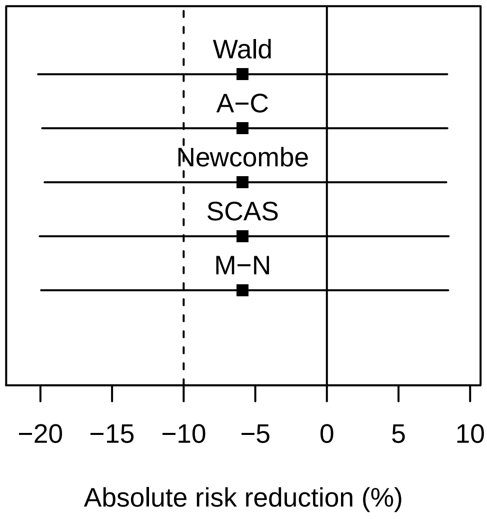

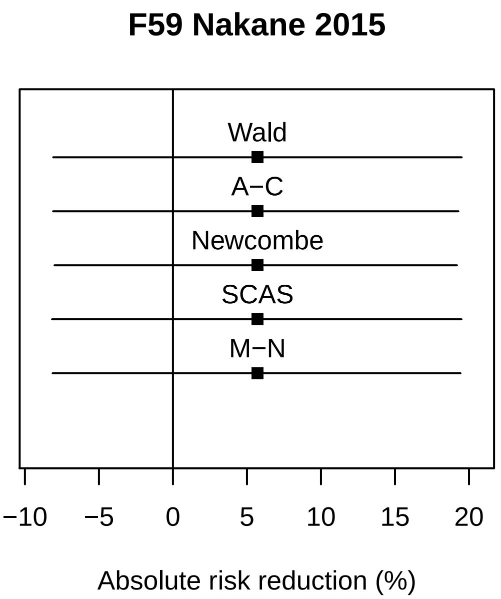
F59 Nakane 2015
Wald
A−C
Newcombe
SCAS
M−N
−10 −5 0 5 10 15 20
Absolute risk reduction (%)

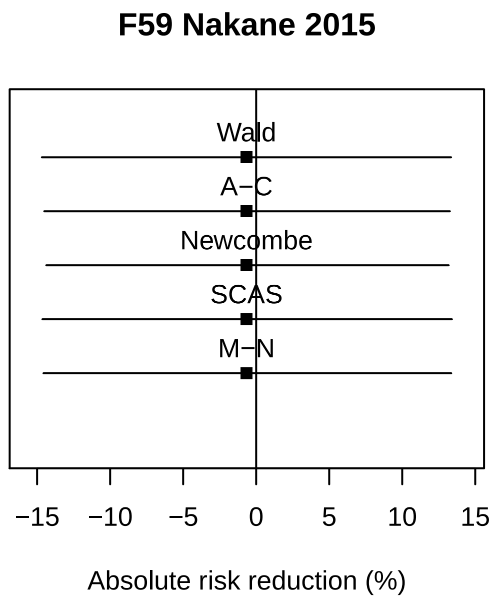
F59 Nakane 2015
Wald
A−C
Newcombe
SCAS
M−N
−15 −10 −5 0 5 10 15
Absolute risk reduction (%)

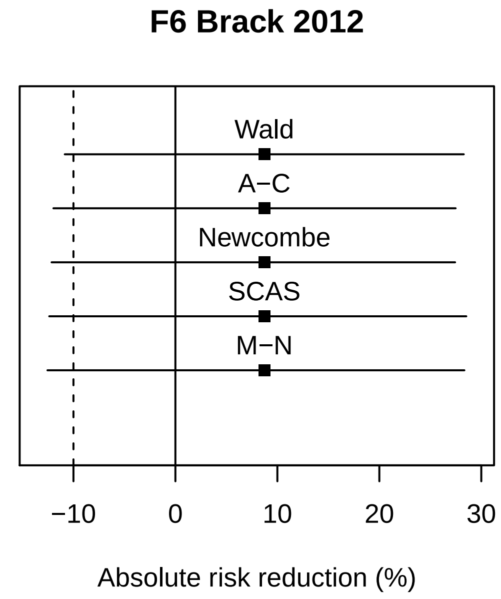
F6 Brack 2012
Wald
A−C
Newcombe
SCAS
M−N
−10 0 10 20 30
Absolute risk reduction (%)

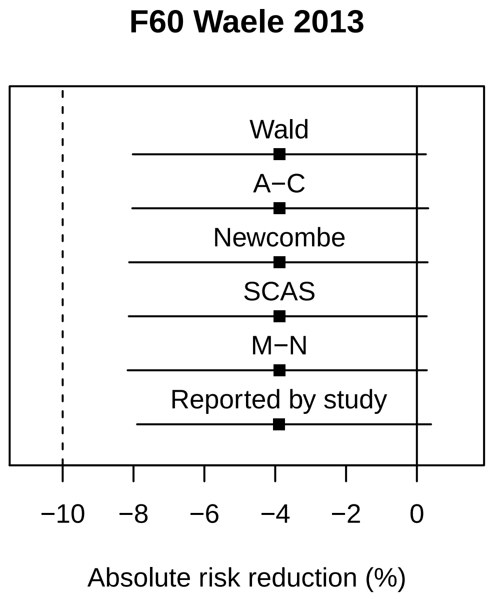
F60 Waele 2013
Wald
A−C
Newcombe
SCAS
M−N
Reported by study
−10 −8 −6 −4 −2 0
Absolute risk reduction (%)

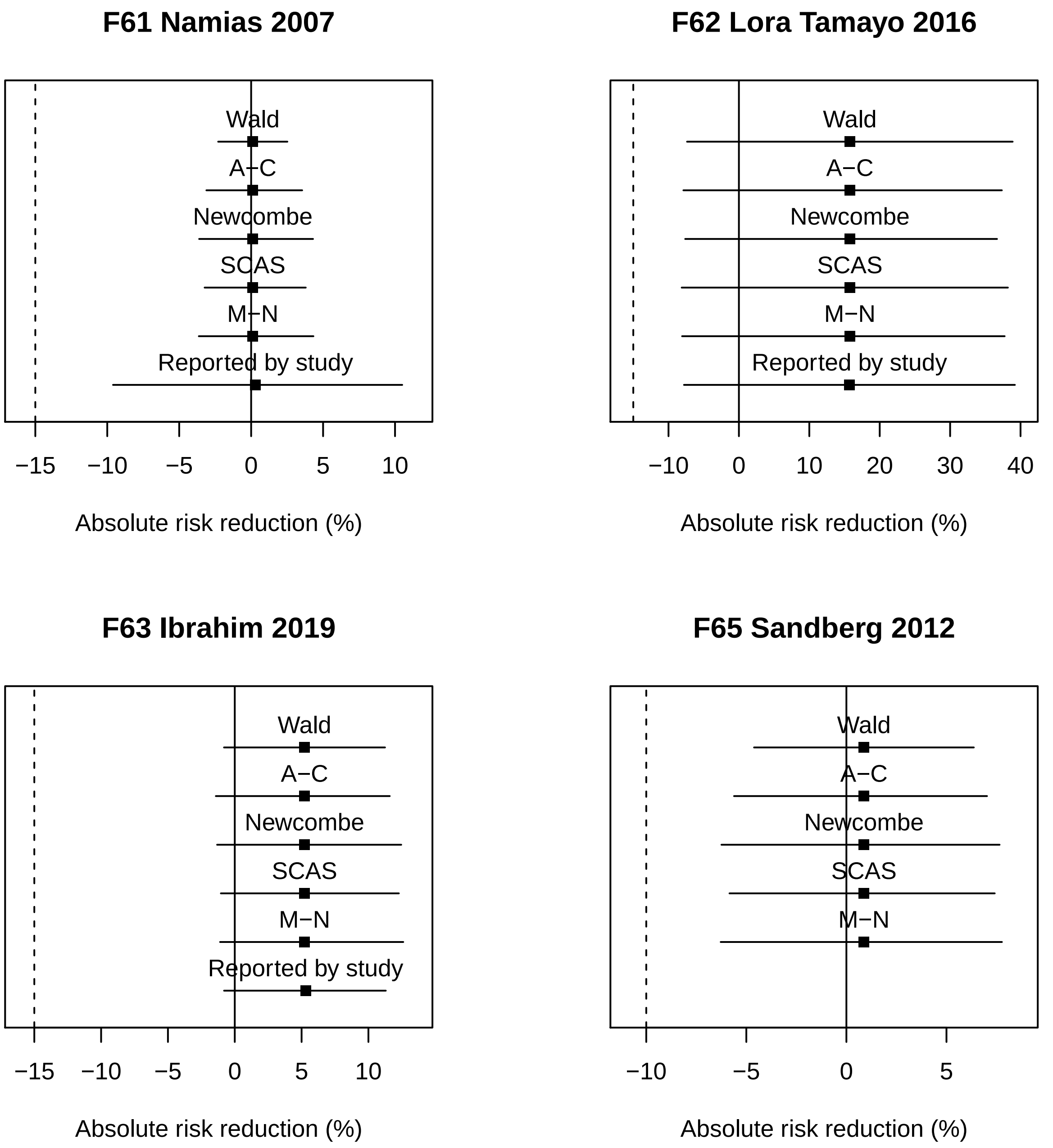
F61 Namias 2007
Wald
A−C
Newcombe
SCAS
M−N
Reported by study
−15
−10
−5
0
5
10
Absolute risk reduction (%)
F62 Lora Tamayo 2016
Wald
A−C
Newcombe
SCAS
M−N
Reported by study
−10
0
10
20
30
40
Absolute risk reduction (%)
F63 Ibrahim 2019
Wald
A−C
Newcombe
SCAS
M−N
Reported by study
−15
−10
−5
0
5
10
Absolute risk reduction (%)
F65 Sandberg 2012
Wald
A−C
Newcombe
SCAS
M−N
−10
−5
0
5
Absolute risk reduction (%)

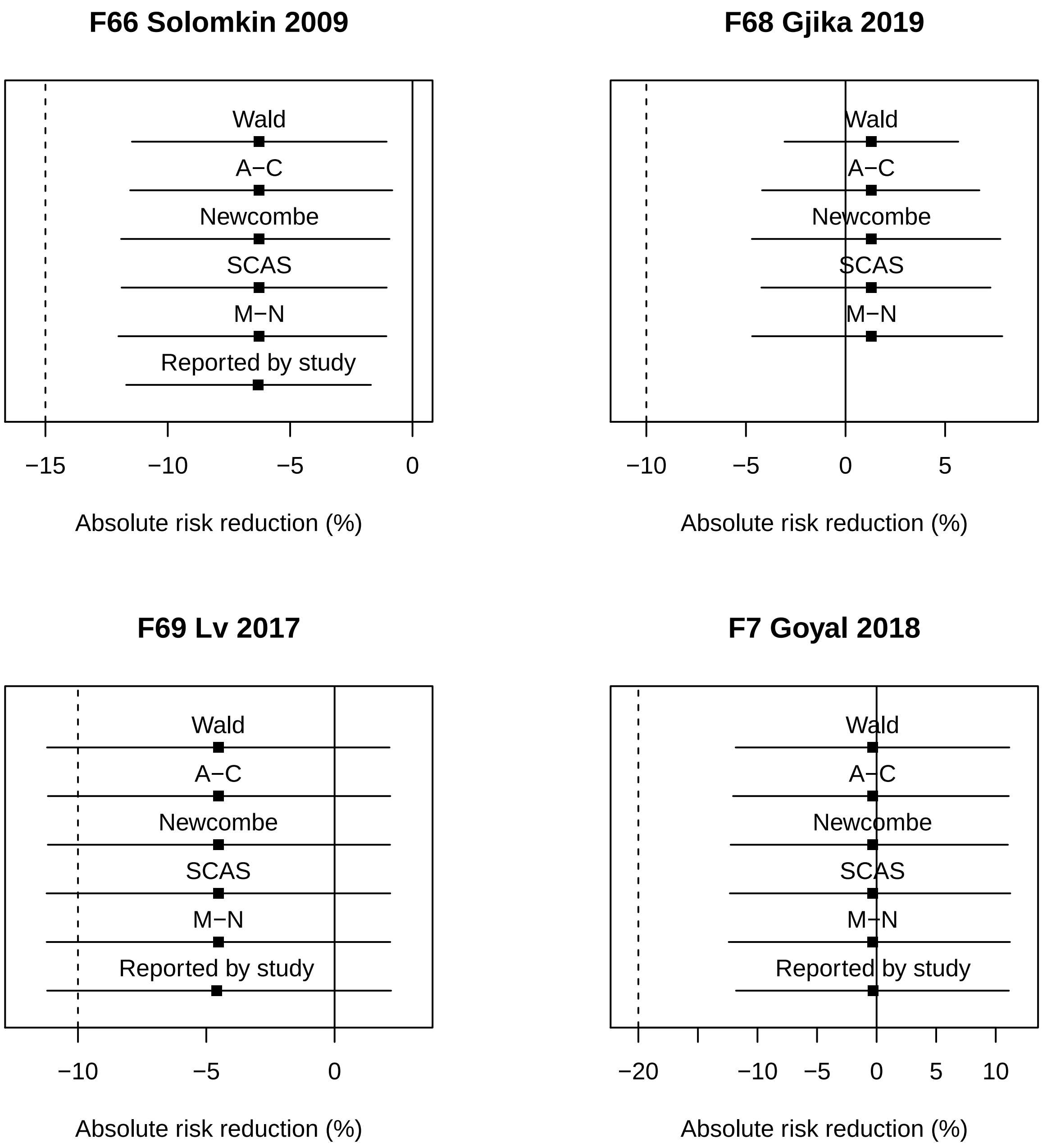
F66 Solomkin 2009
Wald
A−C
Newcombe
SCAS
M−N
Reported by study
−15
−10
−5
0
Absolute risk reduction (%)
F68 Gjika 2019
Wald
A−C
Newcombe
SCAS
M−N
−10
−5
0
5
Absolute risk reduction (%)
F69 Lv 2017
Wald
A−C
Newcombe
SCAS
M−N
Reported by study
−10
−5
0
Absolute risk reduction (%)
F7 Goyal 2018
Wald
A−C
Newcombe
SCAS
M−N
Reported by study
−20
−10
−5
0
5
10
Absolute risk reduction (%)

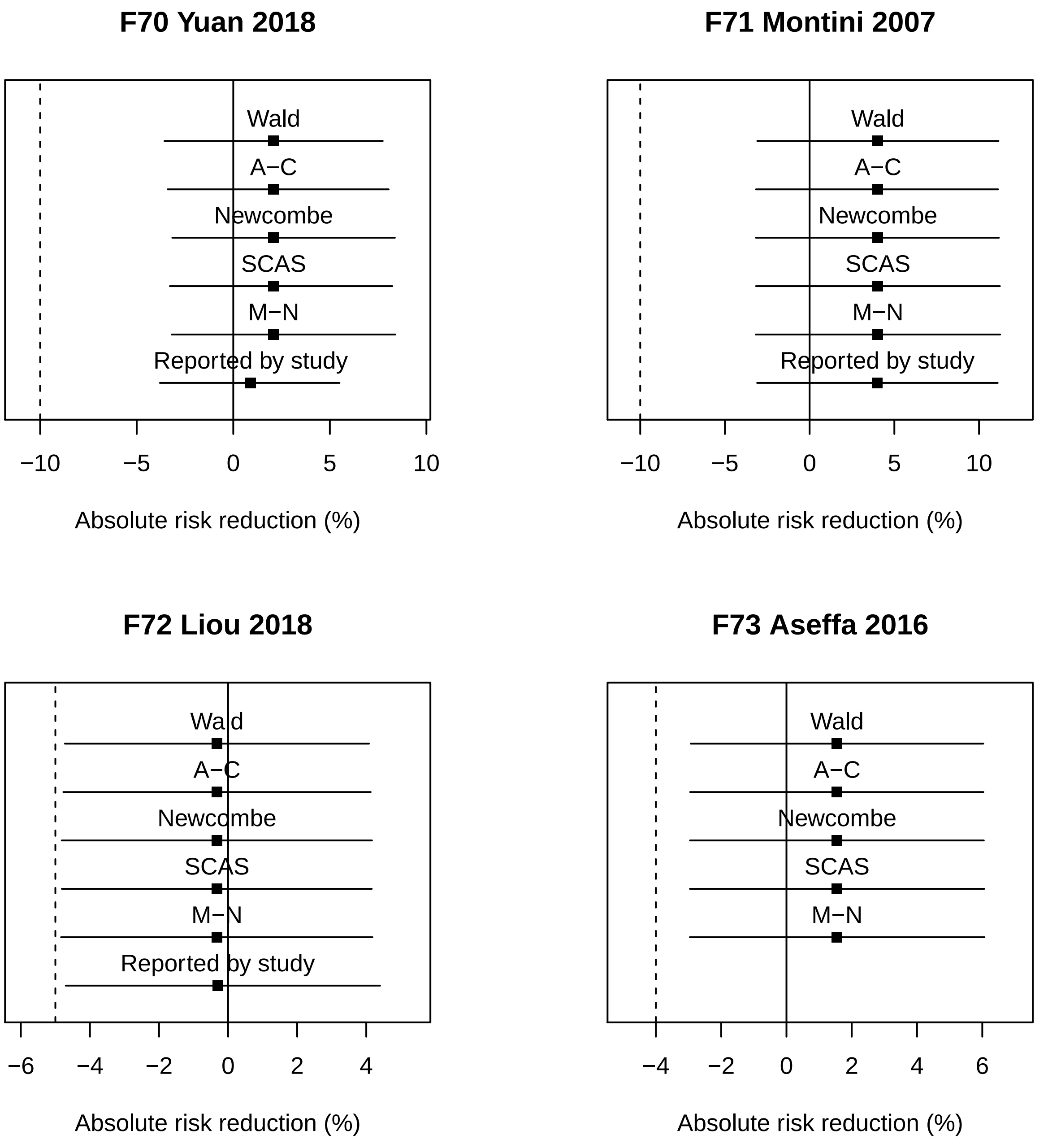

F70 Yuan 2018
Wald
A−C
Newcombe
SCAS
M−N
Reported by study
−10
−5
0
5
10
Absolute risk reduction (%)
F71 Montini 2007
Wald
A−C
Newcombe
SCAS
M−N
Reported by study
−10
−5
0
5
10
Absolute risk reduction (%)
F72 Liou 2018
Wald
A−C
Newcombe
SCAS
M−N
Reported by study
−6
−4
−2
0
2
4
Absolute risk reduction (%)
F73 Aseffa 2016
Wald
A−C
Newcombe
SCAS
M−N
−4
−2
0
2
4
6
Absolute risk reduction (%)

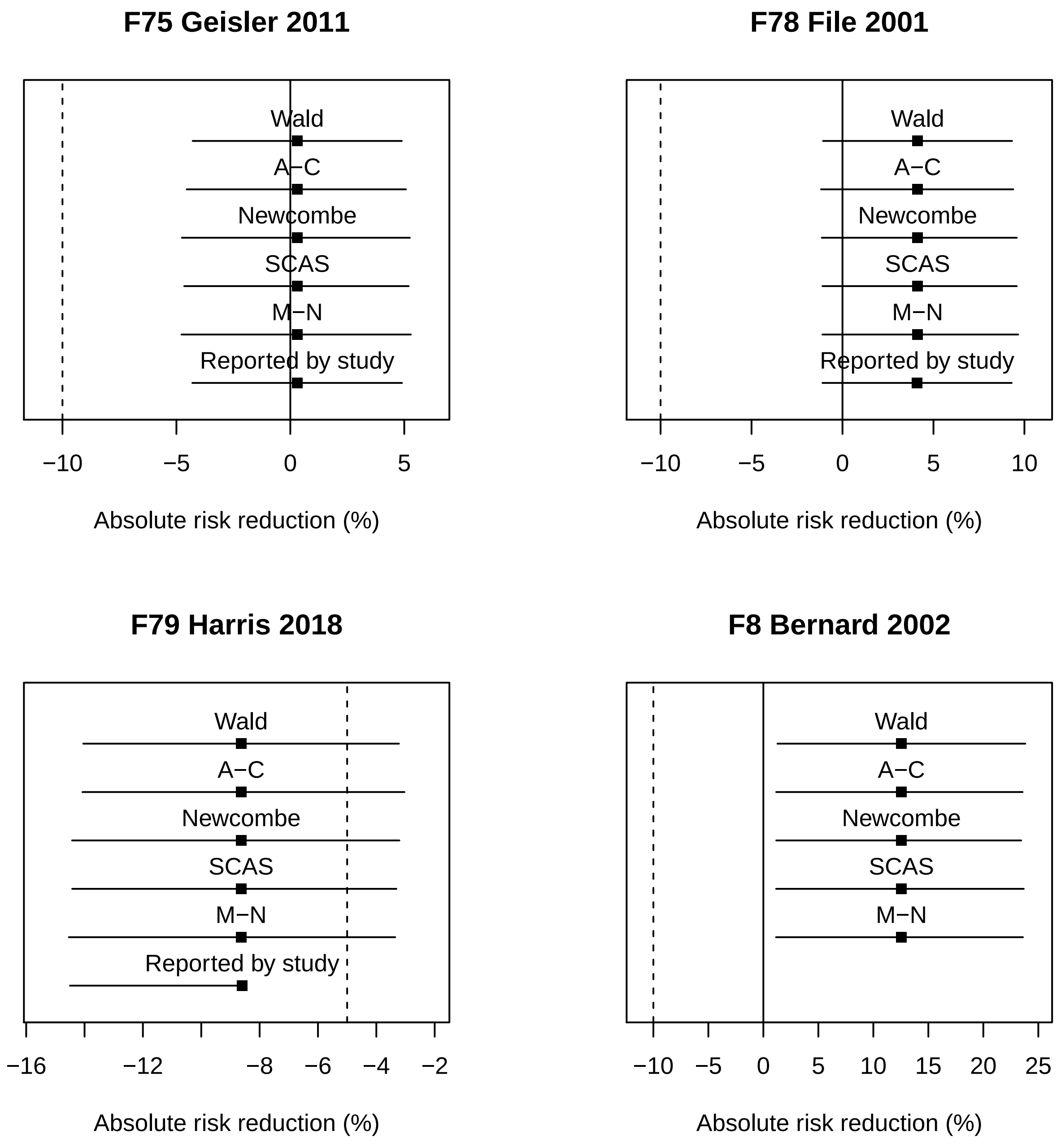
F75 Geisler 2011
Wald
A−C
Newcombe
SCAS
M−N
Reported by study
−10
−5
0
5
Absolute risk reduction (%)
F78 File 2001
Wald
A−C
Newcombe
SCAS
M−N
Reported by study
−10
−5
0
5
10
Absolute risk reduction (%)
F79 Harris 2018
Wald
A−C
Newcombe
SCAS
M−N
Reported by study
−16
−12
−8
−6
−4
−2
Absolute risk reduction (%)
F8 Bernard 2002
Wald
A−C
Newcombe
SCAS
M−N
−10
−5
0
5
10
15
20
25
Absolute risk reduction (%)

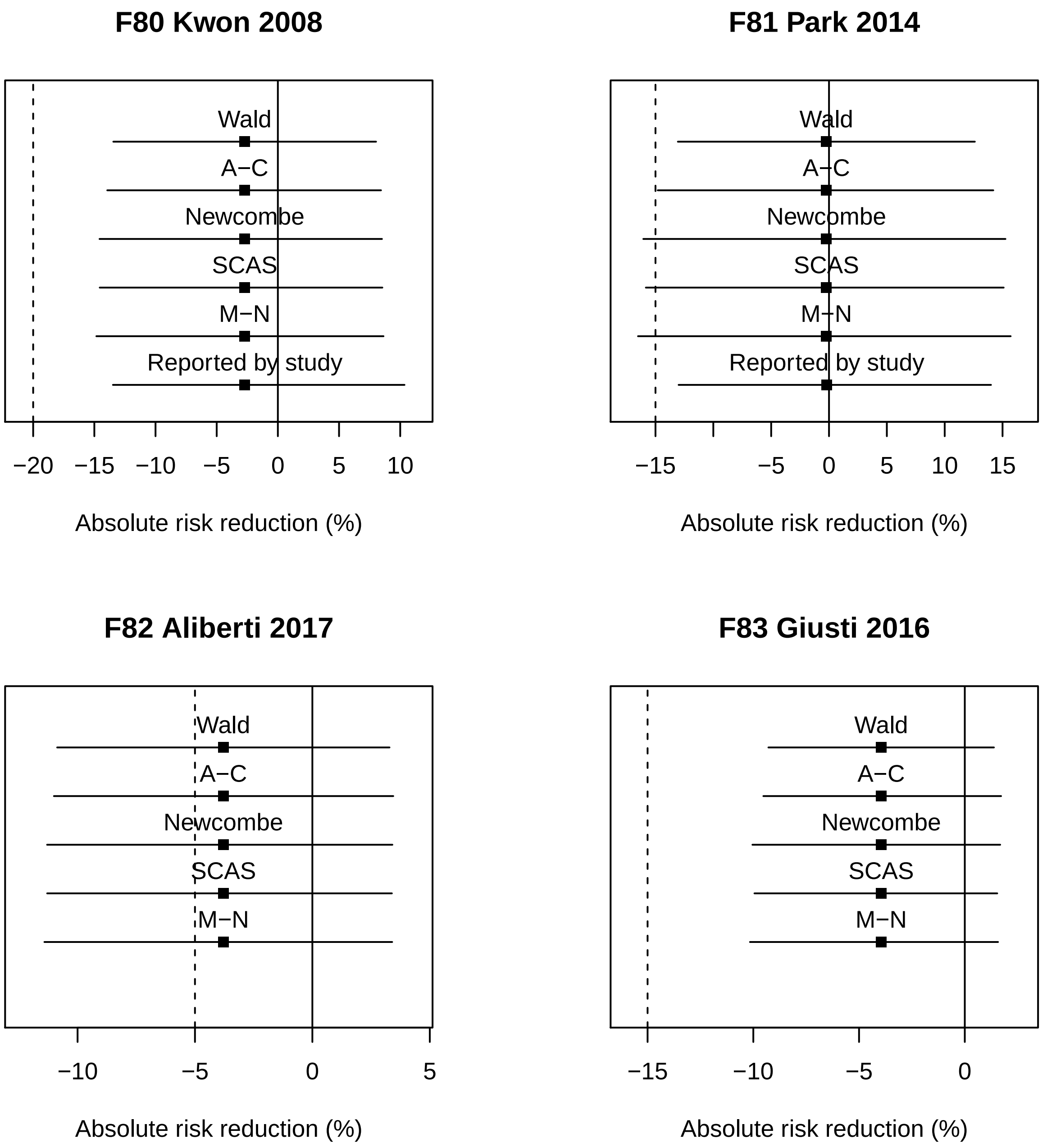
F80 Kwon 2008
Wald
A−C
Newcombe
SCAS
M−N
Reported by study
−20
−15
−10
−5
0
5
10
Absolute risk reduction (%)
F81 Park 2014
Wald
A−C
Newcombe
SCAS
M−N
Reported by study
−15
−5
0
5
10
15
Absolute risk reduction (%)
F82 Aliberti 2017
Wald
A−C
Newcombe
SCAS
M−N
−10
−5
0
5
Absolute risk reduction (%)
F83 Giusti 2016
Wald
A−C
Newcombe
SCAS
M−N
−15
−10
−5
0
Absolute risk reduction (%)

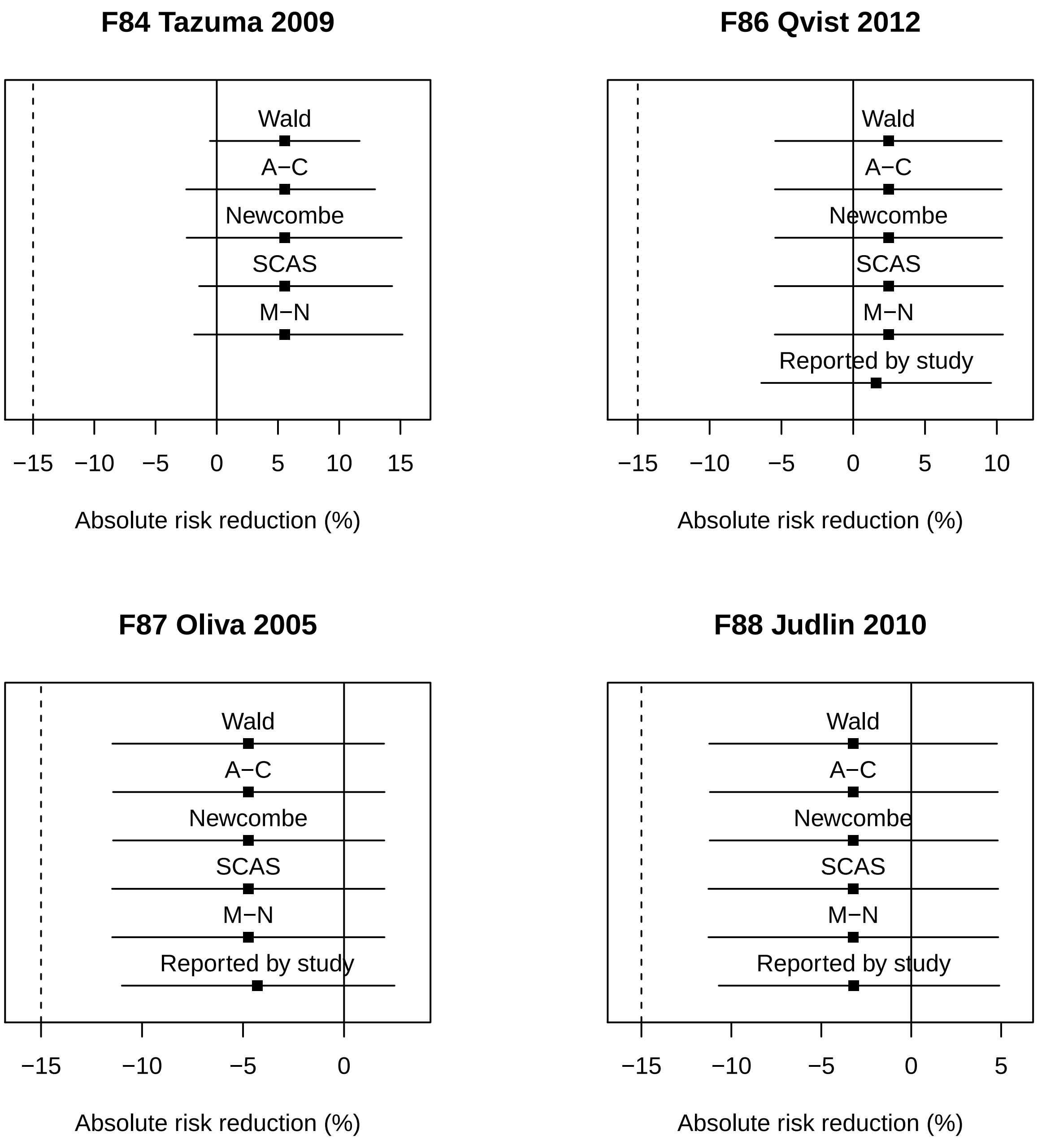

F84 Tazuma 2009
Wald
A−C
Newcombe
SCAS
M−N
−15
−10
−5
0
5
10
15
Absolute risk reduction (%)
F86 Qvist 2012
Wald
A−C
Newcombe
SCAS
M−N
Reported by study
−15
−10
−5
0
5
10
Absolute risk reduction (%)
F87 Oliva 2005
Wald
A−C
Newcombe
SCAS
M−N
Reported by study
−15
−10
−5
0
Absolute risk reduction (%)
F88 Judlin 2010
Wald
A−C
Newcombe
SCAS
M−N
Reported by study
−15
−10
−5
0
5
Absolute risk reduction (%)

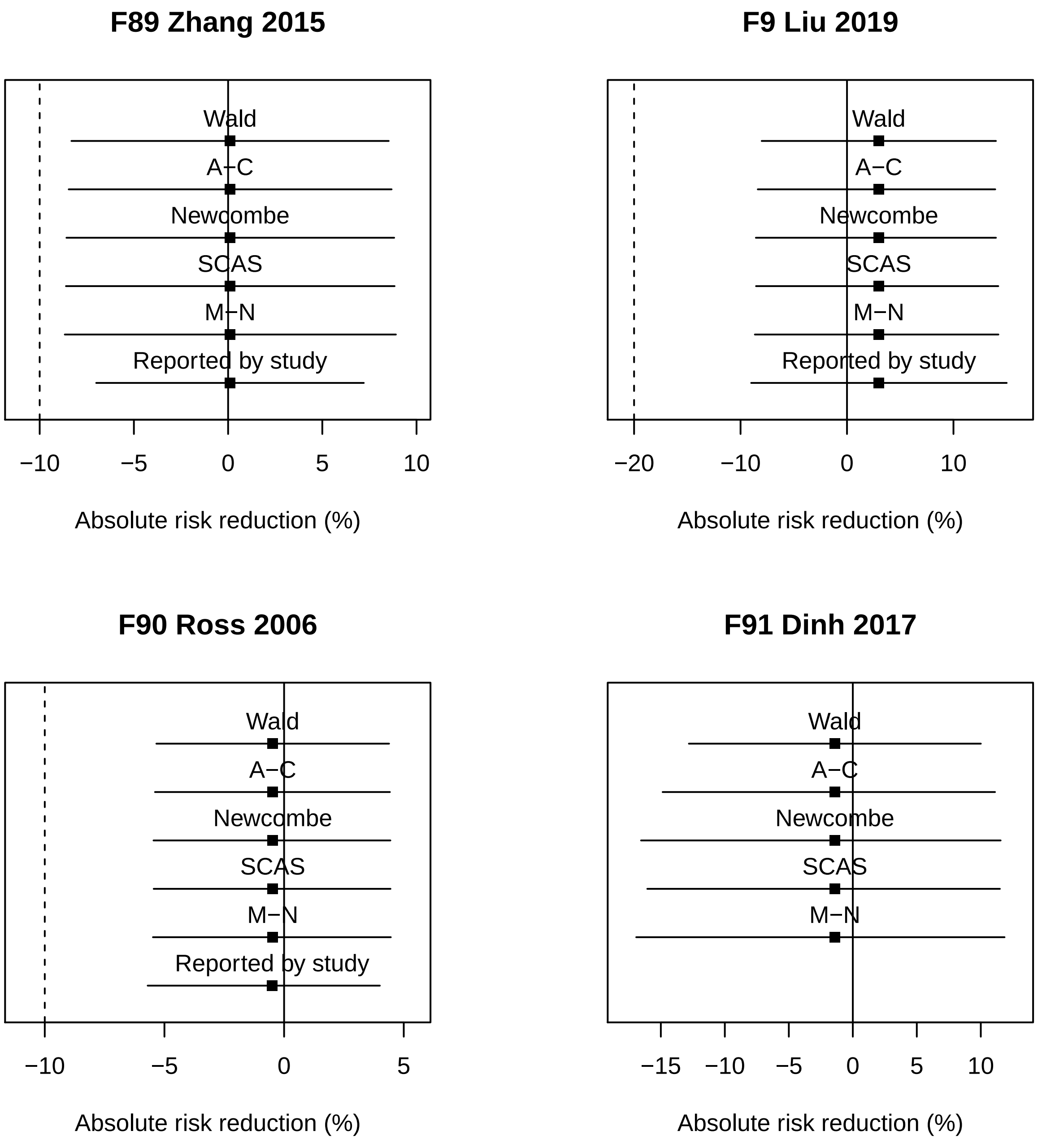
F89 Zhang 2015
Wald
A−C
Newcombe
SCAS
M−N
Reported by study
−10
−5
0
5
10
Absolute risk reduction (%)
F9 Liu 2019
Wald
A−C
Newcombe
SCAS
M−N
Reported by study
−20
−10
0
10
Absolute risk reduction (%)
F90 Ross 2006
Wald
A−C
Newcombe
SCAS
M−N
Reported by study
−10
−5
0
5
Absolute risk reduction (%)
F91 Dinh 2017
Wald
A−C
Newcombe
SCAS
M−N
−15
−10
−5
0
5
10
Absolute risk reduction (%)

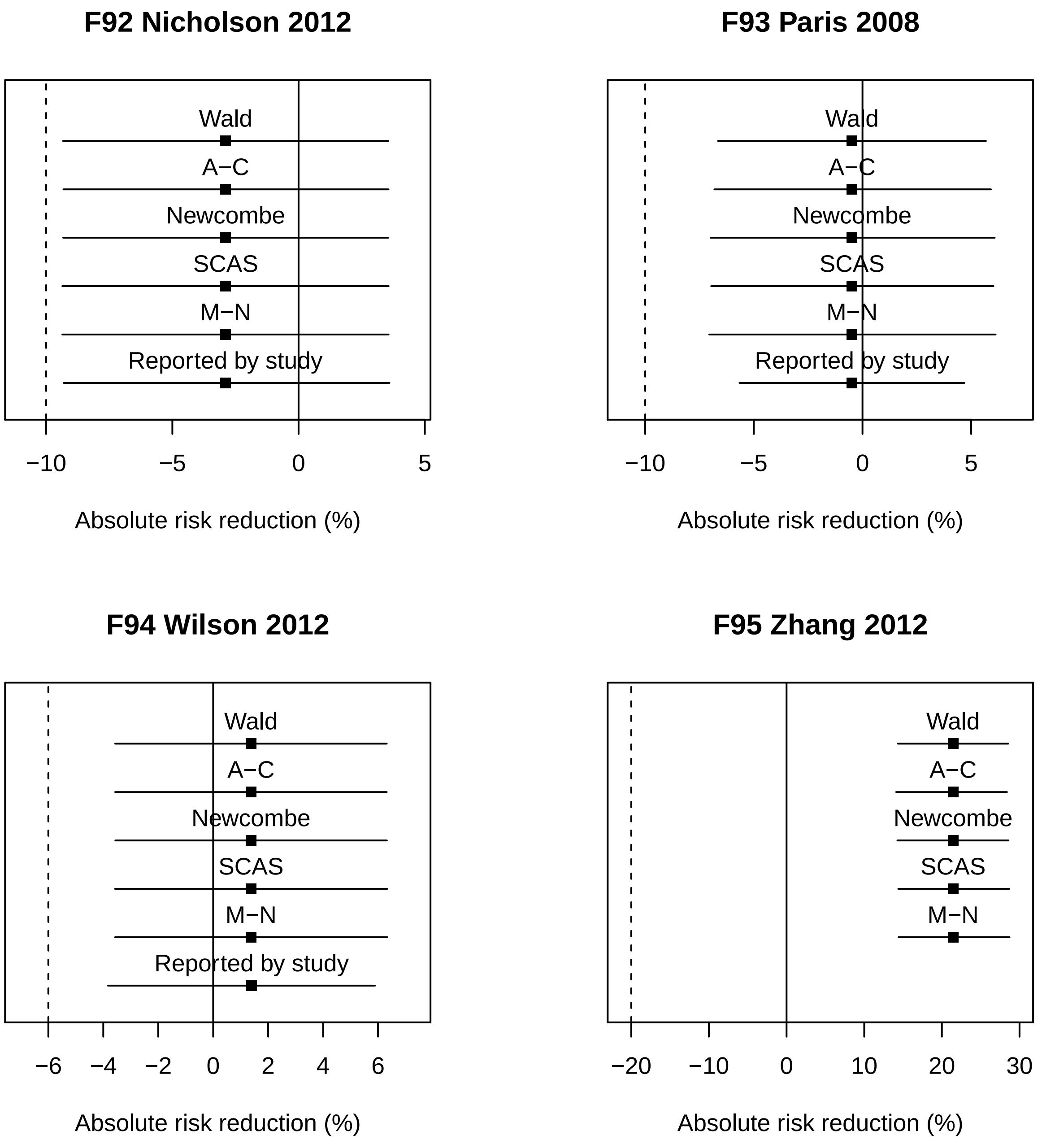

F92 Nicholson 2012
Wald
A−C
Newcombe
SCAS
M−N
Reported by study
−10
−5
0
5
Absolute risk reduction (%)
F93 Paris 2008
Wald
A−C
Newcombe
SCAS
M−N
Reported by study
−10
−5
0
5
Absolute risk reduction (%)
F94 Wilson 2012
Wald
A−C
Newcombe
SCAS
M−N
Reported by study
−6
−4
−2
0
2
4
6
Absolute risk reduction (%)
F95 Zhang 2012
Wald
A−C
Newcombe
SCAS
M−N
−20
−10
0
10
20
30
Absolute risk reduction (%)

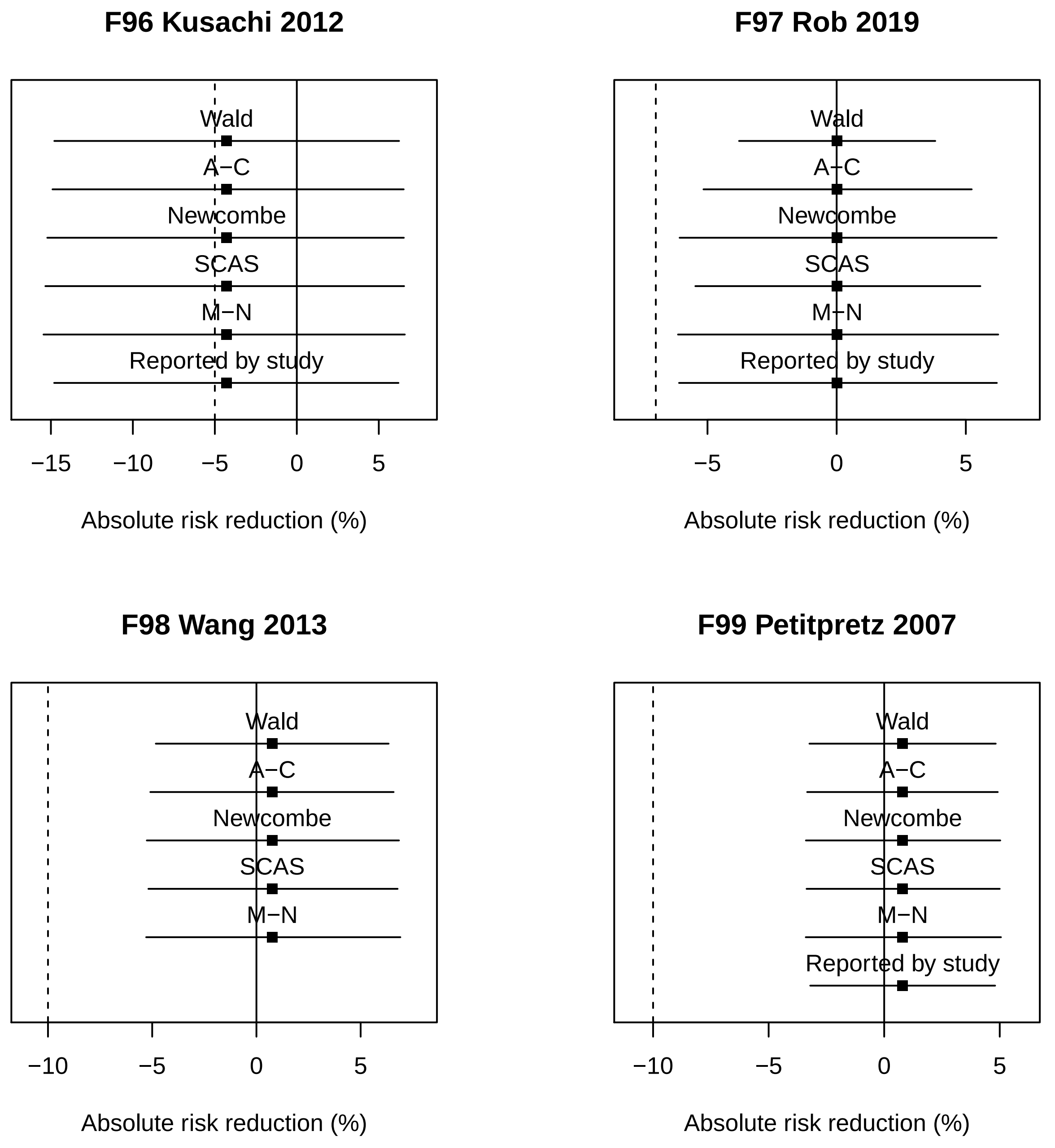

F96 Kusachi 2012
Wald
A−C
Newcombe
SCAS
M−N
Reported by study
−15
−10
−5
0
5
Absolute risk reduction (%)
F97 Rob 2019
Wald
A−C
Newcombe
SCAS
M−N
Reported by study
−5
0
5
Absolute risk reduction (%)
F98 Wang 2013
Wald
A−C
Newcombe
SCAS
M−N
−10
−5
0
5
Absolute risk reduction (%)
F99 Petitpretz 2007
Wald
A−C
Newcombe
SCAS
M−N
Reported by study
−10
−5
0
5
Absolute risk reduction (%)
